# The association between rainfall and human leptospirosis in Aotearoa New Zealand

**DOI:** 10.1017/S0950268825100423

**Published:** 2025-08-26

**Authors:** Toni Tana, Masako Wada, Jackie Benschop, Emilie Vallee

**Affiliations:** 1 Surveillance and Incursion Investigation, Ministry for Primary Industries, Wallaceville, New Zealand; 2 Tāwharau Ora, School of Veterinary Science, Massey University, Palmerston North, New Zealand

**Keywords:** leptospirosis, one health, rainfall, climate, zoonosis, surveillance, serovar

## Abstract

Leptospirosis remains a significant occupational zoonosis in New Zealand, and emerging serovar shifts warrant a closer examination of climate-related transmission pathways. This study aimed to examine whether total monthly rainfall is associated with reported leptospirosis in humans in New Zealand. Poisson and negative binomial models were developed to examine the relationship between rainfall at 0-, 1-, 2-, and 3-month lags and the incidence of leptospirosis during the month of the report. Total monthly rainfall was positively associated with the occurrence of human leptospirosis in the following month by a factor of 1.017 (95% CI: 1.007–1.026), 1.023 at the 2-month lag (95% CI:1.013–1.032), and 1.018 at the 3-month lag (95% CI: 1.009–1.028) for every additional cm of rainfall. Variation was present in the magnitude of association for each of the individual serovars considered, suggesting different exposure pathways. Assuming that the observed associations are causal, this study supports that additional human cases are likely to occur associated with increased levels of rainfall. This provides the first evidence for including rainfall in a leptospirosis early warning system and to design targeted communication and prevention measures and provide resource allocation, particularly after heavy rainfall in New Zealand.

## Introduction

Found worldwide, leptospirosis is caused by infection with any of the pathogenic species of the bacterial genus *Leptospira* [1, 2]. Infection typically occurs via contact with urine contaminated with *Leptospira* shed from the kidneys of infected animals. Indirect transmission also occurs through exposure to urine-contaminated water, food, or soil, as pathogenic *Leptospira* are able to survive in fresh water and in moist soil for days to weeks [3, 4]. In humans, the disease varies in severity, from a sub-clinical infection or mild clinical signs in the majority of people to fatal infections when renal, hepatic, or pulmonary failure occurs [2, 5]. Chronic symptoms are reported to affect approximately 30% of people and can last for months to years [6].

Leptospirosis is an important public human concern in New Zealand. Despite control efforts, resulting in a significant decline from 30 cases per 100,000 people in the early 1970s to 2.1 cases per 100,000 people in 2020 [7–9], leptospirosis remains one of New Zealand’s most notified non-foodborne zoonoses. It is predominantly an occupationally acquired disease in New Zealand; a hazard for those in contact with livestock, such as through farming or working in meat processing facilities [7, 8]. Historically, the serovars most commonly notified in humans in New Zealand were *Leptospira borgpetersenii* serovar Hardjo type hardjo-bovis (Hardjo) and *Leptospira interrogans* serovar Pomona (Pomona). However, the incidence of these serovars in notified cases has been declining since the early 1990s, likely as a result of the implementation of vaccination in the dairy industry [7, 10], while, more recently, *L. borgpetersenii* serovar Ballum (Ballum) has emerged as an important serovar, becoming the most common confirmed serovar for the first time in 2010 [11]. The association of serovar Balum with rodent maintenance hosts suggests alternative transmission pathways may be increasingly occurring in New Zealand [12].

Rainfall and flooding events are recognized as important determinants for leptospirosis in humans overseas, with a positive association between rainfall and leptospirosis previously being reported in numerous studies [13–18]. Surface water and flooding events can bring humans into contact with *Leptospira*-contaminated water, as well as facilitate the dispersal of *Leptospira* in the environment through surface run-off into bodies of water or onto other areas of land. An emerging theory proposes that heavy rain re-suspends *Leptospira* together with soil particles so that the bacteria are carried to surface water, from which humans and other animals are exposed [4, 19]. Wet weather and heavy rain can also encourage rodents into human dwellings and farm buildings, creating a risk of exposure to *Leptospira*-contaminated urine when addressing rodent infestations or handling contaminated animal forage or bedding.

No recent work has explored the association between the incidence of human cases in New Zealand and rainfall. Gaining an understanding of this association may inform the recognition of risk circumstances for increased disease occurrence. Identifying whether climatic conditions create an increased risk of disease occurrence could provide a mechanism for health officials to implement preventative measures to minimize disease and to enable resources to be saved. This study aimed to examine whether rainfall is associated with the reported occurrence of leptospirosis in humans in New Zealand.

## Methods

### Data sources

#### Reported cases of leptospirosis

Leptospirosis is a notifiable disease in humans in New Zealand. Health practitioners (medical practitioners, nurses, or midwives) and the people in charge of medical laboratories, officially, must notify actual and suspected cases of leptospirosis to their local public health units’(PHUs) medical officer of health within regional health subdivisions called district health boards (DHBs).

The reported cases are interviewed by staff in PHUs using a standardized questionnaire (Supplementary Material S1) and centrally collated from the PHUs into New Zealand’s national notifiable disease database managed by the Institute of Environmental Science and Research (ESR). For this investigation, leptospirosis case data for the period from 1 January 1999 to 31 December 2017 was obtained. This case series has previously been described by Nisa et al. [7].

A case definition, case classifications, and the requirements for laboratory definitive evidence for a confirmed case of leptospirosis are maintained by the New Zealand Ministry of Health [20] (see Supplementary Material S1). The infecting serovar was determined by ESR when the case definition (Supplementary Material S1) was met. When the case definition was met for more than one serovar, no serovar was attributed to the case.

Information from each case utilized for this analysis consisted of: the case status as either confirmed, probable, or unknown; the date reported; location of the case at the DHB and the New Zealand second tier of local government Territorial Authority (TA) level; the infecting serovar; and whether the case had a history of overseas travel within the incubation period for infection. Personal information was removed before the data was provided at an aggregated level by ESR.

This study was recorded on the Massey University, New Zealand human ethics database as a low-risk notification (Ethics Notification Number: 4000022554, May 2020).

#### Rainfall data

Climatic conditions vary considerably across New Zealand from warm subtropical in the northern part of the North Island to cool temperate in the south of the South Island and alpine in mountainous areas [21].

Climate stations present in the New Zealand National Institute of Water and Atmospheric Research (NIWA) National Climate Database that were collecting rainfall data over the entire study period (1999–2017) were identified through the web-based interface CliFlo (https://cliflo.niwa.co.nz/).

**Figure 1.**
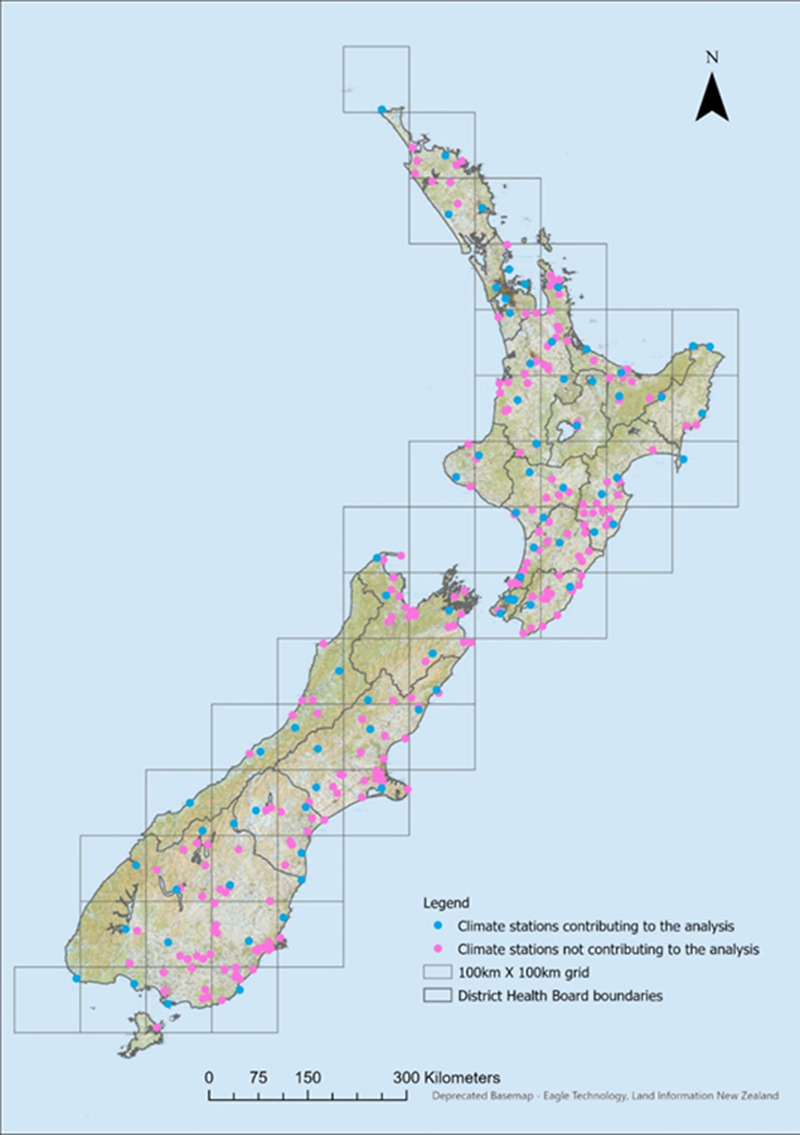
Map demonstrating the process of climate station selection, the position of all the climate stations eligible for inclusion in the analysis, and the overlayed 100-by-100 km grid used to select the climate stations that contributed to the study (blue dots). Map lines delineate study areas and do not necessarily depict accepted national boundaries.

The Global Positioning System positions of 283 climate stations were mapped to visualize the distribution of the stations across New Zealand (Figure 1). Because of the uneven geographic distribution of climate stations, inclusion of measurements from all existing climate stations would have resulted in the disproportionate contribution of rainfall measurements from the areas with the higher density of climate stations. To avoid this, a 100-by-100 km grid was used and overlayed on the map of climate stations (Figure 1). This enabled selection of climate stations that were more or less evenly geographically spread in a DHB. Within each section of a grid square intersecting a DHB, the most central climate station was selected for inclusion in the study (Supplementary Material S2). Where two stations were a similar distance from the central point of a grid section, the station with the higher number of observations was used. For DHBs with two or fewer stations present, all stations were included. A total of 76 climate stations were used in the study (Figure 1).

#### Denominator and spatial data

Publicly available human population data for each DHB for the years 1999–2017 (Supplementary Material S2) were obtained from StatsNZ: Tatauranga [22]. Animal population data by TA for the New Zealand Agricultural census years of 2002, 2007, 2012, and 2017 (Supplementary Material S2) were obtained from the New Zealand Agricultural Production Survey [23]. The geographical boundaries of the DHBs vary from those of the TAs (Supplementary Material S2), and both the DHB and TAs spatial polygons were obtained from Koordinates Limited [24].

### Statistical analysis

All statistical analyses were carried out using R Version 4.0.3 [25].

The outcome variable in this study was the reported incidence rate of leptospirosis in humans per month for each DHB. The exposures of interest were the total monthly rainfall (cm) for each DHB (Total rainfall), with 0-, 1-, 2-, and 3-month lags counting backward from the current month during the study period.

#### Data preparation

##### Reported cases of leptospirosis

Cases that were considered unrelated to domestic exposure or of an unknown case status were excluded from the study. Out of the 1,695 cases provided by ESR for the study period, 71 records were excluded due to the cases having a confirmed history of overseas travel during the relevant incubation period and 67 cases were excluded as they were not a confirmed or probable case as defined by MoH. A further 13 cases were excluded as they were attributed to serovars considered exotic to New Zealand, namely: *L. interrogans* serovar Australis (*n* = 2), *L. interrogans* serovar Bratislava (*n* = 1), *L. interrogans* serovar Canicola (*n* = 8), and *L. interrogans* serovar Grippotyphosa (*n* = 2). In total, 1,544 reported cases entered the analysis.

##### Rainfall, human population, and season

The total monthly rainfall values (‘Total Rainfall’) obtained from CliFlo were converted to centimetres and averaged across the climate stations for each DHB. The total rainfall data were merged with the case dataset using DHB and month-year as joining fields.

In addition, total rainfall with lags of 1, 2, and 3 months were created, and along with human population data and season were merged with the case dataset.

The season in which a report occurred was defined from the calendar month in each record (Autumn: March–May; Winter: June–August; Spring: September–November; Summer: December–February).

##### Animal density

To obtain the animal density in each DHB, the animal population of each TA was interpolated from data on the years 2002, 2007, 2012, and 2017 to estimate the populations for each TA for the missing intervening years between 2002 and 2017. For the years 1999, 2000, and 2001, the population recorded in the 2002 census was used. Animal numbers were obtained for dairy cattle, beef cattle, sheep, goats, pigs, horses, llama, and alpaca for each TA. The totals from all species were combined to create the animal population for the TA in a particular year.

The animal population data available at the TA level were then aggregated at the DHB level to obtain the total animal population for each DHB. Finally, the animal population in each DHB was divided by the number of squared kilometres in the DHB to derive the animal density.

#### Final dataset

Four DHB-month entries in the dataset without rainfall information (due to absent measurements from climate stations) were removed. This resulted in no loss of cases from the dataset. In total 4,556 DHB-month records were analysed.

#### Regression analysis

Univariable analyses of the explanatory variables (season, animal density, DHB, total rainfall at zero (month of the report), 1-, 2-, and 3-month lags, and year were performed to assess their crude association with incidence rate of leptospirosis.

Variables significant in univariable models at a screening threshold p-value of <0.20 by likelihood ratio test statistic were included in the initial multivariable models. In New Zealand, season directly influences the amount of rainfall and the manifestation of other climatic variables. Season also influences occupational exposure events associated with human leptospirosis such as calving and milking, and the volume and ages of animals processed at abattoirs. Therefore, season was retained as a variable in the model regardless of its significance because it was considered likely to be a confounding variable for the association of rainfall and the incidence of human leptospirosis.

Poisson and, when overdispersal was present (dispersal parameter >1.05 [26]), negative binomial regression models were built for case numbers per month per DHB with an offset for human population per DHB.

Variables were removed from the model by backward stepwise elimination until all predictor variables had a p-value less than 0.05 by the likelihood ratio test. Explanatory variables that were excluded at the initial screening stage were tested again for inclusion in the final model. Variables were retained if a rainfall variable’s beta coefficient changed more than 15% by exclusion of these terms, regardless of their significance. The effect of year was included in the model as a cubic term when a non-linear relationship between year and incidence was present. Two-way interactions between rainfall and season, and between rainfall and DHB were tested because season and DHB were considered likely to modify the effect of rainfall. The model fit was evaluated by visually assessing the deviance and standardized residual distributions and consideration of evidence of overdispersal. Identification of the most appropriate model was achieved by examination of the models’ AIC estimate and confirmed by a log likelihood test between the Poisson and the negative binomial models.

The modelling process was repeated for each of the three most commonly reported serovars (Hardjo, Pomona, and Ballum, [7]).

The general form of the full models before variable selection is presented in equation (1):(1)

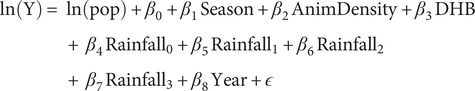

Where:

- Y is the expected number of leptospirosis cases in a given DHB and month.
- ln(pop) is the offset term for human population per DHB.
- AnimDensity is the animal density for a given DHB.

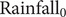
 to 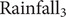 are total rainfall at 0 to 3-month lags.

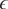
 is the error term.

## Results

### Descriptive analysis of the data

**Figure 2.**
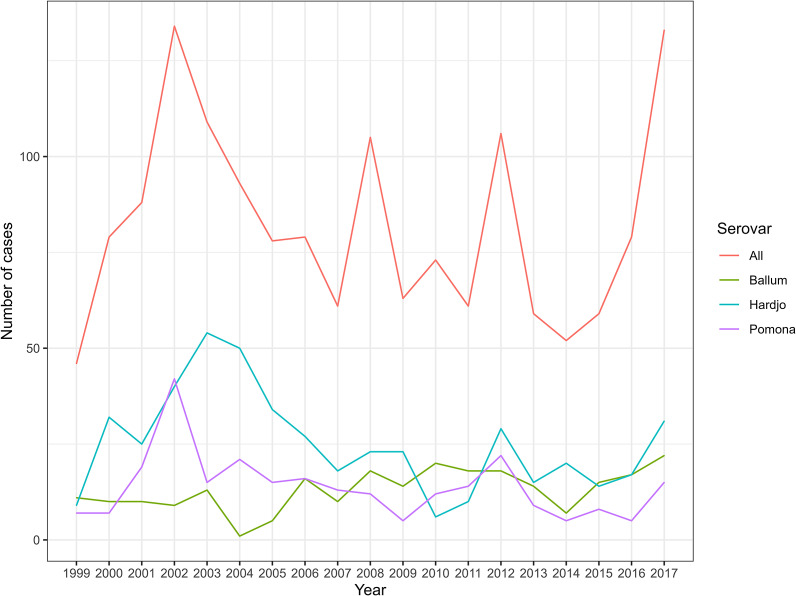
Annual number of reported leptospirosis cases and the numbers of the three most commonly identified individual serovars in New Zealand between 1999 and 2017.

During the study period, there were 1,544 confirmed (*n* = 1,445) or probable (*n* = 99) cases of human leptospirosis reported that were not associated with an exotic serovar or a recognized history of overseas travel. Five serovars were represented in the data set: Hardjo (*n* = 477, 42.5% out of 1,122 cases for which a serovar was attributed), Pomona (*n* = 262, 23.4%), Ballum (*n* = 248, 22.1%), *L. borgpetersenii* serovar Tarassovi (*n* = 106, 9.4%)*, L. interrogans* serovar Copenhageni (*n* = 29, 2.6%), along with 422 cases for which no serovar was identified. The annual number of reported cases in total in the final analysed dataset, and for the top three serovars considered individually, are presented in Figure 2.

### Leptospirosis incidence

#### Summary statistics

Using the cases included in the study, the average annual incidence over the period was 1.92 cases/100000 people, with a standard deviation (SD) of 0.63 and a range of 1.15 to 3.39 cases/100000 people. The mean monthly incidence of reported cases of leptospirosis for the whole of New Zealand in the study period was 0.16 cases/100000 people (SD = 0.09, range 0–0.44). The mean monthly incidence within a DHB ranged from 0.01 cases/100000 people (SD = 0.04, range 0–0.26) in the Auckland DHB to 0.64 cases/100000 people (SD = 1.47, range 0–6.37) in the West Coast DHB.

Mean total monthly rainfall, the minimum total monthly rainfall, and the maximum total monthly rainfall varied between DHBs from 6.78 to 26.32 cm, 0.28 to 5.01 cm, and 21.42 to 57.07 cm, respectively.

### Statistical analysis

#### All leptospirosis cases

In the univariable regression analysis animal density, DHB, year, and total rainfall at 0-, 1-, 2-, and 3-monthly lags were significantly associated with the incidence rate of reported cases of any *Leptospira* serovar (Table 1). Season was not significant on univariable analysis.

**Table 1.** Estimated coefficients and incidence rate ratios (IRR) of explanatory variables in a multivariable negative binomial regression model with the incidence rate of reported leptospirosis cases as the outcome variable

			IRR confidence interval		
Variable name	Beta coefficient	Incidence rate ratio (IRR)	2.5%	7.5%	Wald test p-value
Intercept	−13.122				
Total monthly rainfall (cm)					
Total rainfall_1^a^	0.017	1.017	1.007	1.026	**<0.001**
Total rainfall_2^b^	0.023	1.023	1.013	1.032	**<0.001**
Total rainfall_3^c^	0.018	1.018	1.009	1.028	**<0.001**
Season					
Autumn	Reference				
Spring	−0.231	0.794	0.673	0.935	**<0.01**
Summer	−0.151	0.860	0.734	1.008	0.06
Winter	−0.265	0.767	0.650	0.906	**<0.01**
District health board					
Northland	Reference				
Auckland	−3.990	0.018	0.007	0.039	**<0.001**
Bay of Plenty	−0.806	0.447	0.325	0.611	**<0.001**
Canterbury	−1.092	0.336	0.252	0.447	**<0.001**
Capital and Coast	−2.937	0.053	0.026	0.097	**<0.001**
counties Manukau	−2.178	0.113	0.075	0.166	**<0.001**
Hawke’s Bay	0.695	2.003	1.543	2.609	**<0.001**
Hutt Valley	−2.754	0.064	0.027	0.128	**<0.001**
Lakes	−0.973	0.378	0.240	0.576	**<0.001**
MidCentral	0.112	1.118	0.847	1.478	0.43
Nelson Marlborough	−0.415	0.660	0.479	0.905	**<0.05**
South Canterbury	0.166	1.181	0.791	1.729	0.4
Southern	−0.887	0.412	0.307	0.552	**<0.001**
Tairawhiti	0.367	1.443	1.026	2.012	**<0.05**
Taranaki	−0.285	0.752	0.545	1.032	0.08
Waikato	−0.081	0.922	0.72	1.185	0.52
Wairarapa	0.160	1.173	0.756	1.769	0.46
Waitemata	−2.373	0.093	0.062	0.137	**<0.001**
West Coast	−0.261	0.77	0.488	1.200	0.26
Whanganui	0.439	1.551	1.117	2.140	**<0.01**
Year					
Polynomial (year,3) 1^d^	−7.311	0.001	0.000	0.030	**<0.001**
Polynomial (year,3) 2^d^	−0.065	0.937	0.021	41.029	0.97
Polynomial (year,3) 3^d^	11.307	81,366.82	1847.028	3,658,399	**<0.001**

a Total rainfall_1 is the total monthly rainfall in cm the month prior to the month a case is reported in.

b Total rainfall_2 is the total monthly rainfall in cm in the month, 2 months prior to the month a case is reported in.

c Total rainfall_3 is the total monthly rainfall in cm in the month, 3 months prior to the month a case is reported in.

d Year as a cuboid polynomial variable.

On multivariable analysis, after accounting for season, year, and DHB, the reporting of leptospirosis was significantly associated with total rainfall at all three lags. Total rainfall at the zero-lag, although significant on univariable analysis, was not significant in the multiple model and was eliminated. On average, every centimetre increase in rainfall in a month was associated with an increase in the expected number of reported cases by a factor of 1.017 (95% CI: 1.007–1.026), 1.023 (95% CI: 1.013–1.032), and 1.018 (95% CI: 1.009–1.028) in the following month, at the two-month lag, and three-month lag, respectively (Table 1). The 1-month lag IRR of 1.017 suggests that on average an additional 1 cm of rainfall in a month in a given DHB, was associated with an increase in the incidence rate ratio of reported leptospirosis by a factor of 1.017 or 1.7% in that DHB in the following month (Table 1).

#### Hardjo serovar

On multivariable analysis, accounting for season, year, and DHB, the number of cases of reported Hardjo was significantly associated with all four rainfall variables. On average, every 1 cm increase in rainfall in a month was associated with an increase in the expected number of reported Hardjo cases by a factor of 1.017 (95% CI: 1.001–1.033) in the month of the report, and by 1.019 (95% CI: 1.003–1.035), 1.025 (95% CI: 1.010–1.041), and 1.020 (95% CI: 1.004–1.036) in the following month and at a lag of 2 months, and 3 months, respectively (Table 2). An interaction term between DHB and total rainfall at a 2-month lag was significant, suggesting that the association between rainfall 2 months earlier on the incidence rate of Hardjo cases varied by DHB. With the inclusion of the interaction term, rainfall at the 2-month lag had a significant effect in five DHBs. The IRR of the effect of rainfall at the 2-month lag with inclusion of the interaction term ranged from 0.741 in South Canterbury to 1.118 in Wairarapa (Table 3).

**Table 2.** Table of the incidence rate ratios (IRR) of explanatory variables in Poisson generalized linear models with the incidence rate of reported Hardjo, Pomona, and Ballum cases included in the analyses in New Zealand between 1 January 1999 and 31 December 2017 as the outcome variable

	Hardjo				Pomona				Ballum			
		IRR confidence interval				IRR confidence interval				IRR confidence interval		
Variable name	Incidence rate ratio	2.5%	97.5%	Wald test p-value	Incidence rate ratio	2.5%	97.5%	Wald test p-value	Incidence rate ratio	2.5%	97.5%	Wald test p-value
Animal density (animals/km^2^)					0.981	0.970	0.992	<0.01				
Total rainfall^a^	1.017	1.001	1.033	<0.05								
Total rainfall_1^b^	1.019	1.003	1.035	<0.05					1.025	1.005	1.044	<0.05
Total rainfall_2^c^	1.025	1.010	1.041	<0.01								
Total rainfall_3^d^	1.020	1.004	1.036	<0.05	1.024	1.003	1.045	<0.05				
Season												
Autumn												
Spring	1.241	0.957	1.610	0.10	0.862	0.610	1.218	0.40	0.485	0.334	0.705	<0.001
Summer	0.999	0.762	1.311	1.00	1.088	0.786	1.507	0.61	0.595	0.417	0.847	<0.01
Winter	0.914	0.692	1.206	0.53	0.741	0.517	1.062	0.10	0.830	0.602	1.145	0.26
District Health Board												
Northland												
Auckland	0.000^e^	0.000	3.21e+302	0.96	0.000^e^	0.000	2.423e+304	0.96	0.028	0.009	0.091	<0.001
Bay of Plenty	0.136	0.061	0.307	<0.001	0.168	0.052	0.545	<0.01	0.247	0.131	0.462	<0.001
Canterbury	0.470	0.301	0.732	<0.001	3.130	0.688	14.230	0.14	0.131	0.072	0.238	<0.001
Capital and Coast	0.019	0.003	0.135	<0.001	0.042	0.005	0.335	<0.01	0.046	0.014	0.149	<0.001
Counties Manukau	0.088	0.041	0.191	<0.001	0.206	0.020	2.072	0.18	0.018	0.004	0.075	<0.001
Hawke’s Bay	3.179	2.133	4.738	<0.001	438.071	32.595	5,887.567	<0.001	0.478	0.270	0.848	<0.05
Hutt Valley	0.06	0.014	0.248	<0.001	0.047	0.008	0.279	<0.001	0.058	0.014	0.240	<0.001
Lakes	0.096	0.023	0.400	<0.01	0.415	0.090	1.913	0.26	0.125	0.039	0.406	<0.01
MidCentral	1.555	1.015	2.383	<0.05	1,271.689	39.598	40,839.762	<0.001	0.307	0.160	0.586	<0.001
Nelson Marlborough	0.611	0.354	1.056	0.08	0.267	0.084	0.851	<0.05	0.790	0.484	1.291	0.35
South Canterbury	1.827	0.991	3.367	0.05	9.108	2.448	33.887	<0.001	0.490	0.206	1.165	0.11
Southern	0.682	0.444	1.048	0.08	1.771	0.404	7.770	0.45	0.228	0.129	0.402	<0.001
Tairawhiti	0.803	0.405	1.588	0.53	71.515	13.139	389.234	<0.001	0.245	0.076	0.793	<0.05
Taranaki	0.517	0.285	0.936	<0.05	3.255	0.862	12.288	0.08	0.592	0.335	1.049	0.07
Waikato	0.751	0.501	1.126	0.17	18.662	4.030	86.422	<0.001	0.429	0.276	0.668	<0.001
Wairarapa	1.985	1.058	3.724	<0.05	130.384	8.446	2012.800	<0.001	0.761	0.339	1.709	0.51
Waitemata	0.009	0.001	0.067	<0.001	0.049	0.006	0.385	<0.01	0.024	0.007	0.078	<0.001
West Coast	0.502	0.220	1.146	0.10	0.049	0.004	0.534	<0.05	0.864	0.403	1.853	0.71
Whanganui	1.471	0.840	2.574	0.18	875.557	32.611	23,507.625	<0.001	0.608	0.294	1.258	0.18
Year												
Polynomial (year, 3) 1^f^	0.000	0.000	0.000	<0.001	0.000	0.000	0.000	<0.001				
Polynomial (year, 3) 2^f^	0.176	0.000	86.981	0.58	0.000	0.000	0.011	<0.01				
Polynomial (year, 3) 3^f^	4.96e+09	8.4e+6	2.91e+12	<0.001	5.906e+7	9,471.625	3.682e+11	<0.001				
Year (continuous)^g^									1.030	1.007	1.054	<0.05

a *Total rainfall* is the total monthly rainfall in cm during the month a case is reported in.

b *Total rainfall_1* is the total monthly rainfall in cm in the month, prior to the month a case is reported in.

c *Total rainfall_2* is the total monthly rainfall in cm in the month, 2 months prior to the month a case is reported in.

d *Total rainfall_3* is the total monthly rainfall in cm in the month, 3 months prior to the month a case is reported in.

e The estimated coefficient and wide confidence interval are a consequence of no cases being reported in the Auckland DHB. The findings are not considered a concern to the validity of the results for the rainfall variable as model was able to converge and we are not interpreting the IRRs of the DHBs.

f Year as a cuboid polynomial variable.

g Year as a linear variable.

**Table 3.** Association between the total monthly rainfall lagged by two-months and the incidence rate of reported cases of leptospirosis attributed to Hardjo that were included in the analysis by District Health Board, based on the fitted regression model with an interaction term between DHB and total monthly rainfall at the two-month lag

District Health Board	Coefficient	Incidence Rate Ratio (IRR)	IRR confidence interval – lower limit	IRR confidence interval – upper limit	Wald test p-value
Auckland	−0.007	0.992	2.497e–48	3.946 + 47	1.00
Bay of Plenty	0.080	1.083	1.003	1.170	**<0.05**
Canterbury	−0.002	0.996	0.937	1.062	0.939
Capital and Coast	0.073	1.076	0.815	1.421	0.61
Counties Manukau	−0.025	0.975	0.849	1.120	0.72
Hawke’s Bay	0.056	1.057	1.019	1.097	**<0.01**
Hutt Valley	−0.019	0.981	0.783	1.228	0.87
Lakes	0.046	1.047	0.850	1.290	0.66
MidCentral	0.044	1.045	0.997	1.095	0.07
Nelson Marlborough	0.080	1.083	1.015	1.155	**<0.05**
Northland	0.016	1.016	0.968	1.067	0.52
South Canterbury	−0.300	0.741	0.590	0.930	**<0.01**
Southern	−0.006	0.994	0.932	1.060	0.85
Tairawhiti	0.003	1.003	0.920	1.094	0.94
Taranaki	0.040	1.040	0.974	1.109	0.24
Waikato	0.019	1.019	0.984	1.056	0.29
Wairarapa	0.111	1.118	1.039	1.203	**<0.01**
Waitemata	0.110	1.116	0.848	1.469	0.43
West Coast	−0.012	0.988	0.939	1.040	0.65
Whanganui	−0.047	0.954	0.859	1.059	0.38

#### Pomona serovar

Animal density, year, DHB, and total rainfall at the 3-month lag were significantly associated with the incidence rate of reported cases of Pomona. Total rainfall at the zero-lag, although significant on univariable analysis, was not significant in the multiple model and was eliminated.

No interactions were present between total rainfall at the 3-month lag and season or the DHB. Accounting for animal density, season, year, and DHB, on average every 1 cm increased in rainfall in a month was associated with an increased in the expected number of reported cases 3 months later by a factor of 1.024 (95% CI: 1.003–1.045).

#### Ballum serovar

In the final multivariable Ballum Poisson model, year, season, DHB, and total rainfall lagged by 1 month were retained. No interactions were present between total rainfall at a lag of 1 month and season or the DHB. On average, a 1 cm increase in rainfall in a month was associated with an increase in the expected number of reported cases of Ballum by a factor of 1.025 (95% CI 1.005–1.044) in the following month.

## Discussion

This study investigated the relationship between rainfall and the reported occurrence of human leptospirosis in New Zealand over the period 1999–2017. The monthly incidence rate of leptospirosis in a DHB was found to have a significant positive association with total rainfall during each of the 3 months prior to the month the case was reported. However, variation was observed in the association between rainfall and reported cases when the serovars Hardjo, Pomona, and Ballum were examined individually.

Previous literature examining the association between rainfall and the reported occurrence of leptospirosis in humans has considered the occurrence at the genus level. Thus, our study is unique in its analysis at the serovar level. To our knowledge, the only other study identified that considered the role of rainfall on different *Leptospira* serovars was in non-human animals [27].

### All reported cases

When accounting for other variables in the model, every 1 cm increase in total rainfall was associated with increased incidence of reported leptospirosis by 1.7% (1-month lag), 2.3% (2-month lag), and 1.8% (3-month lag). The study findings indicate that rainfall is associated with an increase in the occurrence of reported leptospirosis in humans in New Zealand and that the impact of rainfall on reported cases would be observed cumulatively over lags of 3 months or possibly more.

Heavy rainfall events have been linked to outbreaks of leptospirosis overseas [5], and climate change is predicted to bring an increased frequency of extreme rainfall events to New Zealand [28]. While this study did not evaluate extreme events directly, the association between increased rainfall and reported leptospirosis signals the potential for rainfall to play an increasingly important role in the epidemiology of human leptospirosis. Anecdotal support of this was observed in 2023 when unusually high numbers of leptospirosis cases were notified in the Hawke’s Bay DHB after Cyclone Gabrielle [29].These results suggest that while livestock exposure remains the likely main pathway for human leptospirosis in New Zealand [30], localized outbreaks may become more frequent, driven by emerging exposure pathways like rainfall in this study or rodents identified in [30]. Local health facilities may need to adapt to a new pattern of occurrence, with localized increases of cases over a short time.

The study’s demonstration of positive associations between lagged effects of rainfall and leptospirosis incidence is consistent with the majority of overseas studies conducted in tropical climates [15, [31]–33]. Variable periods of lagged effects have been reported, with lags as short as 2 weeks and as long as 10 months [34, 35]. The strength of association observed in this study was not as strong as seen in the Republic of Korea [17], a temperate country. On the other hand, a recent study in the Netherlands [36], also a temperate country, found no association between human cases and rainfall. The diversity in the presence and strength of association between cases and rainfall could be due to methodological differences but also suggest a more complex pathway that would involve rainfall and other factors, including differences in rainfall measurement, environmental covariates, serovar and hosts present, health care systems, or mitigations in place (such as cattle vaccination) in New Zealand. These findings suggest early warning systems based on rainfall only for leptospirosis may have limited effectiveness in temperate climates like New Zealand, highlighting the need for additional research to inform surveillance approaches that incorporate multiple environmental and epidemiological factors rather than relying solely on precipitation patterns for disease prediction and prevention strategies.

### Models of individual serovars and implication on exposure pathways

Pomona cases were significantly associated with Total Rainfall only at the 3-month lag, while Ballum cases were associated only at the 1-month lag. In contrast, the reported occurrence Hardjo was significantly associated with increased total rainfall for all four rainfall variables considered, with regional variation observed in the 2-month lag effect across DHBs.

The presence of lagged effects of total rainfall on leptospirosis incidence rates is consistent with rainfall playing a role in the causal pathway for human leptospirosis through facilitating infection at or soon after rainfall. This aligns with the incubation period of human leptospirosis and the time required for symptom recognition, medical attention, and diagnostic testing.

The different patterns of association between serovars suggest that different rainfall-facilitated exposure pathways may dominate for each serovar. Hardjo was the predominant infecting serovar for farmers during the study period [7]. During wet weather, increased presence of wet surfaces and surface water on farms, combined with close contact with livestock, likely increases immediate exposure risk to Leptospira from actively shedding animals. This serovar is prevalent in sheep, beef cattle, and deer in New Zealand, with vaccination reportedly not widespread in dry-stock farming [37].

Some of the association of an increased risk of a case of human leptospirosis could also be due to rainfall facilitating exposure to *Leptospira* from environmental contamination rather than directly from a shedding animal. With rainfall, soil gains moisture which is likely to increase *Leptospira* survival in the environment for longer subsequent to the presence of shedding hosts [3, 35]. In addition, in heavy rain surface flooding may occur and pools of water can form that may: suspend *Leptospira* and facilitate contact with human hosts [4, 19]; or disperse *Leptospira* across the terrestrial environment and into waterways with increased risk of delayed animal and human exposure. The use of roof-collected rainwater has recently been identified as a risk factor for human leptospirosis in New Zealand [30]. Laboratory diagnosed ovine leptospirosis has also only recently been found to be associated with rainfall, and particularly extreme rainfall, in New Zealand, but the association was not significant for bovine leptospirosis [38]. All these recent findings, including the present study, reinforce the need for a multisectoral, research-informed One Health approach to leptospirosis prevention, surveillance, and control [39–41].

The association of total rainfall only at the 3-month lag with Pomona could suggest delayed effects compared to Hardjo or Ballum. This appears consistent with rainfall facilitating direct transmission between livestock around the time of rainfall, subsequently increasing prevalence of shedding animals and human exposure risk for occupations handling stock or carcasses [42]. In addition, in the recent New Zealand animal study [38] nearly 40% of the diagnosed ovine cases were positive to serovar Pomona, confirming the livestock link. In New Zealand, occupational exposure is still a prevalent transmission route [30], and these findings suggest rainfall may contribute to human exposure by exacerbating occupational risk. Therefore, while the present findings of association with rainfall is novel, the current public health messaging around prevention of occupational exposure involving livestock vaccination, use of personal protective equipment, awareness, and prompt medical attention remain valid and should be reinforced following extreme rainfall events.

The potential for differences in rainfall effects on different *Leptospira* strains is supported by Bulach et al. [43], who found genetic differences between *L. interrogans* and *L. borgpetersenii* relating to environmental survival, with higher viability retained by *L. interrogans* in water at 20 °C after 48 h. The variation between Hardjo and Pomona findings in terms of lag may relate to their different survival capabilities outside hosts. Hardjo (*L. borgpetersenii*) has more limited environmental survival than Pomona (*L. interrogans*), so rainfall may help facilitate timely host-to-host transmission.

Rodents are the main maintenance hosts for Ballum [10, 12], and the significant association at the 1-month lag could be consistent with exposure to environmental contamination from rodent urine or changes in rodent activity associated with heavy rainfall facilitating increased human exposure. Exposure to rodents was also only recently identified as a risk factor for human leptospirosis in New Zealand [30], confirming the emergence of novel exposure pathways where rainfall could play a role and the need to raise awareness in a country where leptospirosis has historically been known as ‘dairy farm fever’.”

### Study limitations

The use of disease occurrence at the DHB level risks ecological fallacy, though adequate sample sizes for greater geographical resolution would be limiting. The spatial resolution of climate stations was unlikely to ensure representative rainfall measurements for all DHBs or accurately reflect conditions where all cases occurred.

The robustness of the data constituting the variable animal density and the inclusion of a number of livestock species may have obscured an effect of animal species on leptospirosis occurrence. In addition, the model assumes that the different species posed the same risk of human leptospirosis when this is likely to vary, as animal species differ in their vaccination and infection prevalence and pose different exposure risks for humans. Schneider et al. [44] also found no association between animal density per km^2^ and the cumulative incidence rate of leptospirosis, but some studies have found animal density a factor in the occurrence of leptospirosis [45, 46]. If suitable data can be obtained, ideally at a monthly resolution to account for the seasonal farming system used in NZ, modelling individual species or known reservoir species combinations as co-variables in the models may be worth exploring to examine for any contribution of animal density.

A further consideration potentially relevant to the validity of the findings is that both people and animal hosts can move between DHBs through employment, recreation, or stock movements. ‘Gypsy Day’, an annual event on June 1st in New Zealand’s dairy farming calendar, results in large numbers of sharemilkers moving cows and families to new farms for the new milking season. Such movements may result in cases being attributed to a different DHB than where infection occurred or animal hosts infected in one DHB exposing people in another. This would result in incorrect rainfall levels being ascribed to cases in the models. However, such movements include individuals moving to DHBs with both higher and lower rainfall than their exposure location, and at the DHB geographical resolution, there was no reason to consider that these movements significantly bias the analysis towards or away from an association between rainfall and leptospirosis incidence.

This study only considered total monthly rainfall as a measure of the impact of rainfall on the number of reported cases of human leptospirosis. Studies overseas have found that the type of rainfall measurement used can affect findings when considering the association of rainfall and leptospirosis [47]. Further, this model did not adjust for all environmental, socioeconomic or occupational covariates potentially associated with the occurrence of leptospirosis [1, 47]. Future work could, in combination with rainfall, evaluate the impact on the occurrence of leptospirosis of temperature and other environmental variables (such as humidity, soil moisture, and topography), socioeconomic factors (urban vs. rural and socioeconomic deprivation), and further explore the relative importance of animal reservoirs [45].

## Conclusion

Our study identified a positive association between rainfall and the occurrence of human leptospirosis, confirming previous overseas reports. Notably, this study also found differences between serovars in the timing of the association between total rainfall and the reported occurrence of human leptospirosis, indicating that the exposure pathways facilitated or enhanced by rainfall may vary between serovars.

Further, when the timeline for the reporting of a disease occurrence in humans is considered, the results of the study also suggest that more than one exposure pathway may be facilitated by rainfall in New Zealand and that total rainfall may contribute to the occurrence of reported leptospirosis through pathways resulting in either infection (i) shortly after rainfall or (ii) delayed from the time of the rainfall.

These results have potential public health implications, and awareness should be maintained amongst the public, emergency response staff, and medical practitioners that the risk of exposure to *Leptospira* may be associated with increased levels of rainfall, and consideration should be given to precautionary actions during risk events to prevent human exposure to the urine of shedding animals and potentially contaminated environments. Considering prevention of leptospirosis more broadly, the finding of differences between the effect of rainfall on serovars suggests that the consideration of individual serovar risk conditions may be important in disease prevention and an area for further research.

## Supporting information

10.1017/S0950268825100423.sm001 Tana et al. supplementary material Tana et al. supplementary material

## Data Availability

Rainfall data used in this study are available in the Supplementary Material S1. The human case data set is unavailable due to privacy reasons and is the property of the New Zealand Institute of Environmental Science and Research.

## Supplementary material

The supplementary material for this article can be found at http://doi.org/10.1017/S0950268825100423.
