# Supplementary material for: The association between rainfall and human leptospirosis in Aotearoa New Zealand
Source: Epidemiol Infect. 2025 Aug 26;153:e112. doi: 10.1017/S0950268825100423 (PMC12529419; doi:10.1017/S0950268825100423)
Supplement: Tana et al. supplementary material [file S0950268825100423sup001.docx]

Epidemiology and Infection

The association between rainfall and human leptospirosis in Aotearoa New Zealand

Toni Tana, Masako Wada, Jackie Benschop, and Emilie Vallee

Supplementary material

Supplementary Material S1


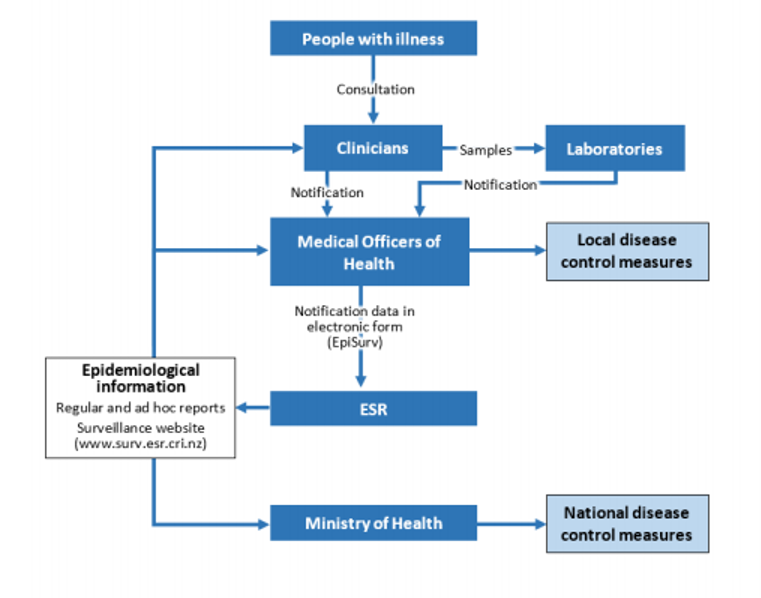


*Supplementary Figure S1: Flowchart of the New Zealand Ministry of Health’s Notifiable disease surveillance system in humans for the period 1999-2017 (ESR: the Institute of Environmental Science and Research.)*

New Zealand Ministry of Health Leptospirosis Case Report Form
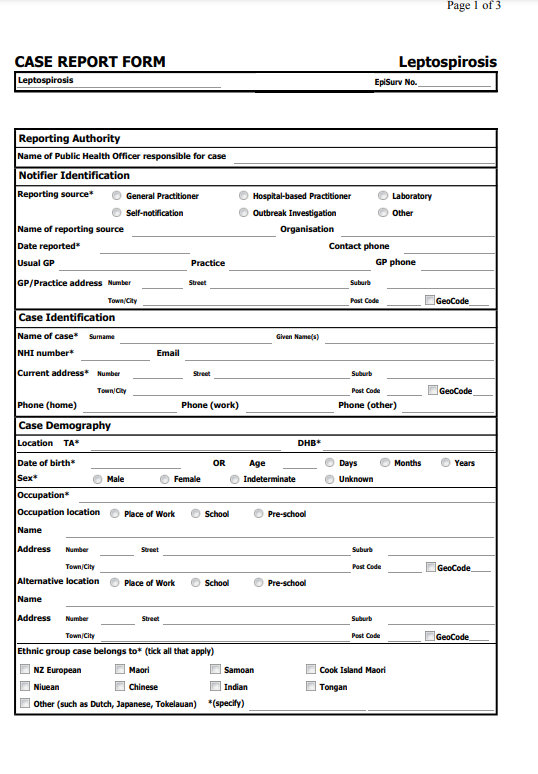

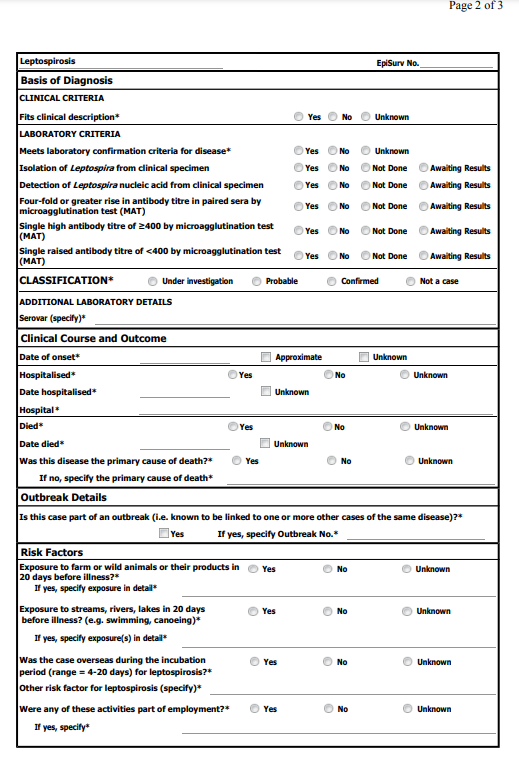

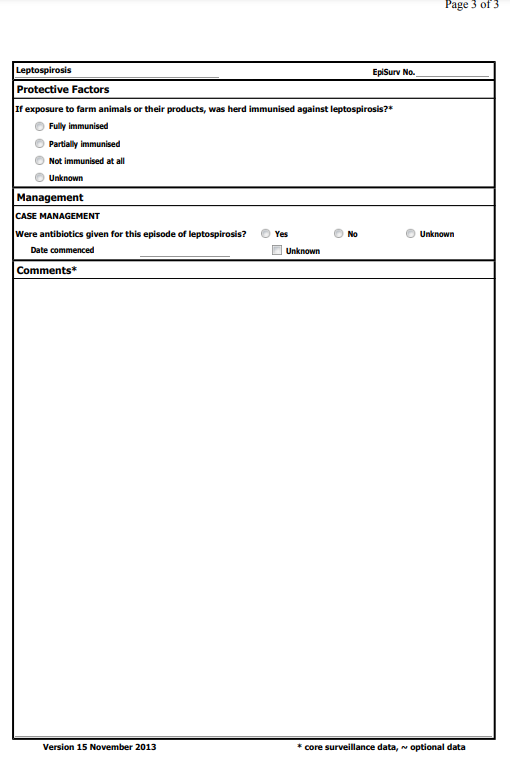


New Zealand Ministry of Health diagnostic definitions for leptospirosis

**Case definition^^[[1]](#footnote-1)^^**

**Clinical description**

An acute illness characterised by fever, chills, headache, myalgia, nausea, diarrhoea, abdominal pain, meningitis, cough and conjunctival suffusion. Manifestations of severe disease can include jaundice, renal failure, haemorrhage, pneumonitis and haemodynamic collapse.

**Laboratory test for diagnosis**

**Laboratory definitive evidence for a confirmed case requires**at least one of the following:

- isolation of leptospires from a clinical specimen
- detection of leptospiral nucleic acid from a clinical specimen
- a four-fold or greater rise in leptospiral microscopic agglutination titre (MAT) between acute and convalescent sera
- single high antibody titre of ≥ 400 in the MAT.

**Laboratory suggestive evidence for a probable case requires**single raised agglutination titre by MAT of < 400.

It is recommended that both nucleic acid testing (NAT) and MAT testing be undertaken to improve diagnostic accuracy. MAT is the current gold standard serological test and is used to identify the probable causative serovar/serogroup. ESR-NCBID is the national reference laboratory for MAT.

Serology

IgM can be detectable within the first week of illness and can persist for months. Seroconversion can take up to 3 weeks from the onset of symptoms. IgM is useful as a screening test but not a confirmatory test because of potential cross-reactivity with other diseases. For confirmatory testing acute and convalescent samples need to be tested in parallel by MAT. There should be a minimum of 2 weeks between collection of acute and convalescent sera.

Nucleic acid testing (for example, polymerase chain reaction – PCR)

NAT is highly sensitivity for diagnosis of leptospirosis. NAT can be used to detect leptospires in blood during the acute leptospiraemic phase of the disease typically before an antibody response is mounted. NAT can also be used to detect leptospires in urine during the second week of illness; shedding may be prolonged and intermittent. Leptospires can be excreted intermittently in the urine. Therefore, a negative result in the context of a compatible clinical illness cannot exclude the diagnosis of leptospirosis. In cases of high clinical suspicion, a second urine sample should be submitted if the initial specimen tested negative by NAT.

**Case classification**

- **Under investigation:** A case that has been notified, but information is not yet available to classify it as probable or confirmed.
- **Probable:** A clinically compatible illness with laboratory suggestive evidence.
- **Confirmed:** A clinically compatible illness with laboratory definitive evidence.
- **Not a case:** A case that has been investigated and subsequently found not to meet the case definition.

Supplementary Material S2: Additional Supplementary Tables and Figures

*Supplementary Table S1: Table summarising the descriptive information of the meteorological stations contributing rainfall data used in the study analysis*

| **Location Identifier** | **Station Name** | **Latitude** | **Longitude** | **Observation Start Date** | **Observation End Date** | **Percent complete*** | **District Health Board** |
| --- | --- | --- | --- | --- | --- | --- | --- |
| **1496** | Waiheke Island, Awaroa Valley | -36.807 | 175.1145 | 1-Mar-80 | 28-Feb-21 | 100 | Auckland |
| **1615** | Tauranga Aero Aws | -37.673 | 176.196 | 1-Jun-90 | 31-Mar-21 | 100 | Bay of Plenty |
| **1664** | Edgecumbe | -37.9725 | 176.8079 | 1-Jun-90 | 28-Feb-21 | 100 | Bay of Plenty |
| **1705** | Rukuhanga Stn | -37.566 | 178.0223 | 1-Aug-30 | 30-Nov-19 | 100 | Bay of Plenty |
| **1786** | Aniwhenua | -38.2953 | 176.7924 | 1-Jan-81 | 30-Nov-18 | 100 | Bay of Plenty |
| **4455** | Boyle River Lodge | -42.519 | 172.386 | 1-Nov-83 | 28-Feb-21 | 90 | Canterbury |
| **4497** | Kaikoura Plains | -42.383 | 173.656 | 1-Nov-80 | 31-Dec-20 | 100 | Canterbury |
| **4540** | Melrose Stn | -42.9207 | 172.4298 | 1-Oct-50 | 28-Feb-21 | 100 | Canterbury |
| **4560** | Hawkswood | -42.654 | 173.329 | 1-Jan-31 | 28-Feb-21 | 100 | Canterbury |
| **4644** | Glenthorne, Upper Station | -43.1797 | 171.444 | 1-Jan-25 | 31-Jan-21 | 100 | Canterbury |
| **4734** | Mt Somers | -43.706 | 171.401 | 1-Dec-80 | 28-Feb-21 | 100 | Canterbury |
| **4920** | Mcqueens Valley | -43.733 | 172.633 | 1-Jan-47 | 28-Feb-21 | 100 | Canterbury |
| **3373** | Judgeford | -41.123 | 174.941 | 1-Oct-78 | 28-Feb-21 | 100 | Capital and Coast |
| **3445** | Wellington Aero | -41.322 | 174.804 | 1-Jan-60 | 31-Mar-21 | 100 | Capital and Coast |
| **1962** | Auckland Aero | -37.0081 | 174.7887 | 1-Jan-62 | 31-Mar-21 | 100 | Counties Manukau |
| **2006** | Pukekohe Ews | -37.2064 | 174.8638 | 1-Mar-86 | 31-Mar-21 | 100 | Counties Manukau |
| **2523** | Flemington | -40.17 | 176.45 | 1-Dec-58 | 28-Feb-21 | 100 | Hawke's Bay |
| **2979** | Eskdale Hedgeley | -39.413 | 176.8138 | 01-Aug-1894 | 31-Jan-21 | 100 | Hawke's Bay |
| **3011** | Rose Hill | -39.648 | 176.557 | 1-Jan-38 | 28-Feb-21 | 100 | Hawke's Bay |
| **3142** | Mahia Aws | -39.1174 | 177.9618 | 1-Dec-90 | 31-Mar-21 | 90 | Hawke's Bay |
| **10709** | Te Kaihi | -40.055 | 176.783 | 1-Jan-95 | 28-Feb-21 | 100 | Hawke's Bay |
| **3476** | Trentham Racecourse | -41.14 | 175.041 | 1-Aug-30 | 28-Feb-21 | 100 | Hutt Valley |
| **3479** | U Hutt,Tennyson St | -41.124 | 175.043 | 1-Oct-54 | 31-Mar-19 | 100 | Hutt Valley |
| **1770** | Rotorua Aero Aws | -38.106 | 176.3149 | 1-May-82 | 31-Mar-21 | 80 | Lakes |
| **1857** | Rainbow Point | -38.7218 | 176.072 | 1-Sep-78 | 28-Feb-21 | 100 | Lakes |
| **2373** | Waipuna Woodville | -40.3308 | 175.8382 | 1-Nov-24 | 28-Feb-21 | 100 | MidCentral |
| **3253** | Bainesse | -40.4092 | 175.3785 | 1-Jan-74 | 28-Feb-21 | 100 | MidCentral |
| **3308** | Te Horo, Longcroft | -40.817 | 175.148 | 1-Mar-69 | 28-Feb-21 | 100 | MidCentral |
| **3832** | Cobb Power Station | -41.088 | 172.731 | 1-Feb-39 | 28-Feb-21 | 100 | Nelson Marlborough |
| **4255** | Linkwater | -41.291 | 173.872 | 1-Apr-34 | 28-Feb-21 | 100 | Nelson Marlborough |
| **4386** | Awatere Vly,Upcot | -41.881 | 173.582 | 1-Jan-32 | 28-Feb-21 | 100 | Nelson Marlborough |
| **24139** | Kaihoka | -40.573 | 172.571 | 1-Dec-83 | 28-Feb-21 | 100 | Nelson Marlborough |
| **1002** | Cape Reinga Aws | -34.4296 | 172.6819 | 1-Apr-84 | 31-Mar-21 | 60 | Northland |
| **1033** | Kaeo Northland | -35.057 | 173.74 | 1-Mar-81 | 28-Feb-21 | 100 | Northland |
| **1189** | Mamaranui | -35.864 | 173.8 | 1-Jan-51 | 28-Feb-21 | 100 | Northland |
| **1287** | Whangarei Aero Aws | -35.769 | 174.364 | 1-Aug-90 | 31-Mar-21 | 100 | Northland |
| **4771** | Orari Gorge | -43.976 | 171.196 | 01-Jan-1899 | 30-Jun-20 | 100 | South Canterbury |
| **4968** | L Pukaki, Guide Hill | -44.004 | 170.247 | 1-Oct-63 | 28-Feb-21 | 100 | South Canterbury |
| **5100** | Moanaroa | -44.603 | 171.095 | 1-Jan-64 | 31-Jan-21 | 100 | South Canterbury |
| **4107** | Milford Sound | -44.6738 | 167.9241 | 1-Dec-29 | 30-Sep-19 | 100 | Southern |
| **4141** | Puysegur Point Aws | -46.156 | 166.6107 | 1-Jan-91 | 28-Feb-21 | 90 | Southern |
| **5110** | Lake Ohau Stn | -44.168 | 169.8222 | 1-May-48 | 31-Dec-20 | 100 | Southern |
| **5142** | Oamaru Airport Aws | -44.9711 | 171.0824 | 1-May-82 | 31-Mar-21 | 70 | Southern |
| **5183** | Makarora Station | -44.251 | 169.224 | 1-Aug-24 | 28-Feb-21 | 100 | Southern |
| **5323** | Palmerston | -45.4755 | 170.7144 | 1-Mar-69 | 28-Feb-21 | 100 | Southern |
| **5335** | Lee Flat | -45.783 | 170.027 | 1-Mar-54 | 28-Feb-21 | 100 | Southern |
| **5430** | Manapouri Aero Aws | -45.5328 | 167.6417 | 1-Feb-91 | 31-Mar-21 | 90 | Southern |
| **5446** | Queenstown | -45.0348 | 168.6636 | 01-Sep-1871 | 28-Feb-21 | 90 | Southern |
| **5496** | Lumsden Aws | -45.748 | 168.449 | 1-May-82 | 31-Mar-21 | 70 | Southern |
| **5537** | Lauder Flat | -45.008 | 169.703 | 1-Jan-45 | 31-Jan-21 | 100 | Southern |
| **5741** | Orepuki 2 | -46.287 | 167.736 | 1-Sep-63 | 28-Feb-21 | 100 | Southern |
| **5823** | Tiwai Point Ews | -46.587 | 168.376 | 1-May-70 | 31-Mar-21 | 100 | Southern |
| **5893** | Nugget Point Aws | -46.4469 | 169.8112 | 1-Aug-83 | 31-Mar-21 | 70 | Southern |
| **1905** | Motu Ews | -38.2857 | 177.5294 | 1-Nov-90 | 31-Mar-21 | 90 | Tairawhiti |
| **2692** | Hicks Bay Aws | -37.562 | 178.3142 | 1-Dec-90 | 31-Mar-21 | 90 | Tairawhiti |
| **2897** | Mokairau, Pakarae | -38.4754 | 178.242 | 1-Jan-47 | 28-Feb-21 | 100 | Tairawhiti |
| **2311** | Tarata | -39.1613 | 174.3649 | 1-Jun-51 | 28-Feb-21 | 100 | Taranaki |
| **3505** | Patiki | -39.4626 | 173.9748 | 1-Jan-63 | 28-Feb-21 | 100 | Taranaki |
| **1520** | Whitianga Aero Aws | -36.834 | 175.677 | 1-Sep-90 | 31-Mar-21 | 90 | Waikato |
| **1563** | Waitoa | -37.5857 | 175.5983 | 1-Jul-87 | 29-Feb-20 | 90 | Waikato |
| **2107** | Ngahinapouri | -37.8895 | 175.2322 | 1-Jan-35 | 30-Jun-20 | 100 | Waikato |
| **2220** | Mangakowhai | -38.396 | 175.029 | 1-Mar-95 | 28-Feb-21 | 100 | Waikato |
| **2264** | Owhango | -38.986 | 175.377 | 1-May-67 | 28-Feb-21 | 100 | Waikato |
| **12558** | Lichfield, Scriveners Road | -38.0914 | 175.8234 | 1-Jan-96 | 28-Feb-21 | 100 | Waikato |
| **2589** | Waiawa, Whareama | -40.9305 | 176.0511 | 1-May-68 | 28-Feb-21 | 100 | Wairarapa |
| **2624** | Kahutara | -41.194 | 175.348 | 1-Oct-81 | 31-Aug-20 | 100 | Wairarapa |
| **1400** | Whangaparaoa Aws | -36.606 | 174.835 | 1-Mar-87 | 28-Feb-21 | 70 | Waitemata |
| **1423** | Auckland, Henderson North | -36.8554 | 174.6238 | 1-Nov-85 | 28-Feb-21 | 100 | Waitemata |
| **3925** | Reefton Ews | -42.1158 | 171.8601 | 1-Aug-60 | 31-Mar-21 | 100 | West Coast |
| **3992** | Kowhitirangi No 2 | -42.885 | 171.032 | 1-Feb-65 | 31-Aug-19 | 100 | West Coast |
| **4054** | Lower Whataroa | -43.199 | 170.373 | 1-Mar-49 | 28-Feb-21 | 100 | West Coast |
| **4097** | Haast Aws | -43.8603 | 169.0063 | 1-May-82 | 31-Mar-21 | 60 | West Coast |
| **3596** | Raetihi, Miranui | -39.3771 | 175.2668 | 1-Jan-79 | 31-Jan-21 | 100 | Whanganui |
| **3652** | Moawhango, Homestead | -39.5803 | 175.8689 | 1-Jan-70 | 31-Oct-20 | 100 | Whanganui |
| **3715** | Whanganui Spriggens Park Ews | -39.937 | 175.0451 | 01-Jan-1890 | 31-Mar-21 | 100 | Whanganui |
| **3737** | Ngahere Iti | -39.9956 | 175.5365 | 1-May-61 | 28-Feb-21 | 100 | Whanganui |

**Percentage of monthly rainfall measurements recorded by a station. The denominator being equal to if a measurement was taken every month of the station’s operation over the period of the study (n=228 months).*


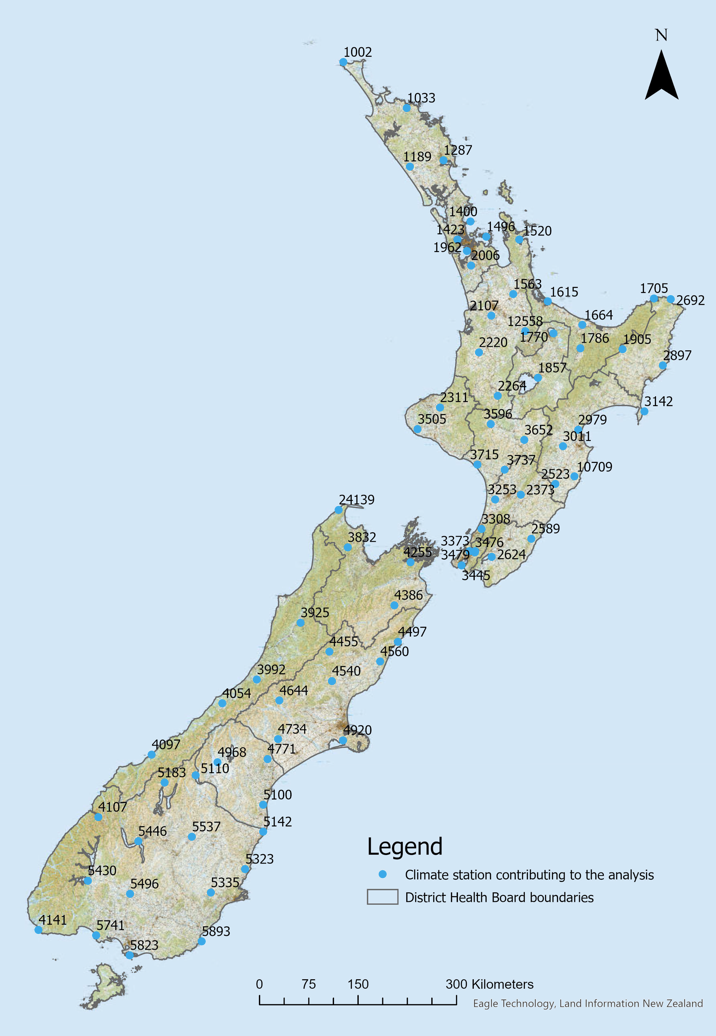


*Supplementary Figure S2: A map of New Zealand showing the location and identification number of the NIWA climate stations used in this study in relation to the District Health Board boundaries.Map lines delineate study areas and do not necessarily depict accepted national boundaries.*

*Supplementary Table S2: New Zealand human population data for the years 1999 to 2017 utilised in analysis (StatsNZ, 2021c)*

| District health board | | Year | | | | | | | | | | | | | | | | | |
| --- | --- | --- | --- | --- | --- | --- | --- | --- | --- | --- | --- | --- | --- | --- | --- | --- | --- | --- | --- |
|  | **1999** | **2000** | **2001** | **2002** | **2003** | **2004** | **2005** | **2006** | **2007** | **2008** | **2009** | **2010** | **2011** | **2012** | **2013** | **2014** | **2015** | **2016** | **2017** |
| Northland | 143800 | 144400 | 144400 | 146000 | 147900 | 149400 | 151000 | 152700 | 154600 | 156300 | 158200 | 160600 | 162500 | 163500 | 164700 | 168200 | 172100 | 176400 | 181200 |
| Waitemata | 435700 | 442700 | 449000 | 461400 | 476000 | 487100 | 495700 | 504700 | 511700 | 517400 | 523600 | 530700 | 538500 | 545400 | 552800 | 563000 | 575100 | 588500 | 602900 |
| Auckland | 381100 | 384300 | 388800 | 401100 | 414200 | 419300 | 423500 | 428300 | 432200 | 436000 | 441000 | 445900 | 451700 | 456000 | 460500 | 466200 | 472800 | 481400 | 488000 |
| Counties Manukau | 380900 | 387500 | 393700 | 406900 | 421300 | 433800 | 444200 | 454800 | 461600 | 467400 | 472900 | 478900 | 485600 | 491500 | 496300 | 508000 | 522500 | 538300 | 553800 |
| Waikato | 327900 | 328600 | 328600 | 332900 | 338000 | 342700 | 346300 | 350200 | 353800 | 357500 | 362000 | 366800 | 371300 | 374800 | 377900 | 384700 | 393000 | 402000 | 412200 |
| Lakes | 99400 | 99500 | 99400 | 100100 | 100900 | 101300 | 101300 | 101500 | 101400 | 101500 | 101800 | 102600 | 103100 | 103300 | 103200 | 104900 | 106800 | 109100 | 111400 |
| Bay of Plenty | 178800 | 181600 | 183400 | 186700 | 190600 | 194400 | 197700 | 200800 | 203600 | 205900 | 208400 | 211000 | 213200 | 213900 | 215000 | 220100 | 226100 | 232900 | 241600 |
| Tairawhiti | 46400 | 46100 | 45500 | 45500 | 45800 | 45800 | 45800 | 45900 | 45900 | 46000 | 46300 | 46700 | 46800 | 47000 | 47000 | 47500 | 48100 | 48800 | 49200 |
| Taranaki | 107500 | 106700 | 105900 | 106100 | 106700 | 107000 | 107000 | 107400 | 107700 | 108400 | 109500 | 110800 | 112000 | 112900 | 113800 | 115100 | 116800 | 118600 | 120100 |
| Hawke's Bay | 147200 | 147200 | 147200 | 148400 | 149200 | 150300 | 151000 | 151900 | 152800 | 153400 | 154400 | 156200 | 157200 | 157400 | 157900 | 160700 | 163600 | 166800 | 169600 |
| Whanganui | 67500 | 66700 | 65600 | 65200 | 65000 | 64600 | 64200 | 64000 | 63500 | 63200 | 63100 | 63100 | 63000 | 62400 | 62300 | 63100 | 64000 | 65100 | 65900 |
| MidCentral | 161500 | 161200 | 160300 | 161100 | 162200 | 163000 | 163300 | 164000 | 164100 | 164500 | 165500 | 166600 | 167800 | 168500 | 168900 | 171200 | 173600 | 176400 | 179000 |
| Hutt Valley | 137100 | 137000 | 136700 | 137800 | 138700 | 139800 | 140400 | 140900 | 141200 | 141100 | 141700 | 142400 | 142900 | 142500 | 142500 | 144400 | 146700 | 149600 | 152100 |
| Capital and Coast | 251700 | 253400 | 256300 | 260600 | 266200 | 270700 | 273800 | 278000 | 280500 | 282500 | 285300 | 287700 | 290400 | 292200 | 293500 | 297700 | 302500 | 307400 | 311500 |
| Wairarapa | 39000 | 39000 | 39200 | 39300 | 39400 | 39400 | 39500 | 39600 | 39800 | 40200 | 40600 | 41200 | 41800 | 42100 | 42400 | 43200 | 44000 | 44800 | 45700 |
| Nelson Marlborough | 123400 | 124600 | 126000 | 127500 | 129600 | 131500 | 132600 | 133600 | 134600 | 135800 | 137000 | 138500 | 140500 | 141300 | 142200 | 144800 | 147400 | 150400 | 152900 |
| West Coast | 31900 | 31400 | 31000 | 31100 | 31400 | 31500 | 31800 | 32100 | 32300 | 32400 | 32700 | 32800 | 33100 | 33100 | 33000 | 32900 | 32800 | 32900 | 32700 |
| Canterbury | 435700 | 438700 | 442000 | 450100 | 460200 | 469100 | 476100 | 484000 | 491100 | 497400 | 503900 | 510500 | 501500 | 497800 | 504300 | 511300 | 525700 | 541000 | 552700 |
| South Canterbury | 54500 | 54000 | 53800 | 54100 | 54400 | 54800 | 54900 | 55100 | 55300 | 55500 | 55800 | 56200 | 56700 | 57100 | 57600 | 58100 | 58800 | 59800 | 60500 |
| Southern | 283600 | 282500 | 283200 | 286100 | 289100 | 291600 | 293100 | 294600 | 295700 | 297000 | 298700 | 301000 | 303900 | 304900 | 306400 | 311100 | 316900 | 323800 | 330400 |
| Total | 3834600 | 3857100 | 3880000 | 3948000 | 4026800 | 4087100 | 4133200 | 4184100 | 4223400 | 4259400 | 4302400 | 4350200 | 4383500 | 4407600 | 4442200 | 4516200 | 4609300 | 4714000 | 4813400 |

*Supplementary Table S3: Crude animal population data obtained from the 2002, 2007, 2012, and 2017 New Zealand Agricultural Production Surveys.*

| **Territorial Authority** | **Species and year** | | | | | | | | | | | | | | | |  |
| --- | --- | --- | --- | --- | --- | --- | --- | --- | --- | --- | --- | --- | --- | --- | --- | --- | --- |
|  | **Dairy Cattle** | | | | **Beef Cattle** | | | | **Sheep** | | | | **Deer** | | | |  |
|  | **2002** | **2007** | **2012** | **2017** | **2002** | **2007** | **2012** | **2017** | **2002** | **2007** | **2012** | **2017** | **2002** | **2007** | **2012** | **2017** |  |
| **Far North District** | 109405 | 90800 | 104753 | 114875 | 230054 | 229655 | 184818 | 177509 | 219695 | 233164 | 198892 | 145262 | 6052 | 2880 | 1899 | 2178 |  |
| **Whangarei District** | 149141 | 127849 | 135694 | 128540 | 107593 | 113360 | 84426 | 84079 | 135078 | 97057 | 84024 | 62933 | 7332 | 2419 | 2237 | 2570 |  |
| **Kaipara District** | 146841 | 148534 | 157317 | 135986 | 130785 | 152818 | 111475 | 121369 | 167416 | 204231 | 158039 | 119838 | 10028 | 2267 | 1022 | 165 |  |
| **Rodney District** | 74293 | 59323 | WD | WD | 97118 | 96739 | WD | WD | 207845 | 181579 | WD | WD | 13996 | 9624 | WD | WD |  |
| **North Shore City** | 207 |  | WD | WD | 0 |  | WD | WD |  |  | WD | WD |  | 0 | WD | WD |  |
| **Waitakere City** |  |  | WD | WD | 2906 | 3738 | WD | WD | 3698 |  | WD | WD |  | 0 | WD | WD |  |
| **Auckland City** | 1966 |  | 117281 | 132323 | 7609 | 3791 | 117458 | 11948 | 41664 | 33297 | 205270 | 253074 |  | 0 | 12647 | 11284 |  |
| **Manukau City** | 11019 | 5429 | WD | WD | 18366 | 13160 | WD | WD | 41485 | 39237 | WD | WD | 810 |  | WD | WD |  |
| **Papakura District** | 5549 | 2496 | WD | WD | 5754 |  | WD | WD | 7611 |  | WD | WD | 756 |  | WD | WD |  |
| **Franklin District** | 113471 | 104040 | WD | WD | 105371 | 102913 | WD | WD | 265565 | 240226 | WD | WD | 10459 | 7847 | WD | WD |  |
| **Thames-Coromandel District** | 40251 | 32434 | 33164 | 30742 | 35700 | 33435 | 25657 | 22659 | 93417 | 82707 | 46016 | 25967 | 1793 | 596 | 311 | 238 |  |
| **Hauraki District** | 144818 | 148240 | 157012 | 149885 | 21933 | 24101 | 20229 | 19154 | 42232 | 52738 | 21644 | 26456 | 1856 | 726 | 0 | 872 |  |
| **Waikato District** | 241198 | 251977 | 325305 | 300650 | 142696 | 147380 | 170097 | 167231 | 402791 | 381494 | 425126 | 353693 | 20269 | 5736 | 7509 | 5143 |  |
| **Matamata-Piako District** | 377804 | 349144 | 365415 | 370626 | 31997 | 41310 | 24326 | 27133 | 50708 | 71387 | 35213 | 23774 | 9548 | 4016 | 2096 | 1450 |  |
| **Hamilton City** | 4770 | 8103 | 6787 |  | 3715 | 2225 | 1551 | 1865 | 664 |  |  | 1261 | 510 | 0 |  | 41 |  |
| **Waipa District** | 266687 | 245858 | 264666 | 269628 | 50237 | 50517 | 37019 | 37190 | 131665 | 116641 | 78432 | 66282 | 18634 | 15594 | 14573 | 9260 |  |
| **Otorohanga District** | 157144 | 155418 | 186213 | 172158 | 69678 | 66930 | 49937 | 47204 | 291436 | 285614 | 183380 | 146331 | 9077 | 8208 | 8732 | 4209 |  |
| **South Waikato District** | 144054 | 157840 | 175286 | 196731 | 12176 | 14362 | 10905 | 11814 | 39010 | 50771 | 29455 | 34083 | 7829 | 5198 | 4095 | 2520 |  |
| **Waitomo District** | 53195 | 61031 | 87040 | 93312 | 152935 | 143707 | 116305 | 106394 | 880121 | 932135 | 690723 | 602605 | 13612 | 12646 | 5942 | 4010 |  |
| **Taupo District** | 80364 | 99537 | 125796 | 176566 | 71674 | 76449 | 43751 | 46815 | 442107 | 487687 | 285641 | 240877 | 53838 | 60465 | 41712 | 37860 |  |
| **Western Bay of Plenty District** | 106410 | 99682 | 97557 | 99004 | 44289 | 44988 | 33369 | 28842 | 112115 | 90373 | 75435 | 49326 | 21928 | 13305 | 9505 | 5856 |  |
| **Tauranga City** | 3325 | 2076 |  |  | 4764 |  | 1472 | 2197 | 3753 |  |  |  | 1012 |  |  |  |  |
| **Rotorua District** | 165002 | 161677 | 170432 | 175420 | 45792 | 40048 | 34721 | 37698 | 220848 | 217842 | 168632 | 137689 | 38612 | 34086 | 25381 | 21038 |  |
| **Whakatane District** | 124301 | 117164 | 117046 | 116221 | 33928 | 27222 | 19942 | 29637 | 66014 | 42810 | 45488 | 46365 | 8723 | 3636 | 2095 | 2017 |  |
| **Kawerau District** |  | 0 |  | 1840 |  |  |  | 88 |  |  |  | 0 |  | 0 |  | 0 |  |
| **Opotiki District** | 36114 | 29060 | 30587 | 28847 | 15225 | 15376 | 11906 | 12045 | 36216 | 35648 | 20898 | 39500 | 2810 |  |  | 764 |  |
| **Gisborne District** | 12533 | 7891 | 17095 | 9407 | 309823 | 287296 | 267599 | 247238 | 1679377 | 1825496 | 1547294 | 1412045 | 25752 | 26694 | 17323 | 11859 |  |
| **Wairoa District** | 8050 |  | 5162 | 4765 | 120340 | 113076 | 104099 | 92005 | 593071 | 766108 | 552832 | 513155 | 18215 |  | 7797 | 7327 |  |
| **Hastings District** | 33312 | 31823 | 41244 | 28315 | 222355 | 155596 | 186461 | 173989 | 1517829 | 1246094 | 1240228 | 1095359 | 48920 | 34702 | 30580 | 18840 |  |
| **Napier City** | 913 |  | 298 |  | 4399 | 3869 | 3458 | 3514 | 26042 | 22960 | 29330 | 25249 | 1151 |  |  | 7 |  |
| **Central Hawke's Bay District** | 38794 | 33536 | 46063 | 51605 | 202990 | 157794 | 168431 | 142009 | 1588831 | 1495088 | 1369734 | 1086456 | 57688 | 38373 | 30954 | 24074 |  |
| **New Plymouth District** | 182546 | 147315 | 155903 | 151606 | 43164 | 47069 | 36486 | 40429 | 185700 | 174925 | 131145 | 133704 | 4068 | 2655 | 1312 | 1096 |  |
| **Stratford District** | 96372 | 82154 | 83729 | 73738 | 36425 | 40876 | 33763 | 40387 | 238736 | 238626 | 178449 | 210236 | 2090 |  | 1357 | 710 |  |
| **South Taranaki District** | 373213 | 360490 | 365807 | 365556 | 55030 | 55205 | 42970 | 44665 | 329118 | 299266 | 182727 | 207322 | 4604 |  | 1087 | 2193 |  |
| **Ruapehu District** | 16675 | 17334 | 27916 | 27598 | 156525 | 151300 | 131504 | 124797 | 1311108 | 1411494 | 1179726 | 1062856 | 36151 | 26397 | 16103 | 11508 |  |
| **Wanganui District** | 27421 | 23665 | 25233 | 24608 | 79106 | 65117 | 60283 | 58455 | 682454 | 676692 | 579661 | 569923 | 14684 | 8824 | 2845 | 5182 |  |
| **Rangitikei District** | 39980 | 42258 | 56995 | 51829 | 156060 | 162866 | 140256 | 138825 | 1708035 | 1774007 | 1536101 | 1283010 | 25298 | 21976 | 20034 | 16896 |  |
| **Manawatu District** | 109437 | 110582 | 139038 | 120470 | 113372 | 115109 | 87366 | 87970 | 900178 | 912607 | 662939 | 639578 | 41281 | 29159 | 20251 | 13159 |  |
| **Palmerston North City** | 24384 | 18431 | 20267 | 31469 | 9509 | 7932 | 7069 | 11574 | 80259 | 69690 | 61537 | 52155 | 4822 |  | 2107 | 1088 |  |
| **Tararua District** | 125565 | 113775 | 135575 | 124317 | 177697 | 149505 | 131547 | 123586 | 1805483 | 1830414 | 1531286 | 1358369 | 18981 | 14022 | 10686 | 6242 |  |
| **Horowhenua District** | 72787 | 67062 | 69386 | 82412 | 30080 | 28895 | 18208 | 19617 | 74514 | 91740 | 59788 | 75883 | 6655 | 3058 | 2653 | 2355 |  |
| **Kapiti Coast District** | 12023 | 8913 | 8506 | 6405 | 5646 | 8635 | 6163 | 3792 | 22471 | 43803 | 22126 | 8175 | 4572 | 4595 | 3428 | 3902 |  |
| **Porirua City** |  |  |  |  | 2245 | 2402 | 2841 | 1867 | 29303 | 36914 | 34385 | 21496 | 1464 |  | 1630 | 531 |  |
| **Upper Hutt City** | 2099 | 1442 | 2430 | 1037 | 2438 | 2714 | 1944 | 3337 | 6720 | 11473 | 11565 |  | 486 |  |  | 0 |  |
| **Lower Hutt City** | 1723 | 0 |  | 537 | 1322 | 517 | 288 | 426 | 21763 | 6916 | 6499 |  |  |  |  | 0 |  |
| **Wellington City** |  |  |  |  | 3070 | 1734 | 2800 | 2488 | 29084 | 19719 | 16274 | 14605 | 874 |  |  | 136 |  |
| **Masterton District** | 16186 | 15044 | 22945 | 16449 | 79965 | 63866 | 57885 | 56759 | 958301 | 953463 | 844506 | 822747 | 8234 | 3703 | 3698 | 1931 |  |
| **Carterton District** | 32154 | 25609 | 27363 | 21624 | 27673 | 23395 | 23509 | 20255 | 271670 | 279285 | 296794 | 233623 | 6953 | 4467 | 3849 | 3782 |  |
| **South Wairarapa District** | 45805 | 41656 | 46549 | 49796 | 58806 | 52384 | 44796 | 45969 | 471684 | 467472 | 430543 | 370554 | 4752 | 1328 | 734 |  |  |
| **Tasman District** | 67473 | 63849 | 71956 | 66114 | 49032 | 51428 | 40149 | 39805 | 356240 | 348485 | 276877 | 261878 | 32185 | 20632 | 15540 | 10348 |  |
| **Nelson City** |  | 1862 |  | 2925 | 2642 | 1298 |  |  | 11089 | 7639 | 6466 |  | 2228 |  |  | 0 |  |
| **Marlborough District** | 32526 | 23899 | 33218 | 25315 | 72479 | 65768 | 60127 | 52448 | 785115 | 578805 | 547180 | 468697 | 22842 |  |  | 6077 |  |
| **Kaikoura District** | 13862 | 11487 | 13361 | 12599 | 17000 | 18625 | 12169 | 15143 | 169817 | 143215 | 85308 | 85468 | 7398 | 9102 | 7740 | 3059 |  |
| **Buller District** | 48891 | 55652 | 60333 | 51122 | 10474 | 7724 | 5759 | 6807 | 30029 | 16643 | 10934 | 14348 | 12710 |  |  | 10213 |  |
| **Grey District** | 27524 | 40676 | 45741 | 45743 | 8999 | 7388 | 7414 | 8090 | 40559 | 19419 | 20531 | 17200 | 12494 | 15558 | 15172 | 15568 |  |
| **Westland District** | 48225 | 56153 | 67577 | 59339 | 19435 | 15163 | 15829 | 12525 | 21920 | 18032 |  | 8836 | 8058 |  |  | 2559 |  |
| **Hurunui District** | 45271 | 53099 | 103121 | 121836 | 117623 | 119141 | 97864 | 91833 | 1643785 | 1612116 | 1276364 | 1082804 | 42988 | 34042 | 26015 | 28364 |  |
| **Waimakariri District** | 47820 | 67671 | 112441 | 95194 | 41547 | 47755 | 40335 | 32172 | 432470 | 402750 | 282165 | 202252 | 42682 | 42269 | 24852 | 16895 |  |
| **Christchurch City** | 11354 | 15706 | 11192 | 51273 | 33160 | 36932 | 30452 | 36307 | 330118 | 313340 | 256178 | 253331 | 8644 | 13349 | 2403 | 5802 |  |
| **Selwyn District** | 105069 | 148181 | 199014 | 229028 | 53838 | 65912 | 52384 | 51343 | 1049428 | 954373 | 745487 | 548069 | 45420 | 48716 | 40759 | 19979 |  |
| **Ashburton District** | 188019 | 261134 | 430174 | 451567 | 96772 | 114382 | 78706 | 79957 | 1475764 | 1293527 | 880789 | 748205 | 96661 | 68390 | 49246 | 41534 |  |
| **Timaru District** | 69920 | 100053 | 142727 | 153569 | 51282 | 70110 | 51878 | 55561 | 766105 | 697432 | 472278 | 396625 | 80356 | 86873 | 64949 | 59243 |  |
| **Mackenzie District** | 5168 | 9048 | 23916 | 29411 | 36255 | 39551 | 42387 | 35506 | 667407 | 626549 | 521238 | 390298 | 36506 | 43183 | 40986 | 34082 |  |
| **Waimate District** | 47190 | 72840 | 132961 | 126819 | 42772 | 55940 | 47952 | 47334 | 776167 | 726238 | 518714 | 445495 | 42671 | 39003 | 27613 | 22419 |  |
| **Waitaki District** | 64517 | 71095 | 118069 | 118733 | 57926 | 74768 | 66215 | 69944 | 1080656 | 978234 | 808829 | 755936 | 40052 | 41253 | 23403 | 15650 |  |
| **Central Otago District** | 13223 | 11939 | 48384 | 45208 | 61115 | 66183 | 80236 | 66999 | 1720002 | 1756092 | 1543135 | 1323549 | 55613 | 45504 | 39537 | 25010 |  |
| **Queenstown-Lakes District** | 3955 | 3184 | 2610 | 3985 | 23193 | 23720 | 23167 | 20757 | 403171 | 373093 | 352392 | 301462 | 28932 | 45800 | 44547 | 37778 |  |
| **Dunedin City** | 35136 | 36102 | 35327 | 42699 | 46851 | 47976 | 52350 | 41151 | 803546 | 810618 | 812296 | 658838 | 26961 | 19056 | 17076 | 13151 |  |
| **Clutha District** | 96908 | 111662 | 163274 | 159987 | 93218 | 96166 | 85049 | 86366 | 2560818 | 2510411 | 2135683 | 1868365 | 53400 | 46396 | 35881 | 31397 |  |
| **Southland District** | 296817 | 374218 | 580079 | 575631 | 173062 | 175061 | 145619 | 145166 | 4877430 | 4619229 | 3488393 | 3133071 | 312410 | 277839 | 211202 | 174321 |  |
| **Gore District** | 45375 | 42802 | 73407 | 81437 | 26431 | 26843 | 21291 | 24448 | 978975 | 962385 | 792004 | 739442 | 30685 | 23822 | 21492 | 12875 |  |
| **Invercargill City** | 14028 | 15622 | 17095 | 23943 | 4177 | 5684 | 5240 | 5587 | 94252 | 80773 | 76030 | 114781 | 8463 | 5863 | 5708 | 5284 |  |
|  | **Pigs** | | | | **Goats** | | | | **Horse** | | | | **Alpaca** | | | |  |
|  | **2002** | **2007** | **2012** | **2017** | **2002** | **2007** | **2012** | **2017** | **2002** | **2007** | **2012** | **2017** | **2002** | **2007** | **2012** | **2017** | **Land Area^^[[2]](#footnote-2)^^**  **(km^2^)** |
| **Far North District** | 1028 | 562 | 574 | 232 | 5369 | 1990 | 875 | 126 | 1374 | 1197 | 1292 | 689 |  | 122 | 374 | 121 | 6677.693 |
| **Whangarei District** | 484 |  |  | 85 | 3364 | 3077 | 3408 | 3094 | 902 | 875 | 1014 | 456 |  |  | 444 | 170 | 2711.516 |
| **Kaipara District** | 3196 |  |  | 2736 | 2358 | 1093 | 770 | 419 | 821 | 652 | 720 | 555 |  |  | 52 | 4 | 3108.724 |
| **Rodney District** |  |  | WD | WD | 6654 | 2794 | WD | WD | 2128 | 2228 | WD | WD |  | 662 | WD | WD | 2372.484 |
| **North Shore City** |  | 0 | WD | WD |  |  | WD | WD |  | 0 | WD | WD |  | 0 | WD | WD | 127.8536 |
| **Waitakere City** |  |  | WD | WD |  |  | WD | WD | 213 | 105 | WD | WD |  |  | WD | WD | 367.1399 |
| **Auckland City** | 46 |  |  | 10102 |  |  | 1539 | 2259 |  |  | 5800 | 3439 |  |  | 1539 | 935 | 629.2666 |
| **Manukau City** | 94 | 46 | WD | WD | 1281 | 560 | WD | WD | 1657 | 1022 | WD | WD |  |  | WD | WD | 543.5892 |
| **Papakura District** |  |  | WD | WD | 52 |  | WD | WD | 849 | 598 | WD | WD |  |  | WD | WD | 118.589 |
| **Franklin District** | 17041 | 12686 | WD | WD | 3713 | 3044 | WD | WD | 4504 | 3848 | WD | WD |  | 278 | WD | WD | 2176.796 |
| **Thames-Coromandel District** |  |  |  | 984 | 1079 | 292 | 168 | 39 | 432 | 318 | 241 | 143 |  |  | 130 | 29 | 2206.919 |
| **Hauraki District** |  |  | 122 | 12 | 1399 | 504 | 64 |  | 448 | 440 | 268 | 185 |  | 31 | 122 | 57 | 1188.614 |
| **Waikato District** | 14590 | 15163 | 16510 | 13160 | 3395 | 7245 | 14921 | 16111 | 2704 | 3462 | 3944 | 4214 |  | 164 | 457 | 597 | 3088.139 |
| **Matamata-Piako District** | 7344 | 4497 |  | 347 | 7064 | 10724 | 13996 | 23044 | 3727 | 3543 | 3539 | 3787 |  |  | 135 |  | 1755.456 |
| **Hamilton City** | 9 |  |  | 93 |  |  |  | 105 | 351 | 486 | 192 | 305 |  | 0 |  | 0 | 98.03247 |
| **Waipa District** | 15192 | 4384 |  | 3662 | 8715 | 7137 | 5352 | 8782 | 5004 | 5218 | 4154 | 5122 |  |  | 342 | 498 | 1470.175 |
| **Otorohanga District** |  |  |  | 11466 | 4572 |  | 955 | 1823 | 729 | 375 | 294 | 284 |  |  | 100 | 0 | 1999.356 |
| **South Waikato District** | 177 | 184 | 95 | 39 | 1397 | 2341 | 3227 | 2454 | 533 | 323 | 315 | 192 |  |  | 9 |  | 1818.998 |
| **Waitomo District** | 248 | 252 | 304 | 121 | 1954 | 916 | 500 | 778 | 758 | 463 | 449 | 224 |  |  |  | 75 | 3534.841 |
| **Taupo District** | 248 | 263 | 358 | 71 | 1073 |  |  |  | 519 | 464 | 447 | 223 |  | 72 | 30 | 6 | 6333.613 |
| **Western Bay of Plenty District** | 4372 | 1990 | 513 | 1334 | 10646 | 3992 | 2598 | 968 | 1498 | 1010 | 821 | 453 |  | 338 | 652 | 214 | 1956.903 |
| **Tauranga City** |  |  | 6 |  | 580 |  |  |  | 216 |  |  |  |  | 0 |  | 2 | 128.4558 |
| **Rotorua District** | 436 | 250 |  | 104 | 1615 | 500 | 231 | 169 | 620 | 610 | 389 | 418 |  | 197 | 273 | 527 | 2409.291 |
| **Whakatane District** |  |  |  | 175 | 1490 | 1186 | 2591 | 706 | 556 | 493 | 436 | 360 |  |  |  | 27 | 4451.889 |
| **Kawerau District** |  | 0 |  | 0 |  | 0 |  | 0 |  |  |  |  |  | 0 |  | 0 | 21.98955 |
| **Opotiki District** | 280 | 138 | 82 | 30 | 949 |  | 513 | 69 | 198 | 220 | 131 | 101 |  |  |  | 0 | 3088.957 |
| **Gisborne District** | 1453 | 1857 |  | 159 | 4536 | 817 | 394 | 763 | 2218 | 1798 | 1193 | 885 |  |  |  | 41 | 8385.573 |
| **Wairoa District** | 337 | 159 | 113 | 133 | 6460 | 4329 | 2602 | 5312 | 875 | 475 | 454 | 366 |  |  | 56 | 28 | 4076.71 |
| **Hastings District** | 3948 |  |  | 1085 | 4701 | 2870 |  | 2214 | 2179 | 1514 | 1478 | 933 |  | 277 | 269 | 271 | 5226.564 |
| **Napier City** | 50 | 22 | 41 | 11 |  |  |  | 0 | 205 | 91 | 49 | 59 |  |  |  | 0 | 105.4925 |
| **Central Hawke's Bay District** | 3657 | 4420 |  | 649 | 2706 | 2326 | 2549 | 1566 | 1297 | 880 | 820 | 532 |  | 30 |  | 47 | 3331.769 |
| **New Plymouth District** | 4877 | 5829 |  | 526 | 3608 | 3723 | 2850 | 1631 | 1015 | 779 | 933 | 639 |  | 120 | 191 | 507 | 2205.463 |
| **Stratford District** |  |  |  | 273 | 2377 |  | 79 | 36 | 402 | 424 | 430 | 253 |  |  |  | 32 | 2163.372 |
| **South Taranaki District** |  |  | 11701 | 15274 | 474 |  | 322 | 329 | 1002 | 709 | 582 | 473 |  |  |  | 53 | 3575.038 |
| **Ruapehu District** | 406 | 643 | 298 | 116 | 3098 | 802 | 0 | 1964 | 891 | 781 | 679 | 590 |  |  | 430 | 779 | 6734.231 |
| **Wanganui District** |  |  |  | 3496 | 1342 | 82 | 93 | 90 | 1043 | 636 | 565 | 428 |  | 47 | 14 | 31 | 2373.539 |
| **Rangitikei District** |  |  |  | 269 | 583 |  | 349 | 276 | 1352 | 1077 | 771 | 459 |  |  |  | 64 | 4483.533 |
| **Manawatu District** | 14933 |  |  | 6283 | 942 | 404 | 380 | 1070 | 2219 | 1700 | 1550 | 767 |  | 140 | 168 | 49 | 2625.995 |
| **Palmerston North City** | 244 | 64 |  | 3296 | 195 |  | 152 | 245 | 408 | 406 | 337 | 270 |  |  | 562 | 408 | 335.7412 |
| **Tararua District** | 386 | 301 | 245 | 145 | 3331 | 588 | 316 | 181 | 1174 | 832 | 850 | 590 |  | 12 | 80 | 90 | 4364.361 |
| **Horowhenua District** |  |  |  | 3313 |  | 349 |  | 3012 | 861 | 552 | 530 | 264 |  | 179 | 269 | 47 | 1063.979 |
| **Kapiti Coast District** | 121 | 65 | 79 | 13 |  | 123 |  |  | 615 | 430 | 405 | 495 |  | 144 | 72 | 34 | 731.4221 |
| **Porirua City** | 35 |  | 74 | 91 |  |  |  | 13 | 107 | 139 | 145 | 92 |  |  |  | 127 | 174.7231 |
| **Upper Hutt City** |  | 46 | 44 | 4 | 87 |  | 29 | 17 | 128 | 79 | 105 | 60 |  | 0 |  | 0 | 539.8682 |
| **Lower Hutt City** | 116 |  |  | 0 |  |  |  | 0 | 98 | 161 | 63 | 5 |  |  |  | 0 | 376.4303 |
| **Wellington City** |  |  |  | 0 | 9 |  |  | 102 | 311 | 271 | 235 | 182 |  |  |  | 75 | 289.9669 |
| **Masterton District** |  | 78 | 118 | 32 | 590 | 104 | 173 | 106 | 837 | 732 | 594 | 638 |  |  | 171 | 88 | 2300.007 |
| **Carterton District** |  |  |  | 2379 | 394 |  |  |  | 248 | 195 | 196 | 124 |  |  |  | 89 | 1180.39 |
| **South Wairarapa District** |  |  |  | 4843 |  |  |  | 312 | 386 | 269 | 277 | 176 |  | 39 |  | 21 | 2386.487 |
| **Tasman District** | 761 | 322 | 500 | 207 | 3695 | 2949 | 2385 | 1667 | 763 | 656 | 706 | 457 |  | 415 | 505 | 242 | 9616.263 |
| **Nelson City** | 18 |  |  | 8 |  |  |  | 450 | 117 | 72 | 53 | 41 |  |  |  | 0 | 423.5983 |
| **Marlborough District** |  |  |  | 1398 | 4707 | 3679 | 1849 | 761 | 841 | 2501 | 638 | 257 |  | 169 | 160 | 35 | 10459.84 |
| **Kaikoura District** | 575 |  |  | 55 | 2204 |  |  | 87 | 174 | 158 | 130 | 38 |  |  |  | 0 | 2045.106 |
| **Buller District** | 73 | 53 |  | 9 | 459 |  | 289 | 86 | 169 | 114 | 65 | 55 |  |  | 14 | 6 | 7941.974 |
| **Grey District** |  | 33 | 39 | 9 | 104 |  |  |  | 129 | 97 | 52 | 49 |  | 0 |  | 0 | 3474.08 |
| **Westland District** |  | 124 |  | 53 | 122 | 67 |  | 132 | 238 | 109 | 98 | 60 |  |  |  | 0 | 11828.66 |
| **Hurunui District** | 3093 |  |  | 49825 | 1938 | 3066 | 2103 | 1398 | 1473 | 1404 | 1060 | 878 |  | 91 | 191 | 37 | 8640.998 |
| **Waimakariri District** | 22529 | 27641 | 32506 | 18844 | 2845 | 2015 | 1334 | 607 | 2033 | 2775 | 2624 | 1958 |  | 510 | 1069 | 741 | 2217.426 |
| **Christchurch City** | 12274 | 13389 | 10381 | 7539 | 979 | 1180 | 446 | 483 | 1658 | 1426 | 1030 | 551 |  | 317 | 640 | 190 | 1415.265 |
| **Selwyn District** | 60928 | 55705 | 53135 | 65545 | 4174 | 2967 | 1657 | 570 | 3870 | 4476 | 3671 | 2821 |  | 1291 | 1582 | 764 | 6380.546 |
| **Ashburton District** | 24127 | 36440 | 13942 | 17811 | 3164 | 1139 | 676 | 467 | 2215 | 1532 | 1349 | 973 |  | 572 | 427 | 143 | 6180.065 |
| **Timaru District** | 16878 | 22986 | 18432 | 6441 | 3407 | 2290 | 1696 | 792 | 944 | 681 | 683 | 301 |  | 114 | 111 | 29 | 2734.808 |
| **Mackenzie District** | 19 | 41 | 18 | 923 | 576 |  | 867 | 278 | 193 | 183 | 159 | 92 |  |  |  | 68 | 7140.417 |
| **Waimate District** | 10818 | 8363 | 5290 | 6049 | 1569 | 1203 |  | 219 | 424 | 543 | 529 | 274 |  | 60 | 122 | 33 | 3554.098 |
| **Waitaki District** | 7982 | 13852 | 6106 | 10272 | 1171 | 1035 |  | 186 | 852 | 643 | 568 | 492 |  |  | 62 | 52 | 7109.528 |
| **Central Otago District** |  | 103 |  | 17 | 4215 | 4297 | 3095 | 2232 | 746 | 463 | 353 | 254 |  |  |  | 9 | 9956.912 |
| **Queenstown-Lakes District** | 23 |  |  | 1 | 268 | 3314 |  | 5 | 281 | 283 | 167 | 85 |  | 59 |  | 19 | 8722.842 |
| **Dunedin City** |  |  |  | 382 | 599 | 902 | 1429 |  | 1360 | 1081 | 1152 | 694 |  | 277 | 372 | 360 | 3279.053 |
| **Clutha District** |  | 1412 | 1289 | 911 | 2693 | 1906 | 2650 | 588 | 817 | 570 | 573 | 347 |  | 298 |  | 453 | 6334.38 |
| **Southland District** | 5498 | 3824 | 1297 | 364 | 3467 | 3858 | 1649 | 918 | 3813 | 2585 | 2737 | 1973 |  | 202 | 291 | 170 | 29550.93 |
| **Gore District** |  | 422 |  | 27 | 992 |  | 197 |  | 792 | 427 | 400 | 316 |  |  |  | 33 | 1253.788 |
| **Invercargill City** |  | 57 |  | 17 | 118 |  | 34 | 20 | 492 | 354 | 331 | 177 |  |  |  | 5 | 385.8451 |

There were a few irregular missing records of animal counts due to confidentiality or data validity restrictions on the data assigned by StatsNZ, they were assumed to be negligible numbers and regarded as zero.

*Supplementary Table S4:* *Table demonstrating in red text the adjustment made to raw animal population data before it was utilised in analysis to account for the 2010 Auckland Council Territorial Authority Boundary change.*

| **Territorial Authority** | **Species and year** | | | | | | | | | | | | | | | |  |
| --- | --- | --- | --- | --- | --- | --- | --- | --- | --- | --- | --- | --- | --- | --- | --- | --- | --- |
|  | **Dairy Cattle** | | | | **Beef Cattle** | | | | **Sheep** | | | | **Deer** | | | |  |
|  | **2002** | **2007** | **2012** | **2017** | **2002** | **2007** | **2012** | **2017** | **2002** | **2007** | **2012** | **2017** | **2002** | **2007** | **2012** | **2017** |  |
| **Rodney District** | 74293 | 59323 | 59323 | 59323 | 97118 | 96739 | 96739 | 96739 | 207845 | 181579 | 181579 | 181579 | 13996 | 9624 | 9624 | 9624 |  |
| **North Shore City** | 207 |  |  |  |  |  |  |  |  |  |  |  |  | 0 | 0 | 0 |  |
| **Waitakere City** |  |  |  |  | 2906 | 3738 | 3738 | 3738 | 3698 |  |  |  |  | 0 | 0 | 0 |  |
| **Auckland City** | 1966 |  | * | * | 7609 | 3791 | 3791* | 3791* | 41664 | 33297 | 33297* | 33297* |  | 0 | 0* | 0* |  |
| **Manukau City** | 11019 | 5429 | 5429 | 5429 | 18366 | 13160 | 13160 | 13160 | 41485 | 39237 | 39237 | 39237 | 810 |  |  |  |  |
| **Papakura District** | 5549 | 2496 | 2496 | 2496 | 5754 |  |  |  | 7611 |  |  |  | 756 |  |  |  |  |
| **Franklin District** | 113471 | 104040 | 104040 | 104040 | 105371 | 102913 | 102913 | 102913 | 265565 | 240226 | 240226 | 240226 | 10459 | 7847 | 7847 | 7847 |  |
|  | **Pigs** | | | | **Goats** | | | | **Horse** | | | | **Alpaca** | | | |  |
|  | **2002** | **2007** | **2012** | **2017** | **2002** | **2007** | **2012** | **2017** | **2002** | **2007** | **2012** | **2017** | **2002** | **2007** | **2012** | **2017** | **Land_Area SQ_KM^6^** |
| **Rodney District** |  |  |  |  | 6654 | 2794 | 2794 | 2794 | 2128 | 2228 | 2228 | 2228 |  | 662 | 662 | 662 | 2372.484 |
| **North Shore City** |  | 0 | 0 | 0 |  |  |  |  |  | 0 | 0 | 0 |  | 0 | 0 | 0 | 127.8536 |
| **Waitakere City** |  |  |  |  |  |  |  |  | 213 | 105 | 105 | 105 |  |  |  |  | 367.1399 |
| **Auckland City** | 46 |  |  | * |  |  | * | * |  |  | * | * |  |  | * | * | 629.2666 |
| **Manukau City** | 94 | 46 | 46 | 46 | 1281 | 560 | 560 | 560 | 1657 | 1022 | 1022 | 1022 |  |  |  |  | 543.5892 |
| **Papakura District** |  |  |  |  | 52 |  |  |  | 849 | 598 | 598 | 598 |  |  |  |  | 118.589 |
| **Franklin District** | 17041 | 12686 | 12686 | 12686 | 3713 | 3044 | 3044 | 3044 | 4504 | 3848 | 3848 | 3848 |  | 278 | 278 | 278 | 2176.796 |

The animal population data available at the TA level were then aggregated at the DHB level, except for three districts (Western Bay of Plenty, Ruapehu, and Kapiti Coast) where the TA boundary straddled a DHB boundary. For these districts, the TA was assigned to the DHB containing the greatest proportion of their human population in alignment with StatsNZ policy.

*Auckland City Council data from the 2012 or 2017 Agricultural Production Survey for the corresponding year was not utilised. Over the period of the study the TA boundaries changed due to local government reforms. In 2010, the [Auckland City Council](https://en.wikipedia.org/wiki/Auckland_City_Council), [Manukau City Council](https://en.wikipedia.org/wiki/Manukau_City_Council), [Waitakere City Council](https://en.wikipedia.org/wiki/Waitakere_City_Council), [North Shore City Council](https://en.wikipedia.org/wiki/North_Shore_City_Council), [Papakura District Council](https://en.wikipedia.org/wiki/Papakura_District), [Rodney District Council](https://en.wikipedia.org/wiki/Rodney_District_Council) and [Franklin District Council](https://en.wikipedia.org/wiki/Franklin_District) were amalgamated into the [Auckland Council](https://en.wikipedia.org/wiki/Auckland_Council). However, the DHBs of Auckland, Counties Manukau, and Waitemata continued to align with the outer aggregated boundary of their contributing pre-2010 TAs. Subsequent to the amalgamation, the available animal population data at the TA level reflected the change in the Auckland Council boundary. Consequently, the animal population data for the affected Auckland Council territories were provided as a single figure for the new territory post 2010. To allow for the animal population to be appropriately attributed to each DHB within the Auckland Council boundary (Auckland, Counties Manukau and Waitemata), the separate animal numbers recorded in 2007 for the seven territories were used for the years 2008 to 2017.

*Supplementary Table S5: Table of the rainfall measurements (in millimetres per month) in each New Zealand District Health Board between 1 January 1996 to 31 December 2017 obtained from NIWA and used in the study analysis.*

| **Month** | **Auckland** | **Bay of Plenty** | **Canterbury** | **Capital and Coast** | **Counties Manukau** | **Hawke's Bay** | **Hutt Valley** | **Lakes** | **MidCentral** | **Nelson Marlborough** | **Northland** | **South Canterbury** | **Southern** | **Tairawhiti** | **Taranaki** | **Waikato** | **Wairarapa** | **Waitemata** | **West Coast** | **Whanganui** |
| --- | --- | --- | --- | --- | --- | --- | --- | --- | --- | --- | --- | --- | --- | --- | --- | --- | --- | --- | --- | --- |
| **Jan-96** | 22.4 | 116.6 | 41.4 | 31.3 | 39.3 | 141.1 | 26.7 | 80.7 | 47.4 | 86.7 | 81.7 | 74.0 | 121.2 | 216.4 | 49.9 | 90.2 | 59.4 | 41.0 | 103.7 | 84.6 |
| **Feb-96** | 54.5 | 100.8 | 90.3 | 125.9 | 56.6 | 118.5 | 177.8 | 141.3 | 137.4 | 100.4 | 67.9 | 122.3 | 101.9 | 200.7 | 122.5 | 156.3 | 136.9 | 75.1 | 292.4 | 115.0 |
| **Mar-96** | 91.0 | 110.8 | 85.0 | 81.4 | 128.8 | 104.9 | 98.1 | 143.0 | 88.2 | 134.5 | 74.6 | 87.2 | 107.6 | 227.8 | 92.5 | 144.6 | 87.8 | 99.8 | 287.2 | 101.7 |
| **Apr-96** | 171.1 | 164.3 | 174.8 | 93.9 | 176.5 | 95.1 | 131.3 | 175.1 | 132.8 | 159.6 | 68.1 | 138.9 | 247.4 | 93.1 | 189.4 | 218.6 | 91.6 | 126.9 | 435.4 | 130.2 |
| **May-96** | 161.2 | 136.6 | 81.3 | 121.4 | 158.9 | 71.9 | 159.3 | 113.0 | 136.1 | 92.6 | 93.0 | 44.9 | 146.0 | 122.1 | 118.1 | 167.8 | 114.9 | 136.8 | 242.0 | 119.6 |
| **Jun-96** | 202.2 | 108.3 | 91.5 | 97.3 | 167.2 | 87.9 | 154.2 | 99.6 | 116.1 | 85.3 | 145.1 | 74.6 | 135.0 | 120.3 | 110.3 | 174.9 | 144.5 | 192.0 | 184.6 | 99.4 |
| **Jul-96** | 257.1 | 198.1 | 166.0 | 180.6 | 178.2 | 181.9 | 151.8 | 143.4 | 116.5 | 190.9 | 201.1 | 74.1 | 54.3 | 201.2 | 144.1 | 212.5 | 228.5 | 217.7 | 87.8 | 104.3 |
| **Aug-96** | 241.2 | 188.2 | 82.9 | 96.1 | 161.4 | 59.7 | 123.1 | 119.7 | 112.3 | 138.8 | 163.9 | 42.8 | 84.8 | 146.5 | 137.9 | 212.3 | 93.2 | 190.2 | 218.1 | 116.8 |
| **Sep-96** | 112.3 | 155.7 | 69.9 | 105.9 | 135.7 | 42.3 | 138.7 | 160.2 | 113.8 | 143.1 | 150.2 | 40.3 | 110.7 | 176.6 | 157.4 | 195.2 | 41.6 | 117.3 | 340.3 | 98.1 |
| **Oct-96** | 64.1 | 62.9 | 126.3 | 105.0 | 55.9 | 34.0 | 166.0 | 43.4 | 135.8 | 125.3 | 136.4 | 92.1 | 227.3 | 99.1 | 114.3 | 103.8 | 32.1 | 64.6 | 600.0 | 89.8 |
| **Nov-96** | 67.7 | 40.8 | 103.4 | 89.1 | 79.3 | 41.8 | 136.2 | 63.5 | 123.1 | 160.8 | 41.9 | 68.3 | 133.3 | 77.9 | 126.1 | 133.6 | 81.2 | 68.1 | 322.6 | 123.4 |
| **Dec-96** | 257.6 | 234.4 | 82.7 | 67.5 | 150.1 | 126.5 | 121.0 | 124.7 | 111.6 | 60.3 | 173.3 | 60.9 | 117.6 | 290.5 | 112.5 | 196.9 | 72.0 | 190.5 | 190.7 | 103.9 |
| **Jan-97** | 17.0 | 48.3 | 95.2 | 54.2 | 15.3 | 63.3 | 50.3 | 38.1 | 73.7 | 66.0 | 44.8 | 77.6 | 64.8 | 78.8 | 56.3 | 41.6 | 63.9 | 35.0 | 71.0 | 69.9 |
| **Feb-97** | 53.7 | 80.8 | 115.2 | 48.5 | 38.9 | 69.7 | 75.0 | 102.0 | 80.5 | 84.9 | 74.6 | 75.9 | 138.0 | 143.5 | 61.2 | 90.4 | 80.6 | 57.2 | 333.4 | 44.5 |
| **Mar-97** | 79.1 | 158.5 | 141.8 | 101.6 | 88.6 | 136.9 | 103.0 | 120.2 | 102.0 | 76.8 | 110.4 | 90.0 | 90.3 | 263.5 | 79.3 | 147.2 | 98.4 | 85.6 | 129.8 | 78.6 |
| **Apr-97** | 68.5 | 63.6 | 119.8 | 93.1 | 81.5 | 51.2 | 105.8 | 64.7 | 133.2 | 99.1 | 92.6 | 82.0 | 214.4 | 74.4 | 137.2 | 117.3 | 37.9 | 76.9 | 265.4 | 123.6 |
| **May-97** | 140.1 | 103.7 | 81.6 | 22.4 | 112.7 | 57.1 | 27.3 | 114.5 | 32.5 | 82.1 | 97.3 | 39.1 | 107.6 | 98.2 | 75.9 | 126.6 | 33.1 | 133.4 | 248.1 | 35.8 |
| **Jun-97** | 189.7 | 241.8 | 89.0 | 105.9 | 141.2 | 225.2 | 84.3 | 166.0 | 68.7 | 138.7 | 285.7 | 20.4 | 48.4 | 239.7 | 98.1 | 148.8 | 99.7 | 172.7 | 146.4 | 75.1 |
| **Jul-97** | 56.2 | 113.8 | 84.0 | 99.3 | 64.8 | 114.0 | 129.8 | 78.1 | 44.5 | 78.1 | 89.2 | 43.4 | 124.5 | 144.8 | 81.3 | 92.4 | 111.0 | 56.7 | 191.7 | 58.6 |
| **Aug-97** | 113.3 | 97.8 | 98.9 | 141.4 | 83.0 | 124.7 | 172.3 | 71.5 | 83.4 | 109.5 | 111.4 | 90.8 | 171.6 | 160.1 | 78.4 | 139.8 | 113.5 | 109.8 | 300.7 | 90.3 |
| **Sep-97** | 318.8 | 160.5 | 55.7 | 61.8 | 223.1 | 87.9 | 78.4 | 147.3 | 86.4 | 101.0 | 297.6 | 54.4 | 44.6 | 110.4 | 107.1 | 253.1 | 75.9 | 240.9 | 65.1 | 78.5 |
| **Oct-97** | 76.5 | 78.4 | 60.6 | 98.6 | 100.3 | 90.7 | 207.6 | 78.0 | 116.8 | 77.7 | 68.8 | 49.4 | 147.9 | 53.6 | 168.3 | 108.5 | 127.1 | 77.6 | 261.2 | 105.8 |
| **Nov-97** | 81.7 | 53.6 | 53.4 | 21.3 | 62.4 | 27.3 | 68.1 | 62.8 | 82.3 | 89.2 | 41.1 | 49.7 | 225.0 | 82.1 | 132.6 | 149.5 | 28.6 | 78.5 | 458.9 | 94.0 |
| **Dec-97** | 64.3 | 54.9 | 81.7 | 46.2 | 78.6 | 13.5 | 131.6 | 37.2 | 127.1 | 96.2 | 109.9 | 86.3 | 201.2 | 57.2 | 184.7 | 112.0 | 18.0 | 57.5 | 408.7 | 92.2 |
| **Jan-98** | 7.0 | 22.9 | 32.9 | 52.2 | 12.5 | 24.6 | 98.4 | 41.9 | 61.6 | 69.8 | 21.2 | 20.2 | 107.8 | 17.4 | 102.9 | 76.4 | 20.1 | 26.2 | 416.2 | 63.8 |
| **Feb-98** | 86.8 | 94.0 | 45.6 | 34.3 | 116.4 | 39.9 | 63.6 | 68.4 | 79.0 | 97.3 | 59.6 | 49.7 | 200.1 | 79.2 | 144.2 | 110.0 | 28.3 | 107.7 | 392.6 | 89.4 |
| **Mar-98** | 97.1 | 134.1 | 112.6 | 24.0 | 83.0 | 8.1 | 59.3 | 115.6 | 44.0 | 124.8 | 63.1 | 87.4 | 229.6 | 85.9 | 63.2 | 78.4 | 17.0 | 101.7 | 387.7 | 34.5 |
| **Apr-98** | 72.4 | 84.4 | 48.7 | 29.4 | 39.2 | 39.6 | 44.5 | 61.0 | 84.1 | 69.4 | 81.8 | 43.2 | 132.6 | 74.6 | 91.2 | 85.9 | 32.9 | 35.2 | 263.9 | 77.9 |
| **May-98** | 97.7 | 83.3 | 58.5 | 78.9 | 83.1 | 49.3 | 82.0 | 114.5 | 98.2 | 145.4 | 122.4 | 56.3 | 112.6 | 33.7 | 156.9 | 133.6 | 50.8 | 97.7 | 196.5 | 96.7 |
| **Jun-98** | 164.1 | 176.6 | 84.1 | 129.9 | 107.9 | 44.5 | 146.7 | 124.3 | 164.9 | 168.0 | 184.5 | 56.9 | 132.1 | 146.6 | 157.2 | 192.5 | 81.6 | 137.1 | 305.9 | 85.6 |
| **Jul-98** | 404.2 | 407.5 | 139.0 | 156.7 | 296.0 | 174.0 | 255.4 | 311.4 | 199.0 | 326.7 | 355.5 | 81.5 | 145.4 | 220.0 | 268.8 | 384.4 | 116.7 | 380.3 | 367.9 | 199.5 |
| **Aug-98** | 147.5 | 115.5 | 91.1 | 64.5 | 145.0 | 43.5 | 54.7 | 117.1 | 64.4 | 163.1 | 170.1 | 56.8 | 105.7 | 92.4 | 70.0 | 138.3 | 80.0 | 124.5 | 232.1 | 67.0 |
| **Sep-98** | 72.3 | 90.5 | 67.1 | 79.6 | 75.2 | 11.5 | 114.9 | 65.0 | 111.5 | 141.1 | 78.2 | 32.1 | 154.2 | 51.8 | 151.7 | 113.3 | 38.3 | 74.8 | 289.5 | 78.8 |
| **Oct-98** | 114.1 | 139.4 | 191.7 | 264.6 | 110.6 | 26.7 | 397.1 | 113.1 | 270.9 | 374.4 | 104.8 | 134.9 | 208.2 | 38.3 | 503.1 | 223.4 | 117.1 | 94.7 | 587.2 | 220.2 |
| **Nov-98** | 82.8 | 109.0 | 31.9 | 58.0 | 68.0 | 82.8 | 47.8 | 64.8 | 96.3 | 79.2 | 146.7 | 38.3 | 42.2 | 125.7 | 107.3 | 82.8 | 65.4 | 92.4 | 51.9 | 67.1 |
| **Dec-98** | 96.8 | 109.8 | 63.5 | 52.3 | 37.7 | 44.3 | 52.7 | 98.1 | 56.6 | 103.1 | 64.5 | 46.7 | 115.3 | 51.1 | 108.4 | 106.7 | 34.2 | 61.8 | 410.2 | 47.8 |
| **Jan-99** | 103.4 | 136.8 | 56.9 | 63.9 | 169.2 | 150.0 | 111.8 | 123.7 | 81.6 | 71.6 | 132.9 | 66.6 | 87.8 | 160.5 | 149.5 | 116.9 | 69.0 | 71.4 | 186.4 | 125.4 |
| **Feb-99** | 30.0 | 19.3 | 66.2 | 34.7 | 33.6 | 21.0 | 47.3 | 8.1 | 24.4 | 44.4 | 42.7 | 44.4 | 57.5 | 23.1 | 17.8 | 24.2 | 30.5 | 44.4 | 196.1 | 14.2 |
| **Mar-99** | 60.2 | 125.1 | 129.5 | 99.1 | 58.5 | 131.1 | 99.2 | 81.2 | 64.6 | 104.1 | 123.3 | 72.1 | 176.7 | 136.5 | 117.7 | 90.0 | 68.5 | 46.3 | 306.4 | 74.5 |
| **Apr-99** | 126.3 | 142.5 | 93.3 | 55.2 | 112.1 | 96.9 | 89.0 | 104.6 | 69.3 | 136.4 | 329.3 | 75.3 | 126.9 | 119.7 | 81.0 | 124.7 | 85.1 | 151.7 | 277.6 | 86.3 |
| **May-99** | 71.4 | 81.6 | 75.0 | 179.5 | 66.4 | 73.8 | 208.1 | 99.1 | 131.8 | 94.3 | 54.0 | 17.1 | 170.8 | 129.6 | 170.0 | 122.0 | 82.8 | 42.6 | 235.5 | 104.0 |
| **Jun-99** | 123.8 | 150.0 | 119.4 | 100.7 | 99.7 | 99.3 | 91.9 | 102.4 | 77.4 | 147.4 | 124.7 | 85.5 | 90.9 | 200.5 | 138.9 | 124.1 | 72.2 | 129.9 | 161.5 | 73.0 |
| **Jul-99** | 176.3 | 139.5 | 142.9 | 125.3 | 136.8 | 103.4 | 150.7 | 117.2 | 119.7 | 141.0 | 136.1 | 97.4 | 142.8 | 108.4 | 179.7 | 167.3 | 137.4 | 112.4 | 288.8 | 112.5 |
| **Aug-99** | 135.3 | 139.3 | 65.1 | 75.6 | 140.7 | 58.9 | 115.4 | 92.9 | 103.1 | 149.5 | 94.4 | 26.7 | 89.4 | 154.9 | 169.3 | 144.2 | 39.8 | 127.9 | 185.4 | 75.8 |
| **Sep-99** | 95.5 | 142.7 | 42.6 | 65.1 | 58.8 | 62.6 | 90.7 | 102.0 | 83.6 | 114.9 | 92.3 | 33.4 | 94.0 | 134.7 | 109.9 | 124.4 | 32.1 | 61.1 | 199.6 | 75.7 |
| **Oct-99** | 114.5 | 66.7 | 121.8 | 97.1 | 75.5 | 30.5 | 119.4 | 26.4 | 51.8 | 110.0 | 55.8 | 57.2 | 150.9 | 74.4 | 68.4 | 68.9 | 61.4 | 85.6 | 493.6 | 36.6 |
| **Nov-99** | 150.8 | 250.0 | 175.4 | 166.1 | 178.6 | 146.9 | 172.5 | 224.9 | 167.1 | 257.8 | 221.2 | 133.5 | 259.0 | 173.0 | 220.8 | 216.4 | 154.9 | 165.3 | 259.9 | 181.6 |
| **Dec-99** | 77.3 | 92.6 | 57.5 | 44.3 | 31.6 | 48.8 | 51.1 | 66.5 | 108.5 | 90.0 | 82.6 | 100.8 | 76.9 | 148.0 | 73.9 | 78.2 | 74.1 | 39.2 | 157.8 | 42.1 |
| **Jan-00** | 65.9 | 77.4 | 105.0 | 106.8 | 124.6 | 69.9 | 118.3 | 66.1 | 136.0 | 167.9 | 46.5 | 124.0 | 156.0 | 91.9 | 126.7 | 151.4 | 96.5 | 90.9 | 299.0 | 147.1 |
| **Feb-00** | 31.8 | 23.6 | 43.4 | 28.5 | 10.2 | 14.6 | 28.0 | 2.8 | 22.2 | 92.1 | 44.7 | 45.0 | 98.4 | 49.2 | 36.4 | 11.5 | 19.3 | 22.0 | 165.6 | 15.5 |
| **Mar-00** | 68.6 | 92.8 | 99.2 | 47.5 | 57.8 | 94.2 | 49.8 | 32.7 | 52.5 | 46.0 | 54.4 | 111.4 | 106.5 | 224.0 | 49.5 | 50.5 | 66.8 | 52.7 | 123.6 | 34.2 |
| **Apr-00** | 134.4 | 225.6 | 130.7 | 105.2 | 110.8 | 129.7 | 121.7 | 159.0 | 91.8 | 199.8 | 116.6 | 117.8 | 119.1 | 202.5 | 176.4 | 172.6 | 67.6 | 102.3 | 390.6 | 117.8 |
| **May-00** | 78.6 | 154.9 | 99.3 | 89.7 | 112.8 | 66.1 | 104.4 | 79.7 | 109.4 | 142.7 | 131.9 | 85.2 | 212.1 | 140.7 | 159.3 | 108.1 | 69.2 | 108.8 | 422.9 | 69.9 |
| **Jun-00** | 105.8 | 99.2 | 104.7 | 114.7 | 142.3 | 61.1 | 157.1 | 79.7 | 96.6 | 152.8 | 164.9 | 74.3 | 185.2 | 91.2 | 131.7 | 132.8 | 108.2 | 189.5 | 301.9 | 88.2 |
| **Jul-00** | 180.9 | 131.5 | 74.0 | 39.7 | 147.4 | 138.0 | 49.0 | 132.8 | 49.0 | 123.4 | 250.3 | 39.4 | 91.3 | 161.9 | 69.6 | 163.2 | 50.3 | 201.5 | 206.8 | 68.0 |
| **Aug-00** | 176.8 | 201.6 | 197.7 | 67.7 | 107.2 | 59.2 | 79.3 | 187.8 | 82.7 | 178.8 | 107.9 | 193.8 | 112.7 | 107.7 | 90.1 | 135.3 | 85.4 | 100.7 | 182.3 | 81.5 |
| **Sep-00** | 57.4 | 87.4 | 132.1 | 108.2 | 76.3 | 88.7 | 188.6 | 75.5 | 110.6 | 113.4 | 54.6 | 133.1 | 152.6 | 118.5 | 187.8 | 197.3 | 93.8 | 84.1 | 297.8 | 120.1 |
| **Oct-00** | 61.8 | 75.1 | 122.6 | 87.0 | 69.9 | 22.3 | 167.2 | 63.4 | 143.2 | 146.7 | 50.4 | 43.7 | 176.0 | 104.0 | 126.8 | 160.4 | 76.7 | 55.2 | 366.5 | 133.2 |
| **Nov-00** | 168.6 | 105.0 | 146.7 | 43.9 | 99.4 | 100.0 | 41.6 | 66.8 | 54.9 | 50.2 | 143.7 | 91.4 | 86.4 | 188.6 | 70.7 | 109.0 | 58.0 | 87.2 | 115.6 | 65.7 |
| **Dec-00** | 84.4 | 92.8 | 86.6 | 57.0 | 69.6 | 35.5 | 76.5 | 96.9 | 120.3 | 94.2 | 106.4 | 72.6 | 194.0 | 63.2 | 114.1 | 142.3 | 57.7 | 79.6 | 317.1 | 113.5 |
| **Jan-01** | 57.5 | 49.2 | 61.9 | 8.5 | 46.7 | 39.1 | 25.5 | 34.1 | 43.6 | 40.8 | 100.3 | 64.8 | 92.2 | 117.7 | 31.1 | 50.1 | 28.7 | 63.6 | 135.9 | 70.1 |
| **Feb-01** | 237.0 | 266.4 | 17.3 | 22.2 | 124.3 | 76.1 | 28.4 | 165.0 | 55.5 | 11.6 | 79.6 | 17.6 | 57.8 | 183.4 | 73.8 | 182.5 | 34.8 | 122.7 | 154.5 | 77.4 |
| **Mar-01** | 21.1 | 57.8 | 41.1 | 23.0 | 37.9 | 32.1 | 48.2 | 55.2 | 65.5 | 27.6 | 53.0 | 16.9 | 87.7 | 79.4 | 59.8 | 68.5 | 13.9 | 19.7 | 199.2 | 27.7 |
| **Apr-01** | 214.4 | 187.8 | 26.6 | 14.5 | 103.6 | 81.1 | 25.4 | 98.0 | 58.0 | 51.5 | 195.5 | 20.0 | 74.6 | 131.9 | 68.4 | 124.4 | 24.3 | 132.3 | 100.9 | 96.9 |
| **May-01** | 230.0 | 203.3 | 86.6 | 59.4 | 220.0 | 66 .2 | 71.0 | 240.0 | 123.5 | 195.2 | 220.2 | 53.9 | 123.6 | 195.5 | 148.6 | 254.5 | 89.2 | 204.1 | 230.8 | 136.4 |
| **Jun-01** | 34.7 | 64.5 | 88.2 | 87.6 | 89.1 | 68.6 | 108.8 | 38.3 | 62.4 | 113.6 | 74.1 | 56.1 | 144.8 | 69.5 | 115.1 | 69.0 | 64.3 | 59.3 | 345.6 | 84.0 |
| **Jul-01** | 65.8 | 77.3 | 126.5 | 114.3 | 108.0 | 125.7 | 132.8 | 48.8 | 79.3 | 37.0 | 162.1 | 71.8 | 63.5 | 115.3 | 105.2 | 114.4 | 84.8 | 94.9 | 50.1 | 93.0 |
| **Aug-01** | 197.4 | 110.0 | 60.3 | 94.5 | 135.0 | 98.1 | 129.9 | 70.7 | 147.9 | 151.0 | 148.1 | 37.3 | 101.8 | 141.1 | 140.6 | 138.8 | 102.5 | 118.3 | 255.5 | 135.6 |
| **Sep-01** | 161.5 | 119.5 | 28.6 | 9.8 | 58.3 | 32.7 | 14.4 | 52.9 | 21.1 | 49.5 | 120.0 | 17.0 | 79.2 | 114.4 | 19.5 | 64.3 | 18.8 | 89.8 | 186.0 | 11.5 |
| **Oct-01** | 95.7 | 159.2 | 110.1 | 150.8 | 151.7 | 114.0 | 159.8 | 136.6 | 145.2 | 256.3 | 139.0 | 76.0 | 102.5 | 210.0 | 204.6 | 134.2 | 107.6 | 100.3 | 287.1 | 110.0 |
| **Nov-01** | 107.2 | 118.8 | 132.7 | 147.3 | 183.3 | 49.5 | 174.0 | 130.8 | 135.7 | 170.8 | 142.2 | 99.1 | 150.0 | 119.8 | 142.1 | 161.4 | 101.3 | 107.7 | 468.1 | 103.1 |
| **Dec-01** | 296.5 | 281.9 | 138.2 | 166.9 | 189.4 | 171.0 | 198.4 | 169.8 | 205.2 | 180.5 | 196.3 | 81.9 | 163.0 | 316.9 | 193.1 | 273.8 | 137.5 | 165.7 | 510.0 | 222.2 |
| **Jan-02** | 80.7 | 104.5 | 223.1 | 89.4 | 72.8 | 77.6 | 79.0 | 107.6 | 78.4 | 155.6 | 54.7 | 197.7 | 122.1 | 149.8 | 151.3 | 118.8 | 65.0 | 79.3 | 256.9 | 98.7 |
| **Feb-02** | 85.9 | 45.5 | 64.3 | 85.9 | 35.6 | 105.8 | 82.1 | 45.9 | 105.2 | 73.7 | 78.6 | 47.9 | 100.2 | 74.9 | 143.7 | 66.7 | 54.7 | 51.1 | 185.1 | 122.6 |
| **Mar-02** | 123.4 | 48.1 | 79.2 | 60.4 | 110.9 | 29.5 | 111.3 | 57.0 | 107.1 | 76.2 | 89.0 | 35.2 | 136.9 | 51.3 | 86.9 | 105.2 | 43.5 | 111.7 | 326.5 | 74.4 |
| **Apr-02** | 64.9 | 150.7 | 79.8 | 47.4 | 52.0 | 57.5 | 74.6 | 100.3 | 43.1 | 59.2 | 95.9 | 80.5 | 103.2 | 107.6 | 74.7 | 96.9 | 57.1 | 59.5 | 185.7 | 70.8 |
| **May-02** | 61.6 | 60.5 | 38.1 | 60.9 | 86.8 | 27.7 | 62.7 | 55.4 | 62.7 | 105.5 | 168.8 | 28.5 | 114.9 | 57.2 | 137.4 | 126.7 | 30.1 | 73.6 | 189.4 | 106.2 |
| **Jun-02** | 177.4 | 168.8 | 179.0 | 152.3 | 111.9 | 107.4 | 214.1 | 128.7 | 154.5 | 217.1 | 272.9 | 113.9 | 213.6 | 220.6 | 248.0 | 206.4 | 91.0 | 172.8 | 544.8 | 141.5 |
| **Jul-02** | 173.8 | 132.9 | 43.6 | 107.4 | 103.3 | 183.5 | 135.4 | 106.8 | 159.3 | 45.5 | 171.7 | 25.2 | 69.7 | 239.2 | 125.7 | 173.9 | 153.3 | 155.3 | 110.4 | 105.8 |
| **Aug-02** | 58.6 | 81.3 | 62.7 | 118.8 | 82.0 | 99.3 | 122.3 | 61.7 | 93.3 | 89.6 | 102.2 | 60.1 | 148.9 | 167.0 | 155.2 | 123.0 | 73.7 | 86.9 | 257.7 | 110.9 |
| **Sep-02** | 58.6 | 83.1 | 109.6 | 107.0 | 72.0 | 60.9 | 131.2 | 83.7 | 96.7 | 195.1 | 92.6 | 81.4 | 234.9 | 104.1 | 197.7 | 153.5 | 48.8 | 110.0 | 426.4 | 145.3 |
| **Oct-02** | 62.4 | 60.6 | 78.1 | 35.4 | 83.8 | 42.6 | 70.4 | 48.1 | 80.7 | 84.9 | 61.7 | 51.7 | 137.3 | 98.3 | 102.5 | 91.2 | 39.1 | 68.2 | 322.0 | 79.7 |
| **Nov-02** | 66.6 | 38.7 | 146.4 | 106.6 | 57.8 | 72.3 | 111.3 | 47.9 | 66.3 | 82.1 | 47.7 | 76.4 | 117.7 | 79.9 | 144.4 | 118.6 | 69.4 | 72.3 | 214.2 | 106.9 |
| **Dec-02** | 78.5 | 79.7 | 77.2 | 99.1 | 96.2 | 68.9 | 148.7 | 107.9 | 98.3 | 112.2 | 70.0 | 62.3 | 161.1 | 129.4 | 150.2 | 138.1 | 41.6 | 82.3 | 253.7 | 132.7 |
| **Jan-03** | 135.5 | 103.3 | 79.1 | 38.7 | 126.8 | 34.8 | 44.0 | 76.3 | 27.8 | 71.3 | 152.1 | 61.8 | 109.2 | 96.9 | 40.3 | 110.2 | 29.6 | 140.4 | 202.8 | 25.7 |
| **Feb-03** | 68.6 | 106.7 | 63.3 | 27.2 | 48.0 | 86.6 | 23.3 | 24.7 | 22.1 | 21.9 | 70.3 | 68.1 | 85.0 | 176.4 | 34.0 | 70.4 | 27.5 | 55.8 | 162.3 | 29.1 |
| **Mar-03** | 154.2 | 124.1 | 80.1 | 27.7 | 121.5 | 64.3 | 44.6 | 103.5 | 29.2 | 91.4 | 246.0 | 58.8 | 49.9 | 145.3 | 106.0 | 115.9 | 15.8 | 159.6 | 103.2 | 20.2 |
| **Apr-03** | 119.5 | 122.2 | 93.0 | 43.1 | 40.9 | 81.5 | 35.0 | 52.1 | 28.8 | 45.3 | 79.0 | 47.3 | 51.7 | 119.1 | 80.1 | 68.4 | 35.2 | 106.9 | 73.1 | 41.7 |
| **May-03** | 127.9 | 143.5 | 90.0 | 57.5 | 82.4 | 77.0 | 73.9 | 89.2 | 105.2 | 134.7 | 176.0 | 86.5 | 158.5 | 146.0 | 172.5 | 138.7 | 43.6 | 97.4 | 409.8 | 98.9 |
| **Jun-03** | 122.6 | 198.0 | 99.7 | 168.1 | 112.0 | 60.5 | 182.1 | 139.0 | 168.0 | 227.6 | 105.6 | 59.3 | 178.8 | 238.9 | 226.2 | 187.4 | 100.2 | 131.4 | 435.2 | 129.4 |
| **Jul-03** | 110.7 | 50.8 | 55.6 | 73.3 | 84.5 | 55.9 | 91.4 | 24.3 | 87.3 | 42.3 | 117.8 | 25.8 | 96.3 | 80.3 | 157.1 | 90.4 | 67.3 | 114.5 | 110.1 | 40.3 |
| **Aug-03** | 140.7 | 87.3 | 83.7 | 80.0 | 46.0 | 204.7 | 57.9 | 45.1 | 28.8 | 101.5 | 200.6 | 41.5 | 92.7 | 133.9 | 66.4 | 52.1 | 103.5 | 111.4 | 170.9 | 29.9 |
| **Sep-03** | 109.9 | 194.8 | 242.2 | 186.5 | 113.9 | 171.1 | 211.8 | 148.9 | 188.4 | 180.7 | 139.7 | 120.7 | 173.2 | 270.1 | 282.2 | 242.2 | 171.9 | 172.7 | 329.5 | 215.6 |
| **Oct-03** | 120.6 | 150.4 | 77.6 | 155.4 | 74.0 | 45.9 | 200.4 | 92.1 | 104.5 | 145.5 | 130.9 | 46.5 | 95.1 | 160.1 | 171.5 | 155.3 | 90.7 | 128.2 | 167.9 | 97.7 |
| **Nov-03** | 64.7 | 66.3 | 81.5 | 66.1 | 74.2 | 71.2 | 65.7 | 87.6 | 104.2 | 75.5 | 48.3 | 57.0 | 143.4 | 98.2 | 152.0 | 152.4 | 89.3 | 73.0 | 285.3 | 128.7 |
| **Dec-03** | 81.3 | 102.3 | 41.4 | 74.9 | 84.7 | 85.5 | 120.5 | 190.9 | 96.7 | 77.7 | 50.5 | 25.8 | 117.7 | 131.8 | 185.7 | 166.5 | 45.3 | 89.2 | 267.9 | 154.8 |
| **Jan-04** | 62.9 | 65.9 | 115.4 | 116.3 | 75.7 | 116.9 | 126.7 | 64.6 | 95.3 | 118.2 | 94.5 | 97.8 | 138.8 | 72.0 | 118.3 | 92.5 | 105.1 | 79.0 | 383.3 | 96.4 |
| **Feb-04** | 234.7 | 284.5 | 121.4 | 353.8 | 240.9 | 179.7 | 423.2 | 234.3 | 346.7 | 252.1 | 231.8 | 77.4 | 169.7 | 256.7 | 432.5 | 360.0 | 247.7 | 193.7 | 402.2 | 308.8 |
| **Mar-04** | 7.7 | 35.4 | 68.9 | 50.1 | 10.2 | 47.2 | 73.0 | 15.1 | 42.8 | 29.9 | 7.2 | 56.4 | 120.9 | 65.8 | 54.6 | 23.2 | 48.7 | 10.3 | 302.7 | 42.4 |
| **Apr-04** | 21.1 | 152.5 | 52.0 | 66.6 | 34.5 | 50.5 | 61.1 | 51.0 | 54.7 | 124.5 | 37.9 | 20.1 | 94.3 | 87.1 | 108.5 | 55.1 | 56.3 | 45.1 | 87.7 | 46.3 |
| **May-04** | 211.7 | 211.7 | 131.8 | 75.4 | 175.0 | 111.0 | 68.4 | 144.8 | 69.4 | 231.9 | 162.8 | 98.7 | 151.8 | 219.7 | 127.3 | 194.5 | 55.8 | 194.4 | 309.5 | 73.6 |
| **Jun-04** | 104.1 | 192.6 | 102.9 | 94.5 | 105.2 | 136.5 | 155.4 | 166.2 | 184.4 | 205.7 | 132.8 | 53.6 | 193.2 | 305.2 | 198.9 | 200.2 | 114.6 | 108.3 | 415.2 | 157.4 |
| **Jul-04** | 111.9 | 233.5 | 64.8 | 131.0 | 87.6 | 139.1 | 118.6 | 178.6 | 94.0 | 119.4 | 94.4 | 43.4 | 68.3 | 212.3 | 121.2 | 140.1 | 104.6 | 113.2 | 137.1 | 90.5 |
| **Aug-04** | 70.6 | 98.7 | 161.9 | 187.7 | 76.0 | 129.3 | 228.4 | 48.3 | 104.4 | 140.7 | 90.6 | 86.6 | 179.5 | 150.0 | 142.1 | 154.7 | 190.6 | 78.3 | 360.0 | 118.4 |
| **Sep-04** | 73.3 | 104.4 | 105.4 | 132.4 | 88.2 | 39.2 | 176.5 | 87.3 | 154.5 | 157.4 | 61.5 | 51.1 | 123.2 | 103.1 | 175.6 | 130.4 | 76.1 | 88.7 | 313.1 | 102.9 |
| **Oct-04** | 90.2 | 134.5 | 79.7 | 74.2 | 81.7 | 94.9 | 153.4 | 143.9 | 104.6 | 169.7 | 87.9 | 58.1 | 82.3 | 124.2 | 122.5 | 167.5 | 67.5 | 83.1 | 197.5 | 102.1 |
| **Nov-04** | 50.1 | 84.5 | 57.5 | 81.6 | 43.0 | 35.6 | 107.1 | 74.5 | 88.7 | 109.6 | 63.0 | 65.8 | 139.6 | 99.8 | 90.8 | 97.3 | 53.7 | 45.6 | 242.1 | 85.1 |
| **Dec-04** | 136.9 | 178.4 | 148.7 | 130.7 | 125.8 | 100.3 | 193.3 | 166.4 | 182.1 | 112.0 | 132.7 | 168.2 | 166.1 | 145.4 | 144.6 | 187.5 | 158.4 | 149.3 | 292.9 | 164.4 |
| **Jan-05** | 24.7 | 25.4 | 41.5 | 106.4 | 17.2 | 34.1 | 150.7 | 20.6 | 92.7 | 108.2 | 40.0 | 47.8 | 116.2 | 49.7 | 85.3 | 56.1 | 49.9 | 27.5 | 170.0 | 58.0 |
| **Feb-05** | 48.3 | 59.5 | 63.3 | 30.6 | 63.9 | 35.1 | 29.3 | 48.4 | 47.6 | 49.5 | 85.9 | 80.6 | 128.6 | 94.6 | 71.3 | 67.1 | 25.2 | 60.7 | 255.4 | 49.1 |
| **Mar-05** | 30.9 | 161.9 | 130.4 | 120.2 | 43.7 | 157.6 | 127.3 | 120.2 | 64.1 | 169.6 | 17.4 | 64.1 | 168.1 | 145.3 | 196.7 | 95.8 | 210.3 | 40.6 | 261.9 | 96.7 |
| **Apr-05** | 21.6 | 16.3 | 48.8 | 54.8 | 33.2 | 72.0 | 64.4 | 13.2 | 47.4 | 7.2 | 10.4 | 33.7 | 86.7 | 81.6 | 57.5 | 34.7 | 48.6 | 44.7 | 109.2 | 47.8 |
| **May-05** | 152.1 | 435.4 | 122.2 | 156.4 | 141.1 | 142.9 | 152.0 | 203.5 | 102.6 | 126.7 | 120.1 | 58.9 | 145.0 | 211.9 | 231.2 | 232.7 | 116.0 | 162.0 | 231.4 | 108.0 |
| **Jun-05** | 139.2 | 116.8 | 38.7 | 53.3 | 109.1 | 139.8 | 66.0 | 74.6 | 51.1 | 83.5 | 133.7 | 11.1 | 96.8 | 164.4 | 76.5 | 106.8 | 82.1 | 113.9 | 229.3 | 75.3 |
| **Jul-05** | 205.3 | 159.9 | 118.3 | 96.4 | 146.4 | 131.3 | 117.5 | 90.0 | 88.0 | 167.8 | 184.8 | 28.4 | 96.4 | 162.1 | 192.2 | 177.7 | 104.4 | 185.4 | 272.6 | 81.5 |
| **Aug-05** | 67.6 | 92.3 | 34.3 | 33.4 | 37.6 | 44.0 | 22.7 | 86.1 | 34.3 | 65.5 | 59.5 | 40.3 | 93.8 | 96.7 | 48.6 | 81.1 | 28.2 | 46.3 | 222.2 | 37.7 |
| **Sep-05** | 73.9 | 82.1 | 63.9 | 38.8 | 84.8 | 46.3 | 47.0 | 100.5 | 101.9 | 57.0 | 111.2 | 53.4 | 112.9 | 92.9 | 144.4 | 142.4 | 37.1 | 89.2 | 385.2 | 107.1 |
| **Oct-05** | 175.4 | 194.8 | 93.0 | 80.8 | 177.5 | 190.3 | 88.7 | 148.6 | 111.7 | 60.7 | 135.3 | 80.0 | 77.7 | 272.6 | 152.5 | 207.4 | 147.7 | 190.4 | 196.6 | 142.5 |
| **Nov-05** | 55.6 | 47.7 | 55.0 | 22.6 | 38.6 | 71.7 | 18.2 | 29.9 | 45.6 | 29.0 | 39.3 | 67.2 | 102.2 | 178.1 | 55.2 | 64.7 | 29.9 | 61.8 | 172.1 | 44.1 |
| **Dec-05** | 72.9 | 143.0 | 56.6 | 80.5 | 82.0 | 31.3 | 83.4 | 175.3 | 117.2 | 92.1 | 92.6 | 63.0 | 139.8 | 81.8 | 178.4 | 201.3 | 52.5 | 78.3 | 272.6 | 149.8 |
| **Jan-06** | 120.4 | 130.6 | 94.4 | 39.0 | 69.9 | 67.8 | 78.6 | 177.7 | 60.9 | 116.9 | 129.0 | 68.2 | 183.9 | 99.6 | 102.8 | 115.3 | 42.4 | 86.8 | 354.9 | 87.6 |
| **Feb-06** | 17.5 | 163.3 | 59.9 | 81.6 | 6.6 | 39.0 | 82.7 | 144.6 | 61.9 | 69.3 | 29.8 | 41.2 | 91.7 | 109.2 | 59.9 | 57.0 | 42.9 | 11.8 | 142.2 | 51.3 |
| **Mar-06** | 79.1 | 149.6 | 88.5 | 86.1 | 53.3 | 144.4 | 74.9 | 110.0 | 59.5 | 44.7 | 170.1 | 33.3 | 104.3 | 181.1 | 66.1 | 98.5 | 116.4 | 74.4 | 151.0 | 65.8 |
| **Apr-06** | 222.2 | 219.5 | 142.5 | 70.2 | 150.1 | 169.5 | 98.0 | 190.8 | 125.7 | 256.4 | 180.0 | 83.3 | 163.3 | 198.5 | 161.1 | 210.5 | 92.2 | 204.0 | 421.4 | 122.7 |
| **May-06** | 183.5 | 126.9 | 99.3 | 105.7 | 186.1 | 88.5 | 137.2 | 113.9 | 107.8 | 184.1 | 154.6 | 100.1 | 102.1 | 198.0 | 130.1 | 128.4 | 101.7 | 182.9 | 212.4 | 95.6 |
| **Jun-06** | 77.7 | 122.1 | 153.3 | 129.7 | 112.1 | 137.5 | 149.5 | 90.5 | 123.4 | 111.3 | 98.9 | 109.7 | 107.6 | 291.9 | 130.4 | 118.6 | 124.1 | 120.4 | 288.8 | 116.8 |
| **Jul-06** | 45.7 | 132.5 | 95.7 | 209.4 | 86.3 | 183.8 | 222.2 | 93.9 | 175.7 | 91.2 | 68.4 | 42.4 | 103.0 | 238.6 | 193.2 | 135.3 | 297.2 | 68.1 | 208.0 | 168.3 |
| **Aug-06** | 104.3 | 179.1 | 76.4 | 175.9 | 107.5 | 66.5 | 228.7 | 138.0 | 126.2 | 102.3 | 125.9 | 34.0 | 101.4 | 170.3 | 209.2 | 167.1 | 110.8 | 109.7 | 175.4 | 139.1 |
| **Sep-06** | 73.1 | 64.4 | 45.3 | 33.0 | 43.0 | 40.9 | 57.2 | 31.2 | 75.4 | 36.3 | 73.3 | 18.0 | 146.6 | 83.7 | 63.6 | 47.1 | 17.8 | 103.6 | 264.5 | 50.2 |
| **Oct-06** | 89.2 | 96.4 | 198.7 | 186.2 | 168.0 | 68.0 | 214.6 | 79.4 | 182.7 | 144.9 | 73.0 | 70.9 | 130.2 | 118.3 | 150.9 | 136.7 | 146.2 | 154.6 | 300.7 | 114.5 |
| **Nov-06** | 60.1 | 86.9 | 193.3 | 155.9 | 99.4 | 41.4 | 262.7 | 87.1 | 165.2 | 190.1 | 66.7 | 140.4 | 207.0 | 143.4 | 212.5 | 156.6 | 81.3 | 87.1 | 429.1 | 150.1 |
| **Dec-06** | 70.2 | 85.4 | 127.1 | 76.7 | 45.9 | 75.3 | 94.2 | 80.7 | 115.2 | 52.1 | 37.2 | 147.9 | 133.8 | 114.8 | 142.3 | 81.5 | 58.2 | 46.3 | 227.3 | 82.4 |
| **Jan-07** | 76.1 | 130.1 | 58.5 | 95.8 | 69.3 | 49.9 | 85.6 | 130.1 | 63.1 | 116.5 | 71.5 | 43.0 | 108.6 | 87.5 | 75.3 | 110.1 | 42.6 | 58.8 | 250.5 | 65.8 |
| **Feb-07** | 11.7 | 49.0 | 37.0 | 14.6 | 22.2 | 36.6 | 15.9 | 12.0 | 23.2 | 17.1 | 131.3 | 34.3 | 40.3 | 36.9 | 35.4 | 39.0 | 12.6 | 3.5 | 55.0 | 24.9 |
| **Mar-07** | 233.4 | 148.5 | 42.1 | 35.4 | 129.0 | 27.6 | 64.3 | 138.7 | 75.6 | 72.8 | 211.6 | 64.2 | 141.7 | 91.0 | 143.7 | 160.5 | 41.4 | 200.2 | 254.3 | 114.2 |
| **Apr-07** | 52.9 | 90.8 | 61.1 | 59.7 | 71.9 | 58.7 | 49.5 | 68.3 | 51.3 | 104.4 | 74.4 | 41.7 | 85.1 | 76.6 | 76.0 | 86.4 | 41.9 | 56.4 | 89.1 | 66.8 |
| **May-07** | 15.2 | 33.8 | 62.1 | 57.6 | 21.6 | 6.0 | 50.7 | 24.7 | 54.0 | 102.0 | 26.5 | 58.1 | 163.8 | 29.1 | 66.7 | 51.5 | 8.4 | 22.4 | 289.2 | 32.0 |
| **Jun-07** | 77.3 | 117.6 | 104.3 | 69.4 | 100.9 | 141.7 | 82.6 | 87.5 | 89.4 | 179.3 | 137.7 | 61.0 | 122.8 | 191.5 | 164.3 | 167.7 | 117.9 | 102.8 | 304.5 | 111.2 |
| **Jul-07** | 254.2 | 203.2 | 59.3 | 137.1 | 173.8 | 238.2 | 135.7 | 151.7 | 107.3 | 90.2 | 374.3 | 48.8 | 104.9 | 276.1 | 196.6 | 236.7 | 131.5 | 203.8 | 128.6 | 108.2 |
| **Aug-07** | 113.1 | 95.7 | 56.2 | 65.1 | 111.4 | 69.1 | 88.9 | 107.7 | 124.7 | 105.9 | 130.9 | 39.2 | 126.6 | 137.4 | 164.9 | 178.1 | 63.9 | 118.0 | 169.7 | 137.6 |
| **Sep-07** | 53.6 | 84.2 | 66.8 | 48.4 | 79.1 | 82.5 | 58.3 | 74.5 | 58.6 | 81.3 | 135.6 | 78.0 | 119.4 | 156.2 | 60.1 | 88.9 | 77.9 | 73.0 | 264.9 | 64.1 |
| **Oct-07** | 143.7 | 89.1 | 198.4 | 153.1 | 86.1 | 56.3 | 220.9 | 64.7 | 110.0 | 262.0 | 58.3 | 71.5 | 210.7 | 135.0 | 177.2 | 155.5 | 111.2 | 125.8 | 411.1 | 146.6 |
| **Nov-07** | 56.1 | 47.3 | 36.7 | 30.9 | 57.7 | 28.9 | 57.2 | 21.1 | 80.7 | 20.4 | 77.4 | 44.6 | 56.7 | 33.2 | 72.9 | 70.0 | 27.7 | 65.2 | 65.1 | 52.6 |
| **Dec-07** | 73.1 | 97.0 | 70.5 | 57.7 | 81.2 | 114.6 | 84.6 | 79.3 | 67.2 | 98.8 | 161.6 | 60.5 | 123.1 | 137.5 | 112.2 | 119.2 | 80.0 | 98.0 | 429.3 | 108.7 |
| **Jan-08** | 19.3 | 51.9 | 33.6 | 43.0 | 14.0 | 26.3 | 51.3 | 28.5 | 112.4 | 95.5 | 64.2 | 44.1 | 109.1 | 66.7 | 39.5 | 23.9 | 20.3 | 23.5 | 219.2 | 18.7 |
| **Feb-08** | 63.4 | 59.4 | 139.9 | 40.6 | 41.1 | 41.3 | 32.5 | 37.7 | 46.4 | 77.5 | 207.4 | 80.0 | 120.9 | 39.1 | 41.6 | 56.1 | 18.9 | 79.2 | 261.8 | 29.4 |
| **Mar-08** | 35.1 | 76.1 | 42.6 | 97.8 | 34.8 | 74.4 | 84.4 | 57.5 | 82.5 | 108.7 | 91.0 | 33.4 | 110.4 | 131.8 | 106.6 | 60.4 | 61.2 | 37.5 | 211.1 | 52.4 |
| **Apr-08** | 106.6 | 232.8 | 63.0 | 148.7 | 119.1 | 116.4 | 134.7 | 248.6 | 105.1 | 148.8 | 184.9 | 55.2 | 79.3 | 216.6 | 206.9 | 187.0 | 104.3 | 121.3 | 188.4 | 112.9 |
| **May-08** | 96.8 | 117.3 | 50.5 | 44.4 | 100.7 | 160.5 | 39.2 | 82.7 | 34.5 | 10.9 | 135.4 | 20.2 | 82.1 | 192.8 | 65.7 | 106.7 | 134.7 | 119.5 | 113.2 | 67.7 |
| **Jun-08** | 191.2 | 160.4 | 138.6 | 132.8 | 176.2 | 94.4 | 171.1 | 135.1 | 145.1 | 117.4 | 145.8 | 58.0 | 110.6 | 157.2 | 207.5 | 216.8 | 105.2 | 151.9 | 275.5 | 159.5 |
| **Jul-08** | 253.9 | 239.3 | 226.9 | 229.2 | 231.5 | 175.4 | 348.3 | 190.2 | 172.5 | 262.6 | 246.8 | 141.3 | 115.3 | 299.3 | 301.8 | 324.4 | 174.6 | 250.0 | 261.5 | 171.6 |
| **Aug-08** | 159.4 | 165.6 | 216.3 | 157.9 | 188.1 | 51.3 | 201.0 | 185.4 | 200.4 | 215.3 | 159.7 | 69.6 | 75.9 | 126.2 | 196.0 | 217.2 | 137.9 | 170.8 | 190.4 | 142.7 |
| **Sep-08** | 46.8 | 80.1 | 99.7 | 77.7 | 48.4 | 27.3 | 89.0 | 90.8 | 71.4 | 130.0 | 72.1 | 56.6 | 178.4 | 64.0 | 121.3 | 101.1 | 34.5 | 47.0 | 414.8 | 78.0 |
| **Oct-08** | 79.6 | 85.3 | 75.2 | 86.2 | 100.7 | 41.5 | 144.3 | 118.1 | 188.4 | 119.1 | 66.6 | 47.3 | 122.7 | 98.4 | 221.5 | 169.1 | 81.3 | 98.3 | 262.4 | 136.8 |
| **Nov-08** | 65.9 | 50.0 | 61.4 | 55.3 | 52.0 | 18.8 | 104.9 | 36.5 | 56.5 | 162.9 | 103.1 | 35.9 | 126.5 | 61.6 | 107.9 | 95.6 | 24.2 | 60.2 | 472.3 | 48.7 |
| **Dec-08** | 88.7 | 90.4 | 104.5 | 113.3 | 107.9 | 53.1 | 138.7 | 100.1 | 111.3 | 147.9 | 119.9 | 112.1 | 148.1 | 76.3 | 93.4 | 124.0 | 74.3 | 101.9 | 402.1 | 110.1 |
| **Jan-09** | 29.3 | 26.8 | 39.4 | 26.5 | 34.0 | 23.2 | 37.1 | 21.4 | 66.4 | 18.3 | 23.0 | 35.1 | 161.2 | 54.0 | 54.5 | 50.0 | 12.0 | 23.6 | 225.0 | 64.4 |
| **Feb-09** | 145.7 | 203.4 | 126.5 | 145.7 | 130.6 | 96.5 | 189.9 | 107.4 | 214.5 | 135.5 | 161.3 | 214.2 | 108.2 | 147.3 | 173.5 | 180.2 | 130.6 | 142.0 | 266.8 | 134.8 |
| **Mar-09** | 46.8 | 107.2 | 25.4 | 25.9 | 32.8 | 43.2 | 36.2 | 34.8 | 20.4 | 48.6 | 66.5 | 21.6 | 98.5 | 74.0 | 31.0 | 48.0 | 34.5 | 46.0 | 171.3 | 38.9 |
| **Apr-09** | 47.7 | 57.7 | 132.1 | 95.3 | 54.9 | 29.8 | 126.4 | 59.9 | 64.8 | 116.4 | 114.9 | 98.5 | 147.1 | 78.6 | 98.1 | 84.9 | 40.0 | 52.5 | 466.5 | 47.9 |
| **May-09** | 103.7 | 100.3 | 176.1 | 152.8 | 160.6 | 103.6 | 194.3 | 67.1 | 111.3 | 76.8 | 165.6 | 152.6 | 189.9 | 183.2 | 185.9 | 139.5 | 154.4 | 159.9 | 259.8 | 105.2 |
| **Jun-09** | 175.0 | 263.8 | 50.8 | 63.4 | 100.3 | 177.7 | 96.5 | 123.8 | 79.4 | 159.5 | 154.1 | 15.9 | 47.6 | 331.8 | 98.0 | 146.4 | 94.9 | 136.2 | 128.2 | 61.3 |
| **Jul-09** | 133.0 | 159.0 | 97.5 | 71.7 | 136.5 | 92.1 | 116.0 | 143.7 | 82.2 | 122.3 | 198.8 | 62.3 | 126.8 | 219.0 | 138.1 | 189.1 | 76.8 | 126.6 | 250.2 | 101.0 |
| **Aug-09** | 91.5 | 144.3 | 118.4 | 120.2 | 82.0 | 65.2 | 194.8 | 130.6 | 100.4 | 184.4 | 112.5 | 60.3 | 162.8 | 193.2 | 151.2 | 171.1 | 68.7 | 83.8 | 510.6 | 106.8 |
| **Sep-09** | 51.0 | 112.9 | 71.4 | 57.8 | 102.6 | 73.9 | 64.1 | 105.4 | 104.3 | 101.2 | 115.7 | 39.3 | 79.2 | 138.3 | 159.4 | 135.4 | 61.6 | 87.5 | 179.6 | 101.6 |
| **Oct-09** | 106.8 | 153.1 | 147.9 | 157.0 | 115.6 | 156.2 | 185.6 | 124.6 | 152.3 | 150.7 | 85.0 | 73.7 | 85.2 | 218.4 | 207.0 | 180.7 | 99.9 | 95.3 | 183.9 | 172.9 |
| **Nov-09** | 19.5 | 15.9 | 39.4 | 65.3 | 36.4 | 26.9 | 96.0 | 22.3 | 178.4 | 72.9 | 17.4 | 26.5 | 90.8 | 34.3 | 125.5 | 61.3 | 68.7 | 22.1 | 208.6 | 90.0 |
| **Dec-09** | 109.5 | 64.8 | 87.7 | 50.6 | 93.3 | 59.7 | 79.0 | 45.6 | 144.9 | 72.3 | 24.7 | 54.7 | 162.0 | 69.0 | 137.0 | 148.9 | 44.5 | 108.5 | 443.9 | 97.6 |
| **Jan-10** | 37.6 | 199.0 | 123.2 | 66.3 | 68.1 | 212.2 | 75.9 | 150.9 | 105.5 | 89.2 | 37.6 | 86.0 | 155.2 | 254.3 | 77.2 | 124.5 | 172.6 | 55.8 | 423.8 | 110.3 |
| **Feb-10** | 14.0 | 29.5 | 41.9 | 23.4 | 8.1 | 32.8 | 40.5 | 31.9 | 69.7 | 63.0 | 74.1 | 38.1 | 66.7 | 30.3 | 84.7 | 60.5 | 30.0 | 14.5 | 178.6 | 52.4 |
| **Mar-10** | 15.5 | 19.1 | 72.6 | 37.3 | 10.6 | 16.4 | 52.7 | 21.3 | 48.3 | 49.3 | 25.0 | 28.0 | 149.1 | 63.6 | 45.9 | 36.4 | 40.7 | 20.2 | 240.6 | 39.7 |
| **Apr-10** | 21.5 | 76.1 | 62.1 | 44.2 | 53.5 | 26.3 | 56.1 | 40.5 | 49.3 | 46.2 | 52.7 | 65.5 | 246.5 | 77.5 | 66.5 | 48.4 | 14.8 | 30.4 | 352.8 | 34.1 |
| **May-10** | 226.0 | 285.8 | 209.7 | 154.7 | 145.4 | 179.8 | 123.5 | 194.8 | 88.9 | 204.9 | 187.1 | 186.1 | 130.8 | 223.6 | 201.0 | 190.8 | 159.0 | 233.8 | 165.6 | 99.4 |
| **Jun-10** | 200.0 | 248.2 | 161.7 | 176.3 | 195.4 | 140.3 | 188.4 | 214.7 | 113.1 | 243.3 | 97.8 | 58.6 | 85.9 | 203.6 | 250.1 | 255.1 | 145.1 | 157.3 | 209.0 | 131.7 |
| **Jul-10** | 76.9 | 85.3 | 93.3 | 79.2 | 95.1 | 127.2 | 92.9 | 30.0 | 39.1 | 85.6 | 148.8 | 42.1 | 72.7 | 187.6 | 39.0 | 89.6 | 116.2 | 133.0 | 179.6 | 46.5 |
| **Aug-10** | 218.5 | 313.0 | 166.9 | 152.6 | 200.5 | 96.2 | 171.9 | 242.3 | 141.6 | 192.7 | 232.5 | 133.4 | 111.9 | 266.5 | 188.2 | 274.1 | 99.4 | 230.9 | 311.7 | 131.1 |
| **Sep-10** | 125.5 | 187.0 | 169.4 | 188.0 | 130.8 | 81.3 | 269.5 | 152.8 | 286.7 | 251.5 | 143.5 | 45.8 | 184.6 | 163.1 | 342.6 | 298.4 | 96.7 | 134.5 | 530.9 | 271.4 |
| **Oct-10** | 37.0 | 55.9 | 61.8 | 81.1 | 30.5 | 118.2 | 99.1 | 37.3 | 54.6 | 55.0 | 36.6 | 37.8 | 80.0 | 179.8 | 43.4 | 47.1 | 67.4 | 38.3 | 180.8 | 35.6 |
| **Nov-10** | 36.0 | 47.0 | 50.3 | 30.9 | 30.4 | 26.5 | 32.6 | 33.9 | 41.7 | 26.5 | 26.4 | 45.2 | 60.6 | 31.6 | 38.8 | 34.1 | 20.9 | 30.2 | 105.0 | 25.9 |
| **Dec-10** | 122.5 | 157.3 | 120.4 | 102.1 | 92.7 | 32.2 | 147.0 | 69.3 | 90.2 | 281.6 | 123.8 | 106.8 | 200.3 | 112.9 | 136.1 | 195.8 | 36.8 | 139.1 | 570.7 | 81.2 |
| **Jan-11** | 280.5 | 358.2 | 81.9 | 73.3 | 184.8 | 170.1 | 87.7 | 358.5 | 97.4 | 80.8 | 334.2 | 82.6 | 163.4 | 216.0 | 169.5 | 293.8 | 103.6 | 239.7 | 385.0 | 140.4 |
| **Feb-11** | 24.0 | 39.1 | 63.4 | 24.2 | 18.7 | 23.5 | 31.6 | 36.2 | 49.5 | 29.3 | 23.1 | 100.3 | 172.1 | 52.5 | 40.2 | 35.6 | 8.7 | 30.5 | 323.2 | 22.6 |
| **Mar-11** | 156.5 | 188.2 | 89.0 | 77.6 | 158.7 | 115.4 | 88.9 | 162.2 | 102.4 | 117.6 | 157.8 | 74.8 | 139.3 | 232.7 | 156.7 | 185.9 | 89.1 | 150.5 | 231.8 | 97.6 |
| **Apr-11** | 125.5 | 267.1 | 103.4 | 128.6 | 170.6 | 199.0 | 161.7 | 116.1 | 126.9 | 157.6 | 121.8 | 77.7 | 81.4 | 197.2 | 132.7 | 136.8 | 149.1 | 136.7 | 191.0 | 115.7 |
| **May-11** | 139.5 | 286.4 | 128.3 | 122.8 | 158.0 | 49.2 | 130.3 | 200.8 | 110.2 | 315.1 | 179.8 | 82.5 | 131.2 | 269.4 | 211.6 | 210.8 | 49.9 | 153.1 | 397.0 | 106.9 |
| **Jun-11** | 130.0 | 212.6 | 50.6 | 93.4 | 129.1 | 75.7 | 104.3 | 126.6 | 100.6 | 201.1 | 155.8 | 22.5 | 69.7 | 181.7 | 226.1 | 169.7 | 51.6 | 140.5 | 136.2 | 80.0 |
| **Jul-11** | 146.5 | 85.5 | 113.6 | 70.6 | 120.4 | 141.7 | 131.0 | 116.3 | 151.9 | 141.0 | 160.0 | 52.9 | 145.9 | 176.1 | 208.5 | 221.1 | 106.5 | 141.6 | 275.0 | 177.7 |
| **Aug-11** | 53.5 | 76.9 | 61.3 | 115.8 | 49.7 | 77.1 | 135.8 | 44.0 | 59.9 | 38.1 | 45.5 | 27.3 | 75.7 | 90.2 | 92.9 | 67.2 | 105.8 | 52.2 | 135.3 | 71.3 |
| **Sep-11** | 83.5 | 35.7 | 44.4 | 48.2 | 89.1 | 35.4 | 55.8 | 36.9 | 57.0 | 80.3 | 72.5 | 31.1 | 82.9 | 43.2 | 92.6 | 86.3 | 36.5 | 90.3 | 149.8 | 70.5 |
| **Oct-11** | 106.1 | 222.8 | 142.0 | 110.0 | 121.9 | 85.0 | 141.3 | 143.2 | 177.6 | 217.7 | 98.5 | 126.4 | 130.8 | 144.5 | 227.5 | 161.3 | 100.1 | 106.1 | 301.9 | 149.6 |
| **Nov-11** | 29.0 | 41.1 | 127.9 | 71.8 | 58.4 | 44.6 | 108.2 | 31.1 | 144.7 | 121.3 | 19.5 | 101.2 | 127.9 | 62.9 | 125.6 | 73.8 | 61.9 | 45.9 | 337.7 | 89.5 |
| **Dec-11** | 186.0 | 258.7 | 103.3 | 111.3 | 179.6 | 92.2 | 105.5 | 263.2 | 142.4 | 305.8 | 228.6 | 70.2 | 28.4 | 145.9 | 262.3 | 239.3 | 105.8 | 180.7 | 221.6 | 153.7 |
| **Jan-12** | 75.5 | 93.6 | 58.4 | 72.6 | 61.5 | 128.8 | 109.5 | 108.0 | 100.8 | 44.5 | 72.1 | 92.9 | 119.4 | 127.4 | 85.2 | 116.8 | 87.9 | 63.1 | 274.1 | 121.0 |
| **Feb-12** | 111.5 | 93.7 | 81.0 | 60.2 | 122.4 | 73.0 | 74.7 | 112.3 | 101.2 | 109.3 | 65.7 | 107.3 | 99.1 | 120.8 | 129.2 | 131.6 | 52.4 | 105.2 | 228.9 | 84.5 |
| **Mar-12** | 186.4 | 166.7 | 87.0 | 123.5 | 126.4 | 185.0 | 152.8 | 98.1 | 76.2 | 91.5 | 269.6 | 62.8 | 128.5 | 207.9 | 129.6 | 141.1 | 211.5 | 188.3 | 175.2 | 64.6 |
| **Apr-12** | 46.5 | 73.2 | 64.5 | 40.2 | 22.7 | 84.8 | 54.6 | 70.0 | 30.8 | 63.7 | 74.8 | 38.6 | 57.5 | 220.2 | 59.2 | 64.4 | 61.5 | 32.9 | 162.0 | 31.5 |
| **May-12** | 134.5 | 147.4 | 54.3 | 81.0 | 99.5 | 74.3 | 108.1 | 115.8 | 78.6 | 72.7 | 96.4 | 43.3 | 101.3 | 135.4 | 135.4 | 150.3 | 66.3 | 107.9 | 179.5 | 84.5 |
| **Jun-12** | 60.5 | 86.5 | 106.6 | 127.9 | 106.8 | 44.7 | 163.9 | 56.7 | 98.5 | 234.4 | 61.7 | 50.5 | 120.6 | 120.9 | 154.5 | 134.7 | 82.8 | 68.0 | 255.6 | 109.9 |
| **Jul-12** | 229.5 | 238.8 | 101.7 | 84.9 | 170.6 | 238.5 | 105.3 | 145.7 | 129.7 | 198.8 | 193.2 | 113.3 | 81.9 | 262.5 | 193.3 | 229.6 | 169.6 | 158.1 | 241.9 | 82.0 |
| **Aug-12** | 125.5 | 141.0 | 174.8 | 93.7 | 133.7 | 89.1 | 108.6 | 114.7 | 87.8 | 195.0 | 150.0 | 163.6 | 66.7 | 160.1 | 165.0 | 168.0 | 93.6 | 138.2 | 147.3 | 100.6 |
| **Sep-12** | 101.0 | 112.6 | 82.9 | 65.6 | 97.1 | 35.8 | 113.4 | 117.3 | 91.1 | 216.9 | 144.7 | 62.6 | 194.9 | 106.8 | 136.3 | 146.7 | 59.9 | 109.0 | 390.0 | 95.2 |
| **Oct-12** | 87.5 | 78.5 | 160.2 | 74.9 | 69.9 | 22.0 | 119.9 | 64.0 | 91.5 | 142.4 | 50.1 | 122.3 | 186.9 | 106.8 | 134.6 | 142.0 | 50.3 | 66.7 | 514.7 | 94.2 |
| **Nov-12** | 45.0 | 31.2 | 75.1 | 25.0 | 26.8 | 61.2 | 38.1 | 16.0 | 53.1 | 45.3 | 50.1 | 77.0 | 98.7 | 76.5 | 70.5 | 41.8 | 27.5 | 44.7 | 135.4 | 59.1 |
| **Dec-12** | 63.5 | 96.4 | 66.4 | 75.8 | 86.1 | 47.6 | 124.7 | 89.6 | 142.4 | 100.7 | 124.6 | 51.7 | 134.8 | 101.0 | 115.5 | 137.1 | 45.8 | 78.4 | 289.4 | 88.1 |
| **Jan-13** | 5.0 | 11.0 | 139.2 | 89.7 | 11.3 | 30.8 | 124.4 | 10.9 | 77.0 | 143.8 | 12.5 | 103.9 | 177.0 | 54.5 | 75.7 | 24.8 | 20.9 | 6.1 | 299.4 | 57.1 |
| **Feb-13** | 13.5 | 42.2 | 35.2 | 69.8 | 29.6 | 28.2 | 72.8 | 16.9 | 60.1 | 32.2 | 17.8 | 42.8 | 45.4 | 40.6 | 49.2 | 35.7 | 40.7 | 31.8 | 67.2 | 43.9 |
| **Mar-13** | 33.1 | 25.7 | 80.5 | 74.1 | 48.5 | 38.9 | 67.2 | 23.1 | 83.9 | 87.7 | 21.8 | 54.5 | 69.9 | 47.8 | 66.6 | 44.8 | 86.5 | 47.4 | 148.8 | 52.6 |
| **Apr-13** | 96.5 | 218.0 | 103.8 | 65.4 | 116.2 | 64.3 | 88.8 | 135.8 | 127.0 | 185.0 | 87.5 | 79.9 | 123.7 | 142.9 | 151.2 | 172.4 | 67.8 | 104.9 | 197.6 | 61.4 |
| **May-13** | 208.0 | 135.4 | 115.4 | 134.0 | 239.7 | 68.5 | 141.4 | 129.0 | 83.1 | 161.7 | 147.0 | 93.2 | 147.3 | 161.2 | 174.7 | 196.9 | 93.6 | 231.8 | 182.4 | 97.8 |
| **Jun-13** | 128.5 | 150.6 | 214.2 | 246.0 | 151.5 | 136.6 | 241.6 | 139.9 | 117.5 | 209.6 | 133.6 | 179.1 | 166.2 | 161.8 | 175.0 | 180.9 | 148.7 | 177.4 | 242.4 | 138.1 |
| **Jul-13** | 35.0 | 86.3 | 85.5 | 105.2 | 48.8 | 154.9 | 124.1 | 43.2 | 60.7 | 66.2 | 100.3 | 27.8 | 165.1 | 160.2 | 93.6 | 53.4 | 94.5 | 51.7 | 273.3 | 72.0 |
| **Aug-13** | 140.7 | 97.7 | 87.2 | 75.5 | 125.5 | 163.4 | 85.0 | 95.4 | 80.1 | 166.1 | 167.2 | 32.6 | 60.9 | 260.1 | 136.5 | 126.6 | 89.9 | 151.5 | 243.4 | 66.6 |
| **Sep-13** | 154.4 | 181.3 | 158.4 | 110.9 | 143.3 | 113.7 | 187.7 | 140.1 | 114.5 | 179.4 | 159.3 | 62.1 | 138.2 | 265.7 | 208.2 | 209.0 | 120.9 | 162.4 | 327.4 | 129.6 |
| **Oct-13** | 28.2 | 28.6 | 192.8 | 146.9 | 37.9 | 41.5 | 224.1 | 51.3 | 149.1 | 215.6 | 23.6 | 92.6 | 246.3 | 69.7 | 195.7 | 119.7 | 110.4 | 28.0 | 510.2 | 174.4 |
| **Nov-13** | 79.0 | 80.1 | 65.0 | 52.8 | 81.0 | 201.2 | 49.5 | 104.9 | 61.7 | 78.5 | 63.1 | 49.1 | 66.5 | 107.8 | 132.3 | 113.8 | 150.7 | 75.2 | 151.7 | 87.0 |
| **Dec-13** | 142.5 | 174.1 | 120.1 | 37.0 | 114.0 | 57.1 | 39.0 | 113.8 | 67.5 | 94.5 | 125.2 | 103.9 | 151.3 | 119.0 | 135.1 | 141.6 | 41.1 | 111.4 | 315.5 | 56.5 |
| **Jan-14** | 92.5 | 84.0 | 93.3 | 106.1 | 50.4 | 47.5 | 125.0 | 36.6 | 97.8 | 111.0 | 33.6 | 62.9 | 122.5 | 114.8 | 167.6 | 61.2 | 56.5 | 44.4 | 341.1 | 77.6 |
| **Feb-14** | 26.0 | 68.1 | 59.6 | 22.7 | 16.0 | 48.8 | 22.1 | 17.8 | 26.3 | 24.2 | 78.8 | 42.0 | 76.1 | 29.9 | 20.9 | 29.1 | 50.2 | 20.9 | 142.1 | 24.7 |
| **Mar-14** | 33.5 | 63.4 | 101.9 | 55.6 | 33.7 | 64.6 | 78.2 | 39.0 | 23.8 | 88.2 | 61.5 | 54.2 | 58.7 | 58.3 | 21.7 | 38.9 | 49.2 | 26.1 | 121.1 | 34.8 |
| **Apr-14** | 108.5 | 234.3 | 245.3 | 118.5 | 122.5 | 213.5 | 116.3 | 170.1 | 152.1 | 264.8 | 93.3 | 128.6 | 136.4 | 207.4 | 236.8 | 187.9 | 200.1 | 97.1 | 304.4 | 119.6 |
| **May-14** | 42.5 | 67.0 | 115.8 | 72.2 | 87.3 | 30.6 | 117.9 | 54.1 | 114.2 | 109.8 | 54.1 | 57.6 | 249.0 | 68.9 | 113.6 | 144.0 | 46.0 | 58.5 | 341.5 | 87.5 |
| **Jun-14** | 266.5 | 212.9 | 145.4 | 77.2 | 195.5 | 129.7 | 106.5 | 129.8 | 90.6 | 238.5 | 265.1 | 95.1 | 77.6 | 226.0 | 167.0 | 186.6 | 60.4 | 184.5 | 448.8 | 88.4 |
| **Jul-14** | 188.0 | 63.3 | 49.1 | 82.4 | 93.8 | 106.9 | 97.5 | 54.0 | 29.7 | 106.8 | 319.9 | 26.1 | 95.2 | 113.0 | 86.5 | 98.9 | 83.2 | 124.3 | 199.8 | 47.0 |
| **Aug-14** | 145.0 | 87.3 | 77.3 | 78.0 | 138.9 | 101.0 | 144.6 | 84.3 | 118.6 | 68.2 | 233.3 | 59.6 | 96.1 | 247.2 | 123.4 | 148.0 | 82.0 | 164.1 | 195.7 | 128.2 |
| **Sep-14** | 156.0 | 183.2 | 65.4 | 102.2 | 154.6 | 118.4 | 144.8 | 85.4 | 102.6 | 113.0 | 118.7 | 30.8 | 76.6 | 321.4 | 161.3 | 147.2 | 89.3 | 165.1 | 219.6 | 94.9 |
| **Oct-14** | 109.5 | 78.3 | 94.4 | 49.3 | 101.1 | 54.0 | 94.3 | 64.4 | 83.5 | 92.2 | 93.7 | 51.9 | 115.4 | 80.2 | 139.7 | 124.8 | 52.3 | 127.7 | 258.7 | 132.1 |
| **Nov-14** | 76.5 | 49.4 | 124.0 | 42.4 | 70.4 | 39.9 | 71.2 | 50.1 | 85.3 | 73.5 | 60.4 | 80.5 | 138.4 | 60.3 | 138.0 | 118.4 | 62.1 | 74.2 | 491.4 | 99.3 |
| **Dec-14** | 98.0 | 164.3 | 58.7 | 107.0 | 77.8 | 78.7 | 114.7 | 70.7 | 138.7 | 133.5 | 149.0 | 38.6 | 79.5 | 166.9 | 119.0 | 104.6 | 31.0 | 104.6 | 306.0 | 77.4 |
| **Jan-15** | 6.0 | 13.9 | 17.0 | 3.6 | 12.3 | 25.5 | 18.0 | 29.1 | 5.1 | 15.9 | 15.1 | 35.7 | 104.5 | 43.1 | 16.4 | 29.1 | 13.1 | 11.4 | 123.0 | 5.8 |
| **Feb-15** | 38.5 | 62.5 | 43.6 | 20.8 | 30.7 | 34.4 | 15.3 | 75.3 | 66.6 | 44.4 | 48.7 | 41.4 | 113.6 | 26.6 | 84.3 | 59.7 | 27.3 | 49.3 | 177.0 | 59.1 |
| **Mar-15** | 92.0 | 63.7 | 68.2 | 65.7 | 71.9 | 84.7 | 59.7 | 62.8 | 92.7 | 115.6 | 64.5 | 67.6 | 86.8 | 123.8 | 105.2 | 75.2 | 38.1 | 110.4 | 252.4 | 63.1 |
| **Apr-15** | 104.4 | 106.2 | 115.0 | 139.7 | 137.2 | 91.2 | 192.4 | 113.9 | 196.5 | 142.1 | 58.1 | 93.2 | 158.7 | 124.6 | 272.2 | 199.2 | 72.3 | 91.2 | 392.0 | 205.6 |
| **May-15** | 150.5 | 164.5 | 97.9 | 143.0 | 147.7 | 61.1 | 220.3 | 103.4 | 151.1 | 82.5 | 174.5 | 18.8 | 172.2 | 184.6 | 213.4 | 174.7 | 52.8 | 166.4 | 376.6 | 123.0 |
| **Jun-15** | 64.5 | 87.8 | 122.2 | 111.4 | 86.4 | 77.1 | 147.8 | 87.3 | 229.7 | 133.7 | 69.3 | 98.2 | 192.6 | 199.5 | 328.8 | 143.1 | 105.1 | 74.8 | 469.9 | 228.9 |
| **Jul-15** | 111.0 | 133.3 | 82.4 | 102.3 | 163.5 | 62.5 | 125.8 | 117.0 | 79.2 | 137.1 | 120.5 | 25.6 | 96.8 | 143.0 | 133.1 | 157.6 | 62.1 | 130.4 | 289.6 | 103.7 |
| **Aug-15** | 124.5 | 121.8 | 87.5 | 93.8 | 160.8 | 75.6 | 158.2 | 129.9 | 114.2 | 170.6 | 149.0 | 59.4 | 120.4 | 115.0 | 234.4 | 198.4 | 86.8 | 176.9 | 297.6 | 121.6 |
| **Sep-15** | 85.0 | 148.0 | 96.4 | 70.9 | 99.0 | 200.2 | 100.0 | 99.0 | 86.0 | 110.0 | 108.4 | 42.2 | 82.8 | 128.7 | 148.9 | 154.3 | 182.2 | 97.5 | 190.2 | 87.4 |
| **Oct-15** | 40.0 | 30.2 | 53.6 | 44.0 | 51.9 | 38.5 | 111.8 | 18.9 | 117.5 | 70.1 | 51.9 | 37.9 | 159.4 | 59.1 | 95.4 | 83.0 | 40.5 | 41.4 | 367.3 | 80.3 |
| **Nov-15** | 96.5 | 84.5 | 65.0 | 44.9 | 97.3 | 97.8 | 75.5 | 91.0 | 92.1 | 41.6 | 57.8 | 41.4 | 115.6 | 178.3 | 122.0 | 135.3 | 30.2 | 87.3 | 239.0 | 85.0 |
| **Dec-15** | 36.0 | 29.2 | 56.9 | 18.2 | 24.0 | 30.5 | 35.1 | 28.2 | 61.5 | 33.5 | 31.0 | 65.4 | 87.9 | 29.9 | 47.8 | 52.6 | 38.3 | 50.0 | 284.7 | 32.4 |
| **Jan-16** | 132.0 | 137.3 | 147.4 | 71.9 | 133.2 | 97.7 | 88.0 | 119.6 | 87.9 | 146.6 | 128.1 | 111.3 | 160.9 | 144.4 | 86.8 | 148.1 | 55.1 | 114.6 | 388.2 | 81.5 |
| **Feb-16** | 224.5 | 133.8 | 47.1 | 34.8 | 96.4 | 17.4 | 63.9 | 108.0 | 60.7 | 110.1 | 152.1 | 27.3 | 182.8 | 142.5 | 109.3 | 128.5 | 16.0 | 164.1 | 281.1 | 40.1 |
| **Mar-16** | 62.5 | 133.3 | 61.0 | 20.6 | 34.6 | 69.1 | 21.7 | 96.4 | 76.3 | 126.0 | 62.5 | 53.7 | 150.4 | 61.5 | 77.8 | 102.5 | 24.4 | 51.2 | 297.3 | 60.2 |
| **Apr-16** | 84.5 | 123.7 | 34.1 | 49.0 | 51.6 | 38.1 | 60.3 | 43.2 | 49.5 | 56.9 | 79.2 | 36.4 | 100.4 | 85.6 | 67.4 | 79.0 | 34.2 | 68.4 | 239.5 | 48.9 |
| **May-16** | 93.9 | 110.8 | 176.0 | 217.8 | 136.2 | 46.6 | 312.3 | 90.4 | 238.7 | 218.2 | 115.6 | 90.1 | 267.8 | 149.7 | 263.0 | 185.4 | 116.8 | 113.5 | 516.6 | 161.8 |
| **Jun-16** | 82.5 | 245.5 | 53.2 | 121.3 | 141.3 | 65.0 | 154.4 | 130.0 | 96.1 | 182.6 | 144.2 | 32.7 | 104.3 | 230.5 | 160.2 | 193.8 | 62.6 | 125.0 | 389.2 | 93.6 |
| **Jul-16** | 138.0 | 138.5 | 118.8 | 77.8 | 156.9 | 77.8 | 126.8 | 122.6 | 120.7 | 168.3 | 142.4 | 70.0 | 187.5 | 197.2 | 171.4 | 205.2 | 46.0 | 169.4 | 406.9 | 166.9 |
| **Aug-16** | 162.5 | 97.8 | 64.0 | 117.7 | 93.7 | 157.7 | 120.0 | 76.4 | 122.8 | 68.1 | 132.8 | 47.7 | 65.0 | 149.6 | 172.3 | 118.7 | 94.2 | 150.2 | 144.6 | 106.4 |
| **Sep-16** | 152.5 | 131.0 | 81.3 | 119.2 | 173.5 | 129.6 | 194.2 | 119.0 | 107.0 | 129.7 | 109.9 | 19.7 | 62.0 | 182.2 | 181.8 | 166.1 | 79.3 | 170.8 | 229.8 | 86.5 |
| **Oct-16** | 135.0 | 99.1 | 93.5 | 111.4 | 81.5 | 65.2 | 124.5 |  | 102.9 | 127.0 | 84.4 | 92.8 | 184.2 | 124.5 | 153.0 | 133.6 | 61.8 | 105.8 | 298.9 | 102.7 |
| **Nov-16** | 130.0 | 117.8 | 138.7 | 197.1 | 83.6 | 33.2 | 264.0 | 112.4 | 176.9 | 221.1 | 90.6 | 132.5 | 177.2 | 192.9 | 206.8 | 145.6 | 121.1 | 110.4 | 388.1 | 125.4 |
| **Dec-16** | 32.5 | 68.2 | 76.9 | 85.8 | 40.5 | 31.8 | 90.4 | 53.0 | 80.8 | 99.1 | 24.4 | 63.2 | 107.7 | 68.2 | 75.2 | 58.1 | 42.1 | 28.4 | 439.2 | 54.4 |
| **Jan-17** | 35.0 | 43.9 | 104.4 | 73.4 | 40.8 | 19.9 | 139.9 | 42.0 | 122.9 | 144.9 | 46.2 | 90.7 | 222.8 | 23.1 | 160.3 | 97.1 | 50.4 | 35.1 | 490.4 | 74.4 |
| **Feb-17** | 149.0 | 106.7 | 44.5 | 93.8 | 74.1 | 186.3 | 137.4 | 171.9 | 103.9 | 61.6 | 97.5 | 38.5 | 80.4 | 99.6 | 137.8 | 138.1 | 113.7 | 83.6 | 187.2 | 94.3 |
| **Mar-17** | 401.0 | 269.4 | 100.6 | 109.7 | 278.7 | 99.2 | 112.4 | 254.2 | 146.5 | 121.0 | 195.9 | 100.5 | 53.0 | 236.2 | 143.8 | 228.8 | 64.5 | 309.8 | 175.9 | 97.0 |
| **Apr-17** | 314.5 | 325.1 | 176.0 | 191.3 | 186.7 | 233.4 | 243.9 | 290.4 | 219.3 | 211.1 | 175.5 | 122.3 | 99.1 | 295.2 | 336.4 | 314.7 | 184.8 | 280.3 | 240.0 | 197.5 |
| **May-17** | 117.0 | 148.8 | 73.3 | 80.8 | 127.9 | 112.9 | 84.4 | 191.8 | 115.9 | 197.2 | 101.8 | 43.2 | 84.7 | 217.1 | 168.6 | 179.3 | 72.1 | 138.7 | 218.5 | 131.7 |
| **Jun-17** | 150.6 | 93.4 | 57.2 | 48.9 | 51.4 | 78.9 | 18.5 | 62.0 | 33.0 | 65.5 | 127.9 | 45.5 | 96.6 | 132.9 | 89.0 | 90.8 | 46.8 | 81.0 | 233.7 | 44.2 |
| **Jul-17** | 168.0 | 199.0 | 159.3 | 212.2 | 188.7 | 162.6 | 189.7 | 151.3 | 161.7 | 124.2 | 171.3 | 171.7 | 148.0 | 234.5 | 250.2 | 191.9 | 78.7 | 188.1 | 224.0 | 145.8 |
| **Aug-17** | 100.5 | 154.8 | 126.1 | 154.0 | 122.1 | 56.3 | 183.7 | 197.8 | 106.7 | 175.9 | 134.7 | 68.7 | 107.4 | 131.5 | 250.1 | 184.4 | 95.9 | 115.2 | 299.9 | 122.5 |
| **Sep-17** | 124.0 | 142.5 | 172.0 | 106.0 | 120.2 | 76.7 | 146.7 | 130.0 | 148.5 | 171.5 | 127.2 | 80.5 | 144.3 | 181.7 | 265.3 | 238.3 | 72.9 | 171.8 | 335.0 | 144.0 |
| **Oct-17** | 77.0 | 132.0 | 123.6 | 47.9 | 99.8 | 94.2 | 66.1 | 69.9 | 86.7 | 122.3 | 55.2 | 76.9 | 62.5 | 81.1 | 89.9 | 118.7 | 58.0 | 78.4 | 173.1 | 94.4 |
| **Nov-17** | 59.0 | 53.6 | 47.5 | 16.1 | 44.0 | 31.0 | 25.3 | 93.5 | 33.3 | 48.8 | 72.4 | 29.1 | 69.9 | 104.9 | 55.4 | 93.4 | 9.1 | 43.5 | 177.3 | 59.1 |
| **Dec-17** | 25.0 | 32.6 | 46.3 | 46.2 | 17.2 | 78.1 | 59.7 | 82.8 | 40.1 | 32.8 | 28.9 | 62.7 | 126.6 | 61.7 | 18.0 | 28.4 | 58.6 | 16.7 | 191.2 | 58.8 |

*Supplementary Table S6: Table of the New Zealand District Health Board wards and the names of their contributing New Zealand Territorial Authorities.*

| **Northland DHB** | **Lakes DHB** | **Midcentral DHB** | **Canterbury DHB** |
| --- | --- | --- | --- |
| 001 Far North district | 021 Taupo district | 039 Manawatu district | 054 Kaikoura district |
| 002 Whangarei district | 024 Rotorua district | 040 Palmerston North city | 058 Hurunui district |
| 003 Kaipara district | **Bay of Plenty DHB** | 041 Tararua district | 059 Waimakariri district |
| **Waitemata DHB** | 022 Western Bay of Plenty district | 042 Horowhenua district | 060 Christchurch city |
| 004 Rodney district (pre-2010 Territorial Authority boundary) | 023 Tauranga city | **Hutt DHB** | 062 Selwyn district |
| 005 North Shore city (pre-2010 Territorial Authority boundary) | 025 Whakatane district | 045 Upper Hutt city | 063 Ashburton district |
| 006 Waitakere city (pre-2010 Territorial Authority boundary) | 026 Kawerau district | 046 Lower Hutt city | **South Canterbury DHB** |
| **Auckland DHB** | 027 Opotiki district | **Capital and Coast DHB** | 064 Timaru district |
| 007 Auckland city (pre-2010 Territorial Authority boundary) | **Tairawhiti DHB** | 043 Kapiti Coast district | 065 Mackenzie district |
| **Counties Manukau DHB** | 028 Gisborne district | 044 Porirua city | 066 Waimate district |
| 008 Manukau city (pre-2010 Territorial Authority boundary) | **Taranaki DHB** | 047 Wellington city | **Southern DHB** |
| 009 Papakura district (pre-2010 Territorial Authority boundary) | 033 New Plymouth district | **Wairarapa DHB** | 068 Waitaki district |
| 010 Franklin district (pre-2010 Territorial Authority boundary) | 034 Stratford district | 048 Masterton district | 069 Central Otago district |
| **Waikato DHB** | 035 South Taranaki district | 049 Carterton district | 070 Queenstown-Lakes district |
| 011 Thames-Coromandel district | **Hawke’s Bay DHB** | 050 South Wairarapa district | 071 Dunedin city |
| 012 Hauraki district | 029 Wairoa district | **Nelson-Marlborough DHB** | 072 Clutha district |
| 013 Waikato district | 030 Hastings district | 051 Tasman district | 073 Southland district |
| 015 Matamata-Piako district | 031 Napier city | 052 Nelson city | 074 Gore district |
| 016 Hamilton city | 032 Central Hawke’s Bay district | 053 Marlborough district | 075 Invercargill city |
| 017 Waipa district | 067 Chatham Islands territory | **West Coast DHB** | **Not specified** |
| 018 Otorohanga district | **Whanganui DHB** | 055 Buller district | 999 Area outside territorial authority |
| 019 South Waikato district | 037 Whanganui district | 056 Grey district | |
| 020 Waitomo district | 038 Rangitikei district | 057 Westland district | |
| 036 Ruapehu district | | |  |

*The District Health Board (DHB) areas are aggregated from Territorial Authority (TA) boundaries. In a few cases, a TA boundary straddles DHB boundaries. These TAs have been assigned to the DHB containing the greatest proportion of their population. The TAs affected are Bay of Plenty district, Ruapehu district, and Kapiti Coast district (StatsNZ, 2021a)*


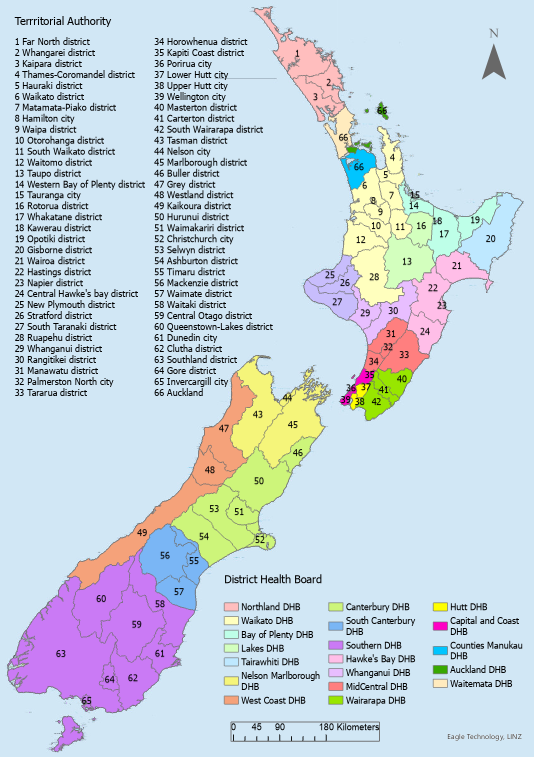


*Supplementary Figure S3: Map showing the aggregation of Territorial Authorities into District Health Boards. Map lines delineate study areas and do not necessarily depict accepted national boundaries.*

Supplementary Material S3: Model of all serovars supplementary tables

*Supplementary Table S7: Summary of average monthly incidence (number of cases /100 000 people) of reported human leptospirosis cases included in the analysis in each New Zealand District Health Board during the period 1 January 1999 to 31 December 2017.*

| District Health Board | Median | Mean | Standard deviation | Interquartile Range | Minimum | Maximum |
| --- | --- | --- | --- | --- | --- | --- |
| Auckland | 0.00 | 0.01 | 0.04 | 0.00 | 0.00 | 0.26 |
| Bay of Plenty | 0.00 | 0.16 | 0.31 | 0.42 | 0.00 | 2.14 |
| Canterbury | 0.00 | 0.10 | 0.16 | 0.20 | 0.00 | 0.81 |
| Capital and Coast | 0.00 | 0.02 | 0.08 | 0.00 | 0.00 | 0.40 |
| Counties Manukau | 0.00 | 0.03 | 0.08 | 0.00 | 0.00 | 0.43 |
| Hawke's Bay | 0.59 | 0.57 | 0.74 | 0.67 | 0.00 | 3.37 |
| Hutt Valley | 0.00 | 0.02 | 0.14 | 0.00 | 0.00 | 1.42 |
| Lakes | 0.00 | 0.12 | 0.35 | 0.00 | 0.00 | 1.97 |
| MidCentral | 0.00 | 0.35 | 0.50 | 0.61 | 0.00 | 2.45 |
| Nelson Marlborough | 0.00 | 0.23 | 0.52 | 0.00 | 0.00 | 3.14 |
| Northland | 0.00 | 0.34 | 0.53 | 0.66 | 0.00 | 2.83 |
| South Canterbury | 0.00 | 0.31 | 0.81 | 0.00 | 0.00 | 3.70 |
| Southern | 0.00 | 0.15 | 0.27 | 0.33 | 0.00 | 2.06 |
| Tairawhiti | 0.00 | 0.58 | 1.49 | 0.00 | 0.00 | 10.87 |
| Taranaki | 0.00 | 0.29 | 0.52 | 0.84 | 0.00 | 2.83 |
| Waikato | 0.28 | 0.35 | 0.40 | 0.56 | 0.00 | 2.91 |
| Wairarapa | 0.00 | 0.32 | 0.91 | 0.00 | 0.00 | 5.06 |
| Waitemata | 0.00 | 0.03 | 0.08 | 0.00 | 0.00 | 0.50 |
| West Coast | 0.00 | 0.64 | 1.47 | 0.00 | 0.00 | 6.37 |
| Whanganui | 0.00 | 0.47 | 0.96 | 0.00 | 0.00 | 4.76 |

*Supplementary Table 8: Summary statistics of the monthly human population in each New Zealand District Health Board the period 1 January 1999 to 31 December 2017.*

| District health board | Median | Mean | Standard deviation | Interquartile range | Minimum | Maximum |
| --- | --- | --- | --- | --- | --- | --- |
| Auckland | 436000 | 435384 | 31282 | 46300 | 381100 | 488000 |
| Bay of Plenty | 205900 | 205563 | 17064 | 24400 | 178800 | 241600 |
| Canterbury | 497400 | 489111 | 32793 | 50300 | 435700 | 552700 |
| Capital and Coast | 282500 | 281153 | 17565 | 27300 | 251700 | 311500 |
| Counties Manukau | 467400 | 463153 | 49144 | 75000 | 380900 | 553800 |
| Hawke's Bay | 153400 | 154863 | 6479 | 8700 | 147200 | 169600 |
| Hutt Valley | 141200 | 141868 | 4012 | 4200 | 136700 | 152100 |
| Lakes | 101500 | 102640 | 3058 | 2325 | 99400 | 111400 |
| MidCentral | 164500 | 166458 | 5227 | 6700 | 160300 | 179000 |
| Nelson Marlborough | 135800 | 136537 | 8386 | 12600 | 123400 | 152900 |
| Northland | 156300 | 157784 | 10940 | 16800 | 143800 | 181200 |
| South Canterbury | 55500 | 56158 | 1955 | 3100 | 53800 | 60500 |
| Southern | 297000 | 299663 | 13257 | 17300 | 282500 | 330400 |
| Tairawhiti | 46300 | 46637 | 1055 | 1200 | 45500 | 49200 |
| Taranaki | 108400 | 110526 | 4383 | 6800 | 105900 | 120100 |
| Waikato | 357500 | 360590 | 24920 | 39900 | 327900 | 412200 |
| Wairarapa | 40200 | 41063 | 2054 | 3000 | 39000 | 45700 |
| Waitemata | 517400 | 515890 | 48116 | 76800 | 435700 | 602900 |
| West Coast | 32400 | 32258 | 695 | 1400 | 31000 | 33100 |
| Whanganui | 64000 | 64289 | 1416 | 2100 | 62300 | 67500 |

*Supplementary Table 9: Summary statistics of monthly animal density (animals per km^2^) in each New Zealand District Health Board in the period 1 January 1999 to 31 December 2017 in New Zealand.*

| District health board | Median | Mean | Standard deviation | Interquartile range | Minimum | Maximum |
| --- | --- | --- | --- | --- | --- | --- |
| Auckland | 58.94 | 66.06 | 9.43 | 18.05 | 58.94 | 81.50 |
| Bay of Plenty | 53.82 | 56.04 | 7.07 | 15.26 | 48.40 | 66.51 |
| Canterbury | 217.28 | 208.02 | 24.49 | 49.78 | 166.53 | 233.14 |
| Capital and Coast | 93.59 | 88.04 | 15.08 | 21.80 | 54.03 | 106.92 |
| Counties Manukau | 189.30 | 197.98 | 11.48 | 21.97 | 189.30 | 216.77 |
| Hawke's Bay | 318.52 | 316.45 | 30.65 | 56.09 | 257.44 | 353.93 |
| Hutt Valley | 25.40 | 26.98 | 10.19 | 16.15 | 5.92 | 40.36 |
| Lakes | 128.42 | 119.24 | 13.16 | 27.17 | 100.14 | 134.98 |
| MidCentral | 410.85 | 393.27 | 39.24 | 81.42 | 330.18 | 431.43 |
| Nelson Marlborough | 56.16 | 58.15 | 8.58 | 17.38 | 45.81 | 70.48 |
| Northland | 110.30 | 106.06 | 9.47 | 18.04 | 88.33 | 115.08 |
| South Canterbury | 186.59 | 176.24 | 22.69 | 44.02 | 134.92 | 197.82 |
| Southern | 201.93 | 194.50 | 18.34 | 35.88 | 161.34 | 212.75 |
| Tairawhiti | 242.76 | 233.95 | 17.41 | 31.60 | 200.63 | 256.61 |
| Taranaki | 178.06 | 177.06 | 15.95 | 33.54 | 155.07 | 196.98 |
| Waikato | 230.91 | 222.02 | 14.20 | 26.08 | 195.09 | 235.93 |
| Wairarapa | 325.11 | 318.95 | 18.73 | 34.11 | 281.63 | 338.28 |
| Waitemata | 124.43 | 130.18 | 7.62 | 14.58 | 124.43 | 142.65 |
| West Coast | 10.88 | 11.36 | 0.71 | 1.38 | 10.75 | 12.50 |
| Whanganui | 394.69 | 375.24 | 30.92 | 54.84 | 314.11 | 405.02 |

*Supplementary Table 10: Summary statistics for the examined total monthly rainfall (cm) (Total Rainfall) in each New Zealand District Health Board over the period 1 January 1999 to 31 December 2017*

| District Health Board | Median | Mean | Standard Deviation | Interquartile Range | Minimum | Maximum |
| --- | --- | --- | --- | --- | --- | --- |
| Auckland | 9.32 | 10.45 | 6.52 | 8.04 | 0.50 | 40.10 |
| Bay of Plenty | 10.67 | 12.25 | 7.13 | 8.31 | 1.10 | 43.54 |
| Canterbury | 8.68 | 9.54 | 4.66 | 6.19 | 1.70 | 24.53 |
| Capital and Coast | 7.96 | 8.89 | 5.08 | 6.45 | 0.36 | 35.38 |
| Counties Manukau | 9.09 | 9.57 | 5.24 | 7.62 | 0.66 | 27.87 |
| Hawke's Bay | 7.34 | 8.47 | 5.13 | 7.11 | 0.60 | 23.85 |
| Hutt Valley | 10.54 | 11.19 | 6.38 | 7.99 | 1.44 | 42.32 |
| Lakes | 9.04 | 9.78 | 5.89 | 7.73 | 0.28 | 35.85 |
| MidCentral | 9.30 | 9.82 | 4.94 | 5.81 | 0.51 | 34.67 |
| Nelson Marlborough | 11.12 | 11.95 | 6.46 | 8.79 | 0.72 | 31.51 |
| Northland | 10.10 | 11.13 | 6.53 | 8.55 | 0.72 | 37.43 |
| South Canterbury | 6.02 | 6.78 | 3.74 | 4.38 | 1.11 | 21.42 |
| Southern | 11.69 | 12.27 | 4.53 | 6.31 | 2.84 | 26.78 |
| Tairawhiti | 13.19 | 13.94 | 6.85 | 9.86 | 2.31 | 33.18 |
| Taranaki | 13.39 | 13.44 | 6.79 | 9.32 | 1.61 | 43.25 |
| Waikato | 13.22 | 13.17 | 6.35 | 8.70 | 1.15 | 36.00 |
| Wairarapa | 9.75 | 7.68 | 4.66 | 5.88 | 0.84 | 29.72 |
| Waitemata | 9.75 | 10.920 | 5.65 | 8.03 | 0.35 | 30.98 |
| West Coast | 25.14 | 26.32 | 10.99 | 14.04 | 5.01 | 57.07 |
| Whanganui | 9.18 | 9.40 | 4.66 | 5.93 | 0.58 | 30.88 |

*Supplementary Table 11: Summary statistics for the examined total monthly rainfall (cm)(Total Rainfall) lagged one month for each New Zealand District Health Board in the period 1 January 1999 to 31 December 2017.*

| District health board | Median | Mean | Standard deviation | Interquartile range | Minimum | Maximum |
| --- | --- | --- | --- | --- | --- | --- |
| Auckland | 9.47 | 10.48 | 6.50 | 7.95 | 0.50 | 40.10 |
| Bay of Plenty | 10.69 | 12.28 | 7.11 | 8.19 | 1.10 | 43.54 |
| Canterbury | 8.68 | 9.55 | 4.65 | 6.17 | 1.70 | 24.53 |
| Capital and Coast | 7.96 | 8.89 | 5.08 | 6.31 | 0.36 | 35.38 |
| Counties Manukau | 9.09 | 9.58 | 5.23 | 7.62 | 0.66 | 27.87 |
| Hawke' s Bay | 7.26 | 8.46 | 5.14 | 7.11 | 0.60 | 23.85 |
| Hutt Valley | 10.54 | 11.19 | 6.38 | 7.99 | 1.44 | 42.32 |
| Lakes | 9.06 | 9.76 | 5.90 | 7.81 | 0.28 | 35.85 |
| MidCentral | 9.30 | 9.83 | 4.94 | 5.81 | 0.51 | 34.67 |
| Nelson Marlborough | 11.12 | 11.98 | 6.44 | 8.75 | 0.72 | 31.51 |
| Northland | 10.10 | 11.15 | 6.52 | 8.50 | 0.72 | 37.43 |
| South Canterbury | 5.99 | 6.77 | 3.75 | 4.38 | 1.11 | 21.42 |
| Southern | 11.59 | 12.26 | 4.53 | 6.31 | 2.84 | 26.78 |
| Tairawhiti | 13.19 | 13.93 | 6.85 | 9.86 | 2.31 | 33.18 |
| Taranaki | 13.39 | 13.48 | 6.74 | 9.20 | 1.64 | 43.25 |
| Waikato | 13.22 | 13.20 | 6.32 | 8.55 | 1.15 | 36.00 |
| Wairarapa | 6.75 | 7.67 | 4.66 | 5.91 | 0.84 | 29.72 |
| Waitemata | 9.75 | 10.22 | 5.62 | 7.96 | 0.35 | 30.98 |
| West Coast | 25.31 | 26.42 | 11.02 | 14.22 | 5.01 | 57.07 |
| Whanganui | 9.18 | 9.39 | 4.66 | 5.93 | 0.58 | 30.88 |

*Supplementary Table 12: Summary statistics for the examined total monthly rainfall (cm)(Total Rainfall) lagged two months for each New Zealand District Health Board in the period 1 January 1999 to 31 December 2017.*

| District Health Board | Median | Mean | Standard deviation | Interquartile range | Minimum | Maximum |
| --- | --- | --- | --- | --- | --- | --- |
| Auckland | 9.47 | 10.49 | 6.49 | 7.91 | 0.50 | 40.10 |
| Bay of Plenty | 10.81 | 12.31 | 7.09 | 8.17 | 1.10 | 43.54 |
| Canterbury | 8.68 | 9.54 | 4.66 | 6.17 | 1.70 | 24.53 |
| Capital and Coast | 7.96 | 8.91 | 5.06 | 6.23 | 0.36 | 35.38 |
| Counties Manukau | 9.09 | 9.59 | 5.22 | 7.52 | 0.66 | 27.87 |
| Hawke’s Bay | 7.34 | 8.48 | 5.12 | 7.04 | 0.60 | 23.85 |
| Hutt Valley | 10.54 | 11.19 | 6.37 | 7.99 | 1.44 | 42.32 |
| Lakes | 9.04 | 9.73 | 5.91 | 7.81 | 0.28 | 35.85 |
| MidCentral | 9.36 | 9.86 | 4.92 | 5.78 | 0.51 | 34.67 |
| Nelson Marlborough | 11.12 | 11.99 | 6.42 | 8.73 | 0.72 | 31.51 |
| Northland | 10.20 | 11.18 | 6.52 | 8.50 | 0.72 | 37.43 |
| South Canterbury | 5.99 | 6.78 | 3.74 | 4.38 | 1.11 | 21.42 |
| Southern | 11.59 | 12.25 | 4.54 | 6.31 | 2.84 | 26.78 |
| Tairawhiti | 13.19 | 13.94 | 6.85 | 9.86 | 2.31 | 33.18 |
| Taranaki | 13.39 | 13.50 | 6.73 | 8.92 | 1.64 | 43.25 |
| Waikato | 13.22 | 13.20 | 6.32 | 8.70 | 1.15 | 36.00 |
| Wairarapa | 6.75 | 7.70 | 4.64 | 5.88 | 0.84 | 29.72 |
| Waitemata | 9.75 | 10.24 | 5.61 | 7.91 | 0.35 | 30.98 |
| West Coast | 25.31 | 26.36 | 11.10 | 14.22 | 5.01 | 57.07 |
| Whanganui | 9.18 | 9.40 | 4.66 | 5.90 | 0.58 | 30.88 |

Supplementary Table 13: Summary statistics for the examined total monthly rainfall (cm) (Total Rainfall) lagged three months for each New Zealand District Health Board in the period 1 January 1999 to 31 December 2017.

| District Health Board | Median | Mean | Standard deviation | Interquartile range | Minimum | Maximum |
| --- | --- | --- | --- | --- | --- | --- |
| Auckland | 9.56 | 10.51 | 6.49 | 7.91 | 0.50 | 40.10 |
| Bay of Plenty | 10.81 | 12.31 | 7.09 | 8.17 | 1.10 | 43.54 |
| Canterbury | 8.68 | 9.57 | 4.70 | 6.17 | 1.70 | 24.53 |
| Capital | 8.02 | 9.00 | 5.18 | 6.28 | 0.36 | 35.38 |
| Counties Manukau | 9.09 | 9.60 | 5.22 | 7.52 | 0.66 | 27.87 |
| Hawke's Bay | 7.26 | 8.45 | 5.14 | 7.11 | 0.60 | 23.85 |
| Hutt Valley | 10.60 | 11.34 | 6.64 | 8.14 | 1.44 | 42.32 |
| Lakes | 9.06 | 9.75 | 5.91 | 7.81 | 0.28 | 35.85 |
| MidCentral | 9.47 | 9.94 | 5.05 | 5.86 | 0.51 | 34.67 |
| Nelson | 11.12 | 12.10 | 6.64 | 8.92 | 0.72 | 37.44 |
| Northland | 10.27 | 11.20 | 6.50 | 8.44 | 0.72 | 37.43 |
| South Canterbury | 5.99 | 6.80 | 3.77 | 4.41 | 1.11 | 21.42 |
| Southern | 11.69 | 12.32 | 4.56 | 6.24 | 2.84 | 26.78 |
| Tairawhiti | 13.19 | 13.92 | 6.87 | 9.86 | 2.31 | 33.18 |
| Taranaki | 13.49 | 13.68 | 7.15 | 9.03 | 1.64 | 50.31 |
| Waikato | 13.32 | 13.24 | 6.35 | 8.80 | 1.15 | 36.00 |
| Wairarapa | 6.77 | 7.72 | 4.65 | 5.94 | 0.84 | 29.72 |
| Waitemata | 9.75 | 10.25 | 5.61 | 7.91 | 0.35 | 30.98 |
| West Coast | 25.40 | 26.54 | 11.29 | 14.44 | 5.01 | 58.72 |
| Whanganui | 9.18 | 9.45 | 4.74 | 5.98 | 0.58 | 30.88 |

Supplementary Material S4: Model of all serovars supplementary tables

*Supplementary Table S14: Table of the summary statistics for the number of leptospirosis cases included in the analysis that were reported in New Zealand over the period 1 January 1999 to 31 December 2017 by District Health Board, season, and year.*

|  |  | Number of cases per month | | | | |  | Total number of cases |
| --- | --- | --- | --- | --- | --- | --- | --- | --- |
| Variable name |  | **Median** | **Mean (SD)** | **Interquartile range** | **Minimum value** | **Maximum value** | **Number of months** |  |
| Season |  |  |  |  |  |  |  |  |
| Autumn |  | 6.00 | 7.01 (±4.00) | 4.00 | 1 | 21 | 57 | 404 |
| Spring |  | 6.00 | 6.83 (±3.79) | 5.00 | 0 | 17 | 57 | 389 |
| Summer |  | 7.00 | 6.67 (±3.60) | 4.00 | 1 | 16 | 57 | 380 |
| Winter |  | 6.00 | 6.51 (±3.57) | 5.00 | 1 | 20 | 57 | 371 |
| District Health Board | | |  |  |  |  |  |  |
| Auckland |  | 0.00 | 0.03 (±0.16) | 0.00 | 0 | 1 | 228 | 6 |
| Bay of Plenty |  | 0.00 | 0.33 (±0.62) | 1.00 | 0 | 4 | 228 | 75 |
| Canterbury |  | 0.00 | 0.50 (±0.76) | 1.00 | 0 | 4 | 228 | 113 |
| Capital and Coast |  | 0.00 | 0.04 (±0.21) | 0.00 | 0 | 1 | 228 | 10 |
| Counties Manukau |  | 0.00 | 0.16 (±0.39) | 0.00 | 0 | 2 | 228 | 36 |
| Hawke’s Bay |  | 1.00 | 0.89 (±1.14) | 1.00 | 0 | 5 | 228 | 202 |
| Hutt Valley |  | 0.00 | 0.03 (±0.20) | 0.00 | 0 | 2 | 228 | 7 |
| Lakes |  | 0.00 | 0.12 (±0.37) | 0.00 | 0 | 2 | 224 | 27 |
| Mid Central |  | 0.00 | 0.58 (±0.82) | 1.00 | 0 | 4 | 228 | 132 |
| Northland |  | 0.00 | 0.53 (±0.84) | 1.00 | 0 | 5 | 228 | 121 |
| Nelson Marlborough |  | 0.00 | 0.32 (±0.69) | 0.00 | 0 | 4 | 228 | 72 |
| South Canterbury |  | 0.00 | 0.17 (±0.44) | 0.00 | 0 | 2 | 228 | 39 |
| Southern |  | 0.00 | 0.44 (±0.78) | 1.00 | 0 | 6 | 228 | 100 |
| Tairawhiti |  | 0.00 | 0.27 (±0.69) | 0.00 | 0 | 5 | 228 | 61 |
| Taranaki |  | 0.00 | 0.32 (±0.58) | 1.00 | 0 | 3 | 228 | 73 |
| Waikato |  | 1.00 | 1.27 (±1.52) | 2.00 | 0 | 12 | 228 | 290 |
| Wairarapa |  | 0.00 | 0.13 (±0.38) | 0.00 | 0 | 2 | 228 | 30 |
| Waitemata |  | 0.00 | 0.15 (±0.44) | 0.00 | 0 | 3 | 228 | 35 |
| West Coast |  | 0.00 | 0.21 (±0.48) | 0.00 | 0 | 2 | 228 | 47 |
| Whanganui |  | 0.00 | 0.30 (±0.61) | 0.00 | 0 | 3 | 228 | 68 |
| Year |  |  |  |  |  |  |  |  |
| 1999 |  | 4.00 | 3.83 (±1.95) | 2.50 | 1 | 7 | 12 | 46 |
| 2000 |  | 5.50 | 6.50 (±3.45) | 2.75 | 3 | 13 | 12 | 78 |
| 2001 |  | 8.00 | 7.33 (±2.81) | 3.75 | 2 | 11 | 12 | 88 |
| 2002 |  | 11.00 | 11.17 (±3.41) | 6.50 | 7 | 16 | 12 | 134 |
| 2003 |  | 8.00 | 9.08 (±4.01) | 4.25 | 5 | 16 | 12 | 109 |
| 2004 |  | 8.00 | 7.67 (±2.15) | 3.25 | 4 | 11 | 12 | 92 |
| 2005 |  | 6.50 | 6.50 (±2.84) | 3.25 | 2 | 12 | 12 | 78 |
| 2006 |  | 6.00 | 6.58 (±3.12) | 3.25 | 1 | 13 | 12 | 79 |
| 2007 |  | 3.50 | 5.08 (±3.78) | 3.75 | 1 | 14 | 12 | 61 |
| 2008 |  | 9.00 | 8.75 (±2.83) | 3.50 | 4 | 13 | 12 | 105 |
| 2009 |  | 4.00 | 5.17 (±3.27) | 4.25 | 2 | 13 | 12 | 62 |
| 2010 |  | 5.50 | 5.92 (±2.39) | 2.50 | 2 | 10 | 12 | 71 |
| 2011 |  | 5.50 | 5.08 (±1.93) | 1.50 | 1 | 7 | 12 | 61 |
| 2012 |  | 8.50 | 8.67 (±4.12) | 4.25 | 2 | 15 | 12 | 104 |
| 2013 |  | 5.00 | 4.83 (±2.44) | 3.00 | 2 | 11 | 12 | 58 |
| 2014 |  | 4.00 | 4.33 (±2.87) | 3.25 | 1 | 10 | 12 | 52 |
| 2015 |  | 5.50 | 4.67 (±3.23) | 4.75 | 0 | 10 | 12 | 56 |
| 2016 |  | 6.00 | 6.42 (±2.50) | 1.75 | 2 | 11 | 12 | 77 |
| 2017 |  | 9.00 | 11.08(±5.43) | 6.00 | 4 | 21 | 12 | 133 |

*Supplementary Table S15: Table showing the results of univariable analyses measuring the crude association between the explanatory variables and the incidence rate of reported cases of leptospirosis included the analysis in New Zealand in the period 1 January 1999 to 31 December 2017 using a negative binomial generalised linear regression model.*

| Variable name | Beta Coefficient | Incidence Rate Ratio (IRR) | IRR Confidence Interval  2.5% 97.5% | | Wald test p-value | Likelihood ratio test p-value |
| --- | --- | --- | --- | --- | --- | --- |
| Animal density (animals/km^2^) | 0.005 | 1.005 | 1.004 | 1.005 | **<0.001** | **<0.001** |
| Total rainfall | 0.018 | 1.018 | 1.007 | 1.029 | **<0.01** | **<0.01** |
| Total rainfall_1* | 0.030 | 1.030 | 1.020 | 1.041 | **<0.001** | **<0.001** |
| Total rainfall_2* | 0.037 | 1.037 | 1.027 | 1.048 | **<0.001** | **<0.001** |
| Total rainfall_3* | 0.034 | 1.034 | 1.024 | 1.045 | **<0.001** | **<0.001** |
| Season |  |  |  |  |  |  |
| Autumn | Reference |  |  |  |  | 0.87 |
| Spring | -0.012 | 0.988 | 0.807 | 1.209 | 0.90 |  |
| Summer | -0.072 | 0.931 | 0.760 | 1.140 | 0.47 |  |
| Winter | -0.064 | 0.938 | 0.765 | 1.149 | 0.51 |  |
| District Health Board |  |  |  |  |  |  |
| Northland | Reference |  |  |  |  | **<0.001** |
| Auckland | -4.021 | 0.0.18 | 0.007 | 0.038 | **<0.001** |  |
| Bay of Plenty | -0.742 | 0.476 | 0.345 | 0.654 | **<0.001** |  |
| Canterbury | -1.196 | 0.302 | 0.226 | 0.405 | **<0.001** |  |
| Capital and Coast | -3.071 | 0.046 | 0.022 | 0.085 | **<0.001** |  |
| Counties Manukau | -2.290 | 0.101 | 0.067 | 0.149 | **<0.001** |  |
| Hawke's Bay | 0.528 | 1.696 | 1.303 | 2.214 | **<0.001** |  |
| Hutt Valley | -2.746 | 0.064 | 0.027 | 0.129 | **<0.001** |  |
| Lakes | -1.054 | 0.348 | 0.220 | 0.533 | **<0.001** |  |
| MidCentral | 0.033 | 1.033 | 0.779 | 1.372 | 0.82 |  |
| Nelson Marlborough | -0.374 | 0.688 | 0.496 | 0.948 | **<0.05** |  |
| South Canterbury | -0.099 | 0.905 | 0.608 | 1.321 | 0.61 |  |
| Southern | -0.833 | 0.435 | 0.322 | 0.586 | **<0.001** |  |
| Tairawhiti | 0.534 | 1.705 | 1.210 | 2.381 | **<0.01** |  |
| Taranaki | -0.151 | 0.860 | 0.621 | 1.183 | 0.36 |  |
| Waikato | 0.042 | 1.043 | 0.811 | 1.347 | 0.75 |  |
| Wairarapa | -0.051 | 0.950 | 0.613 | 1.432 | 0.81 |  |
| Waitemata | -2.427 | 0.088 | 0.058 | 0.130 | **<0.001** |  |
| West Coast | 0.640 | 1.896 | 1.306 | 2.712 | **<0.01** |  |
| Whanganui | 0.321 | 1.378 | 0.989 | 1.907 | 0.06 |  |
| Year |  |  |  |  |  |  |
| 1999 | Reference |  |  |  |  | **<0.001** |
| 2000 | 0.605 | 1.831 | 1.139 | 2.962 | **<0.01** |  |
| 2001 | 0.704 | 2.022 | 1.268 | 3.247 | **<0.01** |  |
| 2002 | 1.207 | 3.345 | 2.141 | 5.277 | **<0.001** |  |
| 2003 | 0.865 | 2.374 | 1.506 | 3.776 | **<0.001** |  |
| 2004 | 0.721 | 2.057 | 1.294 | 3.296 | **<0.01** |  |
| 2005 | 0.594 | 1.812 | 1.129 | 2.928 | **<0.05** |  |
| 2006 | 0.619 | 1.856 | 1.157 | 2.998 | **<0.01** |  |
| 2007 | 0.254 | 1.289 | 0.790 | 2.114 | 0.29 |  |
| 2008 | 0.931 | 2.536 | 1.600 | 4.054 | **<0.001** |  |
| 2009 | 0.246 | 1.279 | 0.784 | 2.097 | 0.31 |  |
| 2010 | 0.514 | 1.672 | 1.033 | 2.720 | **<0.05** |  |
| 2011 | 0.271 | 1.311 | 0.801 | 2.152 | 0.26 |  |
| 2012 | 0.823 | 2.278 | 1.440 | 3.636 | **<0.001** |  |
| 2013 | 0.203 | 1.225 | 0.747 | 2.015 | 0.40 |  |
| 2014 | 0.068 | 1.070 | 0.647 | 1.773 | 0.78 |  |
| 2015 | 0.130 | 1.139 | 0.692 | 1.880 | 0.59 |  |
| 2016 | 0.382 | 1.465 | 0.912 | 2.370 | 0.11 |  |
| 2017 | 0.918 | 2.505 | 1.600 | 3.963 | **<0.001** |  |
| Year (continuous) ^#^ | -0.015 | 0.985 | 0.972 | 0.998 | **<0.05** | **<0.05** |
| Polynomial(year, 3)1^##^ | -5.507 | 0.004 | 0.000 | 0.499 | **<0.05** | **<0.05** |
| Polynomial(year, 3)2^##^ | -1.359 | 0.257 | 0.002 | 33.139 | 0.56 |  |
| Polynomial(year, 3)3^##^ | 13.129 | 5.03E+05 | 4.10E+03 | 6.30E+07 | **<0.001** |  |

** Total rainfall_1* is the total monthly rainfall in cm the month prior to the month a case is reported in.

*** Total rainfall_2* is the total monthly rainfall in cm in the month, two months prior to the month a case is reported in.

****Total rainfall_3* is the total monthly rainfall in cm in the month, three months prior to the month a case is reported in.

^#^Year as a linear variable.

^##^Year as a cuboid polynomial variable.

Supplementary Material S5: Supplementary tables of the model for Hardjo cases

*Supplementary Table S16: Table of the summary statistics for the number of* Hardjo *cases included in the analysis that were reported in New Zealand between 1 January 1999 and 31 December 2017 by District Health Board, season, and year.*

| Categorical variables | | | | | | | | | |
| --- | --- | --- | --- | --- | --- | --- | --- | --- | --- |
|  |  |  | **Number of cases per month** | | | | |  | **Total number of cases** |
|  |  |  | **Median** | **Mean (SD)** | **Interquartile range** | **Minimum value** | **Maximum value** | **Number of months** |  |
| Season |  |  |  |  |  |  |  |  |  |
| Autumn |  |  | 2.00 | 1.81 (±1.68) | 3.00 | 0 | 7 | 57 | 103 |
| Spring |  |  | 2.00 | 2.65 (±2.13) | 3.00 | 0 | 10 | 57 | 151 |
| Summer |  |  | 1.00 | 1.93 (±2.01) | 3.00 | 0 | 10 | 57 | 110 |
| Winter |  |  | 1.00 | 1.98 (±1.87) | 3.00 | 0 | 6 | 57 | 113 |
| District Health Board |  |  |  |  |  |  |  |  |  |
| Auckland |  |  | 0.00 | 0.00 (±0.00) | 0.00 | 0 | 0 | 228 | 0 |
| Bay of Plenty |  |  | 0.00 | 0.03 (±0.17) | 0.00 | 0 | 1 | 228 | 7 |
| Canterbury |  |  | 0.00 | 0.20 (±0.49) | 0.00 | 0 | 3 | 228 | 45 |
| Capital and Coast |  |  | 0.00 | 0.00 (±0.07) | 0.00 | 0 | 1 | 228 | 1 |
| Counties Manukau |  |  | 0.00 | 0.04 (±0.18) | 0.00 | 0 | 1 | 228 | 8 |
| Hawke’s Bay |  |  | 0.00 | 0.40 (±0.76) | 1.00 | 0 | 5 | 228 | 90 |
| Hutt Valley |  |  | 0.00 | 0.01 (±0.09) | 0.00 | 0 | 1 | 228 | 2 |
| Lakes |  |  | 0.00 | 0.01 (±0.09) | 0.00 | 0 | 1 | 224 | 2 |
| Mid Central |  |  | 0.00 | 0.23 (±0.49) | 0.00 | 0 | 3 | 228 | 53 |
| Northland |  |  | 0.00 | 0.16 (±0.43) | 0.00 | 0 | 3 | 228 | 20 |
| Nelson Marlborough |  |  | 0.00 | 0.09 (±0.37) | 0.00 | 0 | 3 | 228 | 36 |
| South Canterbury |  |  | 0.00 | 0.07 (±0.30) | 0.00 | 0 | 2 | 228 | 16 |
| Southern |  |  | 0.00 | 0.22 (±0.59) | 0.00 | 0 | 5 | 228 | 50 |
| Tairawhiti |  |  | 0.00 | 0.05 (±0.23) | 0.00 | 0 | 2 | 228 | 11 |
| Taranaki |  |  | 0.00 | 0.07 (±0.26) | 0.00 | 0 | 1 | 228 | 16 |
| Waikato |  |  | 0.00 | 0.32 (±0.69) | 0.00 | 0 | 5 | 228 | 73 |
| Wairarapa |  |  | 0.00 | 0.06 (±0.26) | 0.00 | 0 | 2 | 228 | 14 |
| Waitemata |  |  | 0.00 | 0.00 (±0.07) | 0.00 | 0 | 1 | 228 | 1 |
| West Coast |  |  | 0.00 | 0.06 (±0.25) | 0.00 | 0 | 2 | 228 | 13 |
| Whanganui |  |  | 0.00 | 0.08 (±0.31) | 0.00 | 0 | 2 | 228 | 19 |
| Year |  |  |  |  |  |  |  |  |  |
| 1999 |  |  | 0.00 | 0.75 (±1.22) | 1.25 | 0 | 3 | 12 | 9 |
| 2000 |  |  | 3.00 | 2.67 (±1.23) | 1.00 | 0 | 5 | 12 | 32 |
| 2001 |  |  | 3.00 | 2.08 (±1.62) | 2.25 | 0 | 5 | 12 | 25 |
| 2002 |  |  | 3.00 | 3.33 (±1.78) | 2.25 | 1 | 7 | 12 | 40 |
| 2003 |  |  | 4.00 | 4.50 (±3.34) | 4.50 | 0 | 10 | 12 | 54 |
| 2004 |  |  | 4.50 | 4.17 (±1.75) | 2.50 | 2 | 7 | 12 | 50 |
| 2005 |  |  | 3.00 | 2.83 (±1.75) | 2.25 | 0 | 6 | 12 | 34 |
| 2006 |  |  | 1.00 | 2.25 (±2.09) | 2.25 | 0 | 7 | 12 | 27 |
| 2007 |  |  | 1.00 | 1.50 (±1.51) | 1.50 | 0 | 5 | 12 | 18 |
| 2008 |  |  | 2.00 | 1.92 (±1.51) | 1.25 | 0 | 5 | 12 | 23 |
| 2009 |  |  | 2.00 | 1.92 (±1.93) | 1.50 | 0 | 7 | 12 | 23 |
| 2010 |  |  | 0.00 | 0.50 (±0.80) | 1.00 | 0 | 2 | 12 | 6 |
| 2011 |  |  | 1.00 | 0.83 (±0.83) | 1.25 | 0 | 2 | 12 | 10 |
| 2012 |  |  | 2.00 | 2.42 (±2.15) | 2.50 | 0 | 6 | 12 | 29 |
| 2013 |  |  | 1.00 | 1.25 (±1.71) | 2.00 | 0 | 6 | 12 | 15 |
| 2014 |  |  | 1.00 | 1.67 (±0.98) | 1.00 | 1 | 4 | 12 | 20 |
| 2015 |  |  | 0.50 | 1.17 (±1.47) | 2.25 | 0 | 4 | 12 | 14 |
| 2016 |  |  | 1.50 | 1.42 (±1.31) | 2.00 | 0 | 4 | 12 | 17 |
| 2017 |  |  | 3.00 | 2.58 (±1.51) | 3.00 | 0 | 4 | 12 | 31 |

*Supplementary Table S17: Summary of average monthly incidence (number of cases /100 000 people) of reported human Hardjo leptospirosis cases included in the analysis in each New Zealand District Health Board during the period 1 January 1999 to 31 December 2017.*

| District Health Board | Median | Mean | Standard deviation | Interquartile range | Minimum | Maximum |
| --- | --- | --- | --- | --- | --- | --- |
| Auckland | 0.00 | 0.00 | 0.00 | 0.00 | 0.00 | 0.00 |
| Bay of Plenty | 0.00 | 0.02 | 0.08 | 0.00 | 0.00 | 0.51 |
| Canterbury | 0.00 | 0.04 | 0.10 | 0.00 | 0.00 | 0.65 |
| Capital and Coast | 0.00 | 0.00 | 0.02 | 0.00 | 0.00 | 0.37 |
| Counties Manukau | 0.00 | 0.01 | 0.04 | 0.00 | 0.00 | 0.24 |
| Hawke's Bay | 0.00 | 0.26 | 0.50 | 0.61 | 0.00 | 3.33 |
| Hutt Valley | 0.00 | 0.01 | 0.06 | 0.00 | 0.00 | 0.71 |
| Lakes | 0.00 | 0.01 | 0.09 | 0.00 | 0.00 | 0.99 |
| MidCentral | 0.00 | 0.14 | 0.30 | 0.00 | 0.00 | 1.70 |
| Nelson Marlborough | 0.00 | 0.07 | 0.28 | 0.00 | 0.00 | 2.35 |
| Northland | 0.00 | 0.10 | 0.29 | 0.00 | 0.00 | 1.96 |
| South Canterbury | 0.00 | 0.13 | 0.56 | 0.00 | 0.00 | 3.70 |
| Southern | 0.00 | 0.07 | 0.20 | 0.00 | 0.00 | 1.71 |
| Tairawhiti | 0.00 | 0.10 | 0.51 | 0.00 | 0.00 | 4.40 |
| Taranaki | 0.00 | 0.06 | 0.23 | 0.00 | 0.00 | 0.94 |
| Waikato | 0.00 | 0.09 | 0.19 | 0.00 | 0.00 | 1.38 |
| Wairarapa | 0.00 | 0.15 | 0.63 | 0.00 | 0.00 | 5.05 |
| Waitemata | 0.00 | 0.00 | 0.01 | 0.00 | 0.00 | 0.17 |
| West Coast | 0.00 | 0.18 | 0.78 | 0.00 | 0.00 | 6.37 |
| Whanganui | 0.00 | 0.13 | 0.48 | 0.00 | 0.00 | 3.16 |

*Supplementary Table S18: Table showing the results of univariable analyses measuring the crude association between the explanatory variables and the incidence rate of reported* Hardjo *cases included in the analysis in New Zealand in the period 1 January 1999 to 31 December 2017 using Poisson models.*

| Variable name | Beta Coefficient | Incidence rate ratio | IRR Confidence Interval 2.5% 97.5% | | Wald test p-value | Likelihood ratio test p-value |
| --- | --- | --- | --- | --- | --- | --- |
|  |  |  |  |  |  |  |
| Animal density (animals/km2) | 0.007 | 1.007 | 1.007 | 1.008 | 0.000 | **<0.001** |
| Total monthly Rainfall (cm) | |  |  |  |  |  |
| Total Rainfall | 0.020 | 1.020 | 1.006 | 1.034 | 0.004 | **<0.01** |
| Total Rainfall_1 | 0.023 | 1.023 | 1.009 | 1.037 | 0.001 | **<0.01** |
| Total Rainfall_2 | 0.030 | 1.030 | 1.017 | 1.044 | 0.000 | **<0.001** |
| Total Rainfall_3 | 0.026 | 1.026 | 1.013 | 1.040 | 0.000 | **<0.001** |
| Season |  |  |  |  |  |  |
| Autumn | Reference |  |  |  |  | **<0.05** |
| Spring | 0.383 | 1.467 | 1.142 | 1.885 | 0.003 |  |
| Summer | 0.067 | 1.069 | 0.817 | 1.398 | 0.627 |  |
| Winter | 0.093 | 1.097 | 0.840 | 1.433 | 0.496 |  |
| District Health Board |  |  |  |  |  |  |
| Northland | Reference |  |  |  |  | **<0.001** |
| Auckland | -17.469 | 0.000 | 0.000 | Inf | 0.963 |  |
| Bay of Plenty | -1.902 | 0.149 | 0.066 | 0.335 | 0.000 |  |
| Canterbury | -0.908 | 0.403 | 0.260 | 0.625 | 0.000 |  |
| Capital and Coast | -4.161 | 0.016 | 0.002 | 0.114 | 0.000 |  |
| Counties Manukau | -2.581 | 0.076 | 0.035 | 0.163 | 0.000 |  |
| Hawke's Bay | 0.935 | 2.547 | 1.731 | 3.749 | 0.000 |  |
| Hutt Valley | -2.784 | 0.062 | 0.015 | 0.257 | 0.000 |  |
| Lakes | -2.443 | 0.087 | 0.021 | 0.361 | 0.001 |  |
| MidCentral | 0.333 | 1.396 | 0.914 | 2.131 | 0.123 |  |
| Nelson Marlborough | -0.443 | 0.642 | 0.372 | 1.109 | 0.112 |  |
| South Canterbury | 0.222 | 1.249 | 0.693 | 2.250 | 0.460 |  |
| Southern | -0.313 | 0.731 | 0.476 | 1.122 | 0.152 |  |
| Tairawhiti | 0.033 | 1.034 | 0.526 | 2.031 | 0.923 |  |
| Taranaki | -0.455 | 0.634 | 0.352 | 1.143 | 0.130 |  |
| Waikato | -0.120 | 0.887 | 0.595 | 1.323 | 0.557 |  |
| Wairarapa | 0.402 | 1.494 | 0.806 | 2.770 | 0.202 |  |
| Waitemata | -4.768 | 0.008 | 0.001 | 0.062 | 0.000 |  |
| West Coast | 0.569 | 1.766 | 0.937 | 3.330 | 0.079 |  |
| Whanganui | 0.259 | 1.295 | 0.743 | 2.258 | 0.362 |  |
| Year |  |  |  |  |  |  |
| 1999 | Reference |  |  |  |  | **<0.001** |
| 2000 | 1.263 | 3.535 | 1.687 | 7.405 | 0.001 |  |
| 2001 | 1.010 | 2.745 | 1.281 | 5.881 | 0.009 |  |
| 2002 | 1.463 | 4.317 | 2.095 | 8.896 | 0.000 |  |
| 2003 | 1.743 | 5.714 | 2.821 | 11.571 | 0.000 |  |
| 2004 | 1.651 | 5.212 | 2.563 | 10.599 | 0.000 |  |
| 2005 | 1.254 | 3.505 | 1.681 | 7.307 | 0.001 |  |
| 2006 | 1.011 | 2.749 | 1.293 | 5.846 | 0.009 |  |
| 2007 | 0.597 | 1.816 | 0.816 | 4.042 | 0.144 |  |
| 2008 | 0.833 | 2.301 | 1.065 | 4.972 | 0.034 |  |
| 2009 | 0.823 | 2.278 | 1.054 | 4.922 | 0.036 |  |
| 2010 | -0.532 | 0.588 | 0.209 | 1.651 | 0.313 |  |
| 2011 | -0.028 | 0.972 | 0.395 | 2.392 | 0.951 |  |
| 2012 | 1.031 | 2.803 | 1.327 | 5.922 | 0.007 |  |
| 2013 | 0.364 | 1.439 | 0.630 | 3.288 | 0.388 |  |
| 2014 | 0.635 | 1.887 | 0.859 | 4.144 | 0.114 |  |
| 2015 | 0.258 | 1.294 | 0.560 | 2.990 | 0.546 |  |
| 2016 | 0.435 | 1.545 | 0.689 | 3.467 | 0.291 |  |
| 2017 | 1.011 | 2.749 | 1.309 | 5.775 | 0.008 |  |
| Year (continuous) | -0.047 | 0.954 | 0.938 | 0.970 | 0.000 | **<0.001** |
| Polynomial (year,3)1 | -17.872 | 0.000 | 0.000 | 0.000 | 0.000 | **<0.001** |
| Polynomial (year,3)2 | -1.072 | 0.342 | 0.001 | 172.005 | 0.735 |  |
| Polynomial (year,3)3 | 23.443 | 1.517e+10 | 2.518e+7 | 9.142e+12 | 0.000 |  |

** Total rainfall_1* is the total monthly rainfall in cm the month prior to the month a case is reported in.

*** Total rainfall_2* is the total monthly rainfall in cm in the month, two months prior to the month a case is reported in.

****Total rainfall_3* is the total monthly rainfall in cm in the month, three months prior to the month a case is reported in.

^#^Year as a linear variable.

^##^Year as a cuboid polynomial variable.

^###^Result is related to no cases of Hardjo recorded in the Auckland DHB over the study period.

Supplementary Material S6: Supplementary tables of the model for Pomona cases

*Supplementary Table S19: Table of the summary statistics for the number of reported Pomona cases included in the analysis in New Zealand between 1 January 1999 and 31 December 2017 by District Health Board, season, and year.*

| Categorical variables | | | | | | | | | |
| --- | --- | --- | --- | --- | --- | --- | --- | --- | --- |
|  |  |  | **Number of cases per month** | | | | |  | **Total number of cases** |
|  |  |  | **Median** | **Mean (SD)** | **Interquartile range** | **Minimum value** | **Maximum value** | **Number of months** |  |
| Season |  |  |  |  |  |  |  |  |  |
| Autumn |  |  | 1.0 | 1.21 (±1.45) | 2.00 | 0 | 5 | 57 | 69 |
| Spring |  |  | 1.0 | 1.23 (±1.05) | 2.00 | 0 | 4 | 57 | 64 |
| Summer |  |  | 1.0 | 1.35 (±1.53) | 2.00 | 0 | 7 | 57 | 77 |
| Winter |  |  | 1.0 | 0.91 (±1.07) | 1.00 | 0 | 5 | 57 | 52 |
| District Health Board | | | | |  |  |  |  |  |
| Auckland |  |  | 0.0 | 0.00 (±0.00) | 0.00 | 0 | 0 | 228 | 0 |
| Bay of Plenty |  |  | 0.0 | 0.03 (±0.19) | 0.00 | 0 | 2 | 228 | 6 |
| Canterbury |  |  | 0.0 | 0.05 (±0.22) | 0.00 | 0 | 1 | 228 | 12 |
| Capital and Coast |  |  | 0.0 | 0.00 (±0.07) | 0.00 | 0 | 1 | 228 | 1 |
| Counties Manukau |  |  | 0.0 | 0.00 (±0.07) | 0.00 | 0 | 1 | 228 | 1 |
| Hawke’s Bay |  |  | 0.0 | 0.29 (±0.66) | 0.00 | 0 | 5 | 228 | 65 |
| Hutt Valley |  |  | 0.0 | 0.01 (±0.09) | 0.00 | 0 | 1 | 228 | 2 |
| Lakes |  |  | 0.0 | 0.01 (±0.09) | 0.00 | 0 | 1 | 224 | 2 |
| Mid Central |  |  | 0.0 | 0.21 (±0.48) | 0.00 | 0 | 3 | 228 | 47 |
| Nelson Marlborough |  |  | 0.0 | 0.03 (±0.25) | 0.00 | 0 | 3 | 228 | 6 |
| Northland |  |  | 0.0 | 0.04 (±0.21) | 0.00 | 0 | 1 | 228 | 10 |
| South Canterbury |  |  | 0.0 | 0.03 (±0.17) | 0.00 | 0 | 1 | 228 | 7 |
| Southern |  |  | 0.0 | 0.03 (±0.16) | 0.00 | 0 | 1 | 228 | 6 |
| Tairawhiti |  |  | 0.0 | 0.08 (±0.32) | 0.00 | 0 | 2 | 228 | 19 |
| Taranaki |  |  | 0.0 | 0.03 (±0.16) | 0.00 | 0 | 1 | 228 | 6 |
| Waikato |  |  | 0.0 | 0.21 (±0.43) | 0.00 | 0 | 2 | 228 | 47 |
| Wairarapa |  |  | 0.0 | 0.02 (±0.15) | 0.00 | 0 | 1 | 228 | 5 |
| Waitemata |  |  | 0.0 | 0.00 (±0.07) | 0.00 | 0 | 1 | 228 | 1 |
| West Coast |  |  | 0.0 | 0.00 (±0.07) | 0.00 | 0 | 1 | 228 | 1 |
| Whanganui |  |  | 0.0 | 0.08 (±0.30) | 0.00 | 0 | 2 | 228 | 18 |
| Year |  |  |  |  |  |  |  |  |  |
| 1999 |  |  | 0.5 | 0.58 (±0.67) | 1.00 | 0 | 2 | 12 | 7 |
| 2000 |  |  | 0.5 | 0.58 (±0.67) | 1.00 | 0 | 2 | 12 | 7 |
| 2001 |  |  | 1.5 | 1.58 (±1.08) | 1.25 | 0 | 3 | 12 | 19 |
| 2002 |  |  | 3.0 | 3.50 (±1.73) | 1.25 | 0 | 7 | 12 | 42 |
| 2003 |  |  | 1.0 | 1.25 (±1.14) | 2.00 | 0 | 3 | 12 | 15 |
| 2004 |  |  | 1.5 | 1.75 (±1.42) | 1.25 | 0 | 5 | 12 | 21 |
| 2005 |  |  | 1.0 | 1.25 (±1.14) | 2.00 | 0 | 3 | 12 | 15 |
| 2006 |  |  | 1.0 | 1.33 (±1.97) | 1.00 | 0 | 7 | 12 | 16 |
| 2007 |  |  | 1.0 | 1.08 (±1.51) | 1.00 | 0 | 5 | 12 | 13 |
| 2008 |  |  | 1.0 | 1.00 (±0.74) | 0.50 | 0 | 2 | 12 | 12 |
| 2009 |  |  | 0.0 | 0.42 (±0.67) | 1.00 | 0 | 2 | 12 | 5 |
| 2010 |  |  | 1.0 | 1.00 (±0.95) | 1.25 | 0 | 3 | 12 | 12 |
| 2011 |  |  | 1.0 | 1.17 (±1.03) | 2.00 | 0 | 3 | 12 | 14 |
| 2012 |  |  | 1.5 | 1.83 (±1.64) | 2.25 | 0 | 5 | 12 | 22 |
| 2013 |  |  | 1.0 | 0.75 (±0.75) | 1.00 | 0 | 2 | 12 | 9 |
| 2014 |  |  | 0.0 | 0.42 (±0.51) | 1.00 | 0 | 1 | 12 | 5 |
| 2015 |  |  | 0.5 | 0.67 (±0.78) | 1.00 | 0 | 2 | 12 | 8 |
| 2016 |  |  | 0.0 | 0.42 (±0.51) | 1.00 | 0 | 1 | 12 | 5 |
| 2017 |  |  | 1.0 | 1.25 (±1.14) | 2.00 | 0 | 3 | 12 | 15 |

*Supplementary Table S20: Summary of average monthly incidence (number of cases/100 000 people) of reported human* Pomona *leptospirosis cases included in the analysis in each New Zealand District Health Board during the period 1 January 1999 to 31 December 2017.*

| District Health Board | Median | Mean | Standard deviation | Interquartile range | Minimum | Maximum |
| --- | --- | --- | --- | --- | --- | --- |
| Auckland | 0.00 | 0.00 | 0.00 | 0.00 | 0.00 | 0.00 |
| Bay of Plenty | 0.00 | 0.01 | 0.10 | 0.00 | 0.00 | 1.07 |
| Canterbury | 0.00 | 0.01 | 0.05 | 0.00 | 0.00 | 0.23 |
| Capital and Coast | 0.00 | 0.00 | 0.03 | 0.00 | 0.00 | 0.39 |
| Counties Manukau | 0.00 | 0.00 | 0.02 | 0.00 | 0.00 | 0.26 |
| Hawke's Bay | 0.00 | 0.18 | 0.43 | 0.00 | 0.00 | 3.37 |
| Hutt Valley | 0.00 | 0.01 | 0.07 | 0.00 | 0.00 | 0.71 |
| Lakes | 0.00 | 0.01 | 0.09 | 0.00 | 0.00 | 1.01 |
| MidCentral | 0.00 | 0.12 | 0.29 | 0.00 | 0.00 | 1.83 |
| Nelson Marlborough | 0.00 | 0.02 | 0.19 | 0.00 | 0.00 | 2.35 |
| Northland | 0.00 | 0.03 | 0.14 | 0.00 | 0.00 | 0.70 |
| South Canterbury | 0.00 | 0.06 | 0.31 | 0.00 | 0.00 | 1.86 |
| Southern | 0.00 | 0.01 | 0.05 | 0.00 | 0.00 | 0.35 |
| Tairawhiti | 0.00 | 0.18 | 0.70 | 0.00 | 0.00 | 4.40 |
| Taranaki | 0.00 | 0.02 | 0.14 | 0.00 | 0.00 | 0.94 |
| Waikato | 0.00 | 0.06 | 0.12 | 0.00 | 0.00 | 0.57 |
| Wairarapa | 0.00 | 0.05 | 0.34 | 0.00 | 0.00 | 2.54 |
| Waitemata | 0.00 | 0.00 | 0.01 | 0.00 | 0.00 | 0.22 |
| West Coast | 0.00 | 0.01 | 0.21 | 0.00 | 0.00 | 3.10 |
| Whanganui | 0.00 | 0.12 | 0.48 | 0.00 | 0.00 | 3.17 |

*Supplementary Table S21: Table showing the results of univariable analyses measuring the crude association between the explanatory variables and the incidence rate of reported* Pomona *cases included in the analysis in New Zealand in the period 1 January 1999 to 31 December 2017 using Poisson models.*

| Variable name | Beta Coefficient | Incidence rate ratio (IRR) | IRR Confidence Interval  2.5% 97.5% | | Wald test p-value | Likelihood ratio test p-value |
| --- | --- | --- | --- | --- | --- | --- |
| Animal density (animals/km2) | 0.010 | 1.010 | 1.009 | 1.011 | 0.000 | **<0.001** |
| Season |  |  |  |  |  |  |
| Autumn | Reference |  |  |  |  | **0.16** |
| Spring | -0.074 | 0.928 | 0.661 | 1.304 | 0.668 |  |
| Summer | 0.111 | 1.117 | 0.807 | 1.546 | 0.505 |  |
| Winter | -0.283 | 0.754 | 0.526 | 1.080 | 0.123 |  |
| Total monthly rainfall (cm) |  |  |  |  |  |  |
| Total rainfall | -0.015 | 0.985 | 0.965 | 1.006 | 0.163 | **0.16** |
| Total rainfall_1* | -0.004 | 0.996 | 0.976 | 1.016 | 0.686 | 0.68 |
| Total rainfall_2* | 0.010 | 1.010 | 0.991 | 1.030 | 0.292 | 0.30 |
| Total rainfall_3* | 0.016 | 1.016 | 0.997 | 1.035 | 0.096 | **0.10** |
| District Health Board |  |  |  |  |  |  |
| Northland | Reference |  |  |  |  | **<0.001** |
| Auckland | -16.188 | 0.000 | 0.000 | Inf | 0.966 |  |
| Bay of Plenty | -0.775 | 0.461 | 0.167 | 1.267 | 0.133 |  |
| Canterbury | -0.949 | 0.387 | 0.167 | 0.896 | 0.027 |  |
| Capital and Coast | -2.880 | 0.056 | 0.007 | 0.438 | 0.006 |  |
| Counties Manukau | -3.379 | 0.034 | 0.004 | 0.266 | 0.001 |  |
| Hawke's Bay | 1.890 | 6.623 | 3.403 | 12.888 | 0.000 |  |
| Hutt Valley | -1.503 | 0.222 | 0.049 | 1.015 | 0.052 |  |
| Lakes | -1.162 | 0.313 | 0.069 | 1.428 | 0.134 |  |
| MidCentral | 1.494 | 4.455 | 2.251 | 8.816 | 0.000 |  |
| Nelson Marlborough | -0.366 | 0.693 | 0.252 | 1.908 | 0.478 |  |
| South Canterbury | 0.676 | 1.967 | 0.749 | 5.167 | 0.170 |  |
| Southern | -1.152 | 0.316 | 0.115 | 0.869 | 0.026 |  |
| Tairawhiti | 1.861 | 6.428 | 2.989 | 13.824 | 0.000 |  |
| Taranaki | -0.155 | 0.857 | 0.311 | 2.357 | 0.764 |  |
| Waikato | 0.721 | 2.057 | 1.039 | 4.070 | 0.038 |  |
| Wairarapa | 0.653 | 1.921 | 0.657 | 5.621 | 0.233 |  |
| Waitemata | -3.487 | 0.031 | 0.004 | 0.239 | 0.001 |  |
| West Coast | -0.715 | 0.489 | 0.063 | 3.821 | 0.495 |  |
| Whanganui | 1.486 | 4.418 | 2.039 | 9.570 | 0.000 |  |
| Year |  |  |  |  |  |  |
| 1999 | Reference |  |  |  |  | **<0.001** |
| 2000 | -0.006 | 0.994 | 0.349 | 2.834 | 0.991 |  |
| 2001 | 0.987 | 2.683 | 1.128 | 6.381 | 0.026 |  |
| 2002 | 1.763 | 5.828 | 2.618 | 12.972 | 0.000 |  |
| 2003 | 0.713 | 2.041 | 0.832 | 5.005 | 0.119 |  |
| 2004 | 1.035 | 2.815 | 1.197 | 6.621 | 0.018 |  |
| 2005 | 0.687 | 1.988 | 0.811 | 4.876 | 0.133 |  |
| 2006 | 0.739 | 2.095 | 0.862 | 5.092 | 0.103 |  |
| 2007 | 0.522 | 1.686 | 0.673 | 4.226 | 0.265 |  |
| 2008 | 0.434 | 1.543 | 0.608 | 3.920 | 0.362 |  |
| 2009 | -0.452 | 0.637 | 0.202 | 2.006 | 0.441 |  |
| 2010 | 0.413 | 1.511 | 0.595 | 3.838 | 0.385 |  |
| 2011 | 0.559 | 1.750 | 0.706 | 4.335 | 0.227 |  |
| 2012 | 1.006 | 2.734 | 1.168 | 6.401 | 0.020 |  |
| 2013 | 0.104 | 1.110 | 0.413 | 2.980 | 0.836 |  |
| 2014 | -0.500 | 0.606 | 0.193 | 1.911 | 0.393 |  |
| 2015 | -0.050 | 0.951 | 0.345 | 2.622 | 0.922 |  |
| 2016 | -0.537 | 0.584 | 0.186 | 1.841 | 0.359 |  |
| 2017 | 0.537 | 1.710 | 0.697 | 4.195 | 0.241 |  |
| Year (continuous) ^#^ | -0.049 | 0.953 | 0.931 | 0.974 | 0.000 | **<0.001** |
| Polynomial(year, 3)1^##^ | -18.156 | 0.000 | 0.000 | 0.000 | 0.000 | **<0.001** |
| Polynomial(year, 3)2^##^ | -5.741 | 0.003 | 0.000 | 16.102 | 0.187 |  |
| Polynomial(year, 3)^###^ | 15.544 | 5.631e+6 | 1.028e+3 | 3.084e+10 | 0.000 |  |

** Total rainfall_1* is the total monthly rainfall in cm the month prior to the month a case is reported in.

*** Total rainfall_2* is the total monthly rainfall in cm in the month, two months prior to the month a case is reported in.

****Total rainfall_3* is the total monthly rainfall in cm in the month, three months prior to the month a case is reported in.

^#^Year as a linear variable.

^##^Year as a cuboid polynomial variable.

Supplementary Material S7: Supplementary tables of the model for Ballum cases

*Supplementary Table S22: Table of the summary statistics for the number of* Ballum *cases included in the analysis that were reported in New Zealand between 1999 and 2017 by District Health Board, season, and year.*

| Categorical variables | | | | | | | |
| --- | --- | --- | --- | --- | --- | --- | --- |
|  | **Number of cases per month** | | | | |  | **Total number of cases** |
|  | **Median** | **Mean (SD)** | **Interquartile range** | **Minimum value** | **Maximum value** | **Number of months** |  |
| Season |  |  |  |  |  |  |  |
| Autumn | 1.0 | 1.44 (±1.36) | 2.00 | 0 | 7 | 57 | 82 |
| Spring | 1.0 | 0.74 (±0.88) | 1.00 | 0 | 4 | 57 | 42 |
| Summer | 1.0 | 0.86 (±0.97) | 1.00 | 0 | 4 | 57 | 49 |
| Winter | 1.0 | 1.32 (±1.12) | 2.00 | 0 | 4 | 57 | 75 |
| District Health Board |  |  |  |  |  |  | |
| Auckland | 0.0 | 0.01 (±0.11) | 0.00 | 0 | 1 | 228 | 3 |
| Bay of Plenty | 0.0 | 0.06 (±0.23) | 0.00 | 0 | 1 | 228 | 13 |
| Canterbury | 0.0 | 0.07 (±0.25) | 0.00 | 0 | 1 | 228 | 15 |
| Capital and Coast | 0.0 | 0.01 (±0.11) | 0.00 | 0 | 1 | 228 | 3 |
| Counties Manukau | 0.0 | 0.01 (±0.09) | 0.00 | 0 | 1 | 228 | 2 |
| Hawke’s Bay | 0.0 | 0.07 (±0.26) | 0.00 | 0 | 1 | 228 | 17 |
| Hutt Valley | 0.0 | 0.01 (±0.09) | 0.00 | 0 | 1 | 228 | 2 |
| Lakes | 0.0 | 0.01 (±0.11) | 0.00 | 0 | 1 | 224 | 3 |
| Mid Central | 0.0 | 0.05 (±0.22) | 0.00 | 0 | 1 | 228 | 12 |
| Northland | 0.0 | 0.17 (±0.44) | 0.00 | 0 | 2 | 228 | 39 |
| Nelson Marlborough | 0.0 | 0.12 (±0.36) | 0.00 | 0 | 3 | 228 | 27 |
| South Canterbury | 0.0 | 0.03 (±0.16) | 0.00 | 0 | 1 | 228 | 6 |
| Southern | 0.0 | 0.07 (±0.26) | 0.00 | 0 | 1 | 228 | 17 |
| Tairawhiti | 0.0 | 0.01 (±0.11) | 0.00 | 0 | 1 | 228 | 3 |
| Taranaki | 0.0 | 0.07 (±0.26) | 0.00 | 0 | 1 | 228 | 17 |
| Waikato | 0.0 | 0.18 (±0.45) | 0.00 | 0 | 2 | 228 | 40 |
| Wairarapa | 0.0 | 0.03 (±0.17) | 0.00 | 0 | 1 | 228 | 7 |
| Waitemata | 0.0 | 0.01 (±0.11) | 0.00 | 0 | 1 | 228 | 3 |
| West Coast | 0.0 | 0.04 (±0.21) | 0.00 | 0 | 1 | 228 | 10 |
| Whanganui | 0.0 | 0.04 (±0.22) | 0.00 | 0 | 2 | 228 | 9 |
| Year |  |  |  |  |  |  |  |
| 1999 | 0.0 | 0.92 (±1.31) | 2.00 | 0 | 4 | 12 | 11 |
| 2000 | 1.0 | 0.83 (±0.94) | 1.00 | 0 | 3 | 12 | 10 |
| 2001 | 1.0 | 0.83 (±0.94) | 1.00 | 0 | 3 | 12 | 10 |
| 2002 | 0.0 | 0.75 (±0.97) | 2.00 | 0 | 2 | 12 | 9 |
| 2003 | 1.0 | 1.08 (±0.90) | 0.50 | 0 | 3 | 12 | 13 |
| 2004 | 0.0 | 0.08 (±0.29) | 0.00 | 0 | 1 | 12 | 1 |
| 2005 | 0.0 | 0.42 (±0.79) | 0.25 | 0 | 2 | 12 | 5 |
| 2006 | 1.0 | 1.33 (±1.07) | 1.25 | 0 | 3 | 12 | 16 |
| 2007 | 1.0 | 0.83 (±0.72) | 1.00 | 0 | 2 | 12 | 10 |
| 2008 | 1.0 | 1.50 (±1.00) | 1.00 | 0 | 4 | 12 | 18 |
| 2009 | 1.0 | 1.17 (±0.72) | 1.00 | 0 | 2 | 12 | 14 |
| 2010 | 1.5 | 1.67 (±1.37) | 2.25 | 0 | 4 | 12 | 20 |
| 2011 | 1.0 | 1.50 (±1.17) | 1.00 | 0 | 4 | 12 | 18 |
| 2012 | 1.5 | 1.50 (±1.45) | 2.00 | 0 | 4 | 12 | 18 |
| 2013 | 1.0 | 1.17 (±0.94) | 1.25 | 0 | 3 | 12 | 14 |
| 2014 | 1.0 | 0.58 (±0.51) | 1.00 | 0 | 1 | 12 | 7 |
| 2015 | 1.5 | 1.25 (±1.22) | 2.00 | 0 | 3 | 12 | 15 |
| 2016 | 1.0 | 1.42 (±1.24) | 1.25 | 0 | 4 | 12 | 17 |
| 2017 | 1.5 | 1.83 (±1.95) | 1.50 | 0 | 7 | 12 | 22 |

*Supplementary Table 23: Summary of average monthly incidence (number of cases/100 000 people) of reported human* Ballum *leptospirosis cases included in the analysis in each New Zealand District Health Board during the period 1 January 1999 to 31 December 2017.*

| District Health Board | Median | Mean | Standard deviation | Interquartile range | Minimum | Maximum |
| --- | --- | --- | --- | --- | --- | --- |
| Auckland | 0.00 | 0.00 | 0.03 | 0.00 | 0.00 | 0.24 |
| Bay of Plenty | 0.00 | 0.03 | 0.11 | 0.00 | 0.00 | 0.50 |
| Canterbury | 0.00 | 0.01 | 0.05 | 0.00 | 0.00 | 0.23 |
| Capital and Coast | 0.00 | 0.00 | 0.04 | 0.00 | 0.00 | 0.40 |
| Counties Manukau | 0.00 | 0.00 | 0.02 | 0.00 | 0.00 | 0.20 |
| Hawke's Bay | 0.00 | 0.05 | 0.17 | 0.00 | 0.00 | 0.68 |
| Hutt Valley | 0.00 | 0.01 | 0.06 | 0.00 | 0.00 | 0.68 |
| Lakes | 0.00 | 0.01 | 0.11 | 0.00 | 0.00 | 0.99 |
| MidCentral | 0.00 | 0.03 | 0.13 | 0.00 | 0.00 | 0.62 |
| Nelson Marlborough | 0.00 | 0.09 | 0.26 | 0.00 | 0.00 | 1.96 |
| Northland | 0.00 | 0.11 | 0.28 | 0.00 | 0.00 | 1.39 |
| South Canterbury | 0.00 | 0.05 | 0.28 | 0.00 | 0.00 | 1.86 |
| Southern | 0.00 | 0.02 | 0.09 | 0.00 | 0.00 | 0.35 |
| Tairawhiti | 0.00 | 0.03 | 0.25 | 0.00 | 0.00 | 2.17 |
| Taranaki | 0.00 | 0.07 | 0.23 | 0.00 | 0.00 | 0.94 |
| Waikato | 0.00 | 0.05 | 0.12 | 0.00 | 0.00 | 0.61 |
| Wairarapa | 0.00 | 0.07 | 0.42 | 0.00 | 0.00 | 2.53 |
| Waitemata | 0.00 | 0.00 | 0.02 | 0.00 | 0.00 | 0.21 |
| West Coast | 0.00 | 0.13 | 0.63 | 0.00 | 0.00 | 3.22 |
| Whanganui | 0.00 | 0.06 | 0.34 | 0.00 | 0.00 | 3.17 |

*.*

*Supplementary Table 24: Table showing the results of univariable analyses measuring the crude association between the explanatory variables and the incidence rate of reported* Ballum *cases included in the analysis in New Zealand in the period 1 January 1999 to 31 December 2017 using Poisson models.*

| Variable name | Beta Coefficient | Incidence rate ratio (IRR) | IRR Confidence Interval  2.5% 97.5% | | Wald test  p-value | Likelihood ratio test  p-value |
| --- | --- | --- | --- | --- | --- | --- |
| Animal density (animals/km2) | 0.001 | 1.001 | 1.000 | 1.003 | 0.06 | **0.06** |
| Total monthly Rainfall (cm) | | |  |  |  |  |
| Total rainfall | 0.033 | 1.033 | 1.015 | 1.052 | **<0.001** | **<0.001** |
| Total rainfall_1* | 0.047 | 1.049 | 1.031 | 1.066 | **<0.001** | **<0.001** |
| Total rainfall_2** | 0.030 | 1.030 | 1.012 | 1.049 | **<0.01** | **<0.01** |
| Total rainfall_3** | 0.031 | 1.031 | 1.013 | 1.050 | **<0.01** | **<0.01** |
| Season |  |  |  |  |  |  |
| Autumn | Reference | |  |  |  | **<0.001** |
| Spring | -0.668 | 0.513 | 0.353 | 0.744 | **<0.001** |  |
| Summer | -0.514 | 0.598 | 0.420 | 0.852 | **<0.01** |  |
| Winter | -0.089 | 0.915 | 0.669 | 1.251 | 0.58 |  |
| District health board | | |  |  |  |  |
| Northland | Reference | |  |  |  | **<0.001** |
| Auckland | -3.580 | 0.028 | 0.009 | 0.090 | **<0.001** |  |
| Bay of Plenty | -1.363 | 0.256 | 0.137 | 0.479 | **<0.001** |  |
| Canterbury | -2.087 | 0.124 | 0.068 | 0.225 | **<0.001** |  |
| Capital and Coast | -3.143 | 0.043 | 0.013 | 0.140 | **<0.001** |  |
| Counties Manukau | -4.047 | 0.017 | 0.004 | 0.072 | **<0.001** |  |
| Hawke's Bay | -0.812 | 0.444 | 0.251 | 0.785 | **<0.01** |  |
| Hutt Valley | -2.864 | 0.057 | 0.014 | 0.236 | **<0.001** |  |
| Lakes | -2.117 | 0.120 | 0.037 | 0.389 | **<0.001** |  |
| MidCentral | -1.232 | 0.292 | 0.153 | 0.557 | **<0.001** |  |
| Nelson Marlborough | -0.223 | 0.800 | 0.490 | 1.307 | 0.37 |  |
| South Canterbury | -0.839 | 0.432 | 0.183 | 1.021 | 0.06 |  |
| Southern | -1.472 | 0.230 | 0.130 | 0.406 | **<0.001** |  |
| Tairawhiti | -1.346 | 0.260 | 0.080 | 0.842 | **<0.05** |  |
| Taranaki | -0.474 | 0.622 | 0.352 | 1.100 | 0.10 |  |
| Waikato | -0.801 | 0.449 | 0.289 | 0.698 | **<0.001** |  |
| Wairarapa | -0.372 | 0.690 | 0.308 | 1.542 | 0.36 |  |
| Waitemata | -3.750 | 0.024 | 0.007 | 0.076 | **<0.001** |  |
| West Coast | 0.226 | 1.254 | 0.626 | 2.512 | 0.52 |  |
| Whanganui | -0.569 | 0.566 | 0.274 | 1.169 | 0.12 |  |
| Year |  |  |  |  |  |  |
| 1999 | Reference | |  |  |  | **<0.01** |
| 2000 | -0.101 | 0.904 | 0.384 | 2.128 | 0.817 |  |
| 2001 | -0.107 | 0.898 | 0.382 | 2.116 | 0.806 |  |
| 2002 | -0.230 | 0.795 | 0.329 | 1.918 | 0.609 |  |
| 2003 | 0.118 | 1.125 | 0.504 | 2.512 | 0.773 |  |
| 2004 | -2.462 | 0.085 | 0.011 | 0.660 | **<0.05** |  |
| 2005 | -0.863 | 0.422 | 0.147 | 1.214 | 0.109 |  |
| 2006 | 0.287 | 1.333 | 0.619 | 2.872 | 0.463 |  |
| 2007 | -0.192 | 0.825 | 0.351 | 1.944 | 0.661 |  |
| 2008 | 0.387 | 1.473 | 0.696 | 3.119 | 0.311 |  |
| 2009 | 0.126 | 1.134 | 0.515 | 2.499 | 0.754 |  |
| 2010 | 0.472 | 1.603 | 0.768 | 3.345 | 0.209 |  |
| 2011 | 0.359 | 1.431 | 0.676 | 3.031 | 0.349 |  |
| 2012 | 0.353 | 1.424 | 0.672 | 3.014 | 0.356 |  |
| 2013 | 0.094 | 1.099 | 0.499 | 2.420 | 0.815 |  |
| 2014 | -0.616 | 0.540 | 0.209 | 1.394 | 0.203 |  |
| 2015 | 0.126 | 1.134 | 0.521 | 2.470 | 0.751 |  |
| 2016 | 0.235 | 1.264 | 0.592 | 2.700 | 0.544 |  |
| 2017 | 0.468 | 1.596 | 0.774 | 3.292 | 0.205 |  |
| Year (continuous)^#^ | 0.029 | 1.030 | 1.006 | 1.054 | **<0.05** | **<0.05** |
| Poly (yearcontinuous, 3)1^##^ | 10.859 | 52012.340 | 9.210 | 2.94E+08 | **<0.05** | 0.06 |
| Poly (yearcontinuous, 3)2^##^ | 0.336 | 1.399 | 0.000 | 7436.729 | 0.939 |  |
| Poly (yearcontinuous, 3)3^##^ | -4.308 | 0.013 | 2.76E-06 | 65.606 | 0.320 |  |

** Total rainfall_1* is the total monthly rainfall in cm the month prior to the month a case is reported in.

*** Total rainfall_2* is the total monthly rainfall in cm in the month, two months prior to the month a case is reported in.

****Total rainfall_3* is the total monthly rainfall in cm in the month, three months prior to the month a case is reported in.

^#^Year as a linear variable.

^##^Year as a cuboid polynomial variable.

1. (New Zealand Ministry of Health, 2021) [↑](#footnote-ref-1)
2. The land areas are sourced from a GIS dataset called ‘Territorial Authority 2015_V1_00 Clipped’ of territorial authority boundaries for 2015 as defined by the Local Government Commission and/or the territorial authorities themselves but maintained by Statistics New Zealand. . ISO 19115/19139, Dublin Core. 26 July 2017. [NZGD2000 / New Zealand Transverse Mercator 2000](http://epsg.io/2193).

   WD = Territorial Authority withdrawn [↑](#footnote-ref-2)
